# Clinical, safety, and patient-reported outcomes associated with second-line and later therapies in primary biliary cholangitis: A systematic review

**DOI:** 10.1097/HC9.0000000000001048

**Published:** 2026-10-14

**Authors:** Marco Carbone, David Jones, Oyinkan Solanke, Rikke Brandt, Taryn Krause, Darren Asquith, Alex Pashley, Sonia Shaw, Emma Worthington, Jörn M. Schattenberg

**Affiliations:** 1 Division of Gastroenterology and Center for Autoimmune Liver Diseases, Department of Medicine and Surgery, University of Milan-Bicocca, Milan, Italy; 2 Institute of Cellular Medicine and NIHR Newcastle Biomedical Research Centre, Newcastle University, Newcastle Upon Tyne, UK; 3 Ipsen, London, UK; 4 Costello Medical, Cambridge, UK; 5 Department of Internal Medicine II, Saarland University Medical Center, Homburg, Germany; 6 Pharma Science Hub Saarbrücken, Germany

**Keywords:** biliary, biological markers, cholangitis, clinical decision-making, fibrosis, pruritus, research design, second-line therapy

## Abstract

**Background:**

Primary biliary cholangitis (PBC) is a rare, chronic, cholestatic disease that can result in cirrhosis, liver failure, and death. While biochemical markers such as alkaline phosphatase (ALP) and total bilirubin (TB) measure disease activity and progression, definitions of biochemical response vary. This systematic literature review (SLR) identified clinical studies for investigational and approved treatments in patients with PBC with inadequate response or intolerance to ursodeoxycholic acid requiring second-line (2L) or later (2L+) therapies.

**Methods:**

Electronic databases were searched on August 7, 2025 (MEDLINE; Embase) or September 30, 2025 (Cochrane Database of Systematic Reviews; CENTRAL), and conference proceedings (2021–2025), bibliographies, and ClinicalTrials.gov were searched on August 15, 2025, following a pre-specified protocol (PROSPERO ID: CRD42023382262). Interventional and observational studies in the 2L/2L+ setting were prioritized for inclusion, and randomized controlled trials for extraction.

**Results:**

A total of 149 publications on 44 unique studies were included. Phase III trials evaluated elafibranor, seladelpar, budenoside, bezafibrate, and obeticholic acid. Various composite biochemical response endpoints were reported across 34% of studies, most frequently the POISE criteria (18%). Biochemical markers were assessed frequently (ALP: 89%; TB: 71%); however, upper limits of normal and response thresholds varied substantially. Response rates in placebo-controlled trials favored active treatment, although placebo responses were highly variable. Non-invasive fibrosis measures were reported in 16% of studies and showed small short-term changes. Patient-reported outcomes assessed pruritus (52%) and fatigue (32%) using a variety of scales.

**Conclusion:**

The evidence base for 2L/2L+ PBC therapies is extensive but heterogeneous in study designs, patient populations, and treatment assessment scales and outcome definitions, limiting cross-trial comparisons. This SLR provides a robust foundation for contextualizing study outcomes within the broader landscape and supporting clinical decision-making.

## INTRODUCTION

Primary biliary cholangitis (PBC) is a rare, chronic, cholestatic liver disease characterized by the autoimmune destruction of intrahepatic bile ducts, resulting in cirrhosis and fibrosis.^1^,^2^ The pathology of PBC is largely unknown, and both environmental and genetic factors have been associated with increased risk of developing the disease.

^3^ PBC predominantly affects women aged ≥40 years, and while estimates of global PBC prevalence vary greatly, ranging from 1.91 to 40.2 per 100,000 individuals, global prevalence has increased over time.^1^,^4^,^5^,^6^,^7^ Fatigue and pruritus are the most common symptoms of PBC and can significantly reduce health-related quality of life (HRQoL).^8^ In addition, patients with PBC may be at a higher risk of cardiovascular, gastrointestinal, bone, and mental health disorders compared with the general population.^9^

Diagnosis of PBC is based on the presence of liver tests indicative of cholestatic hepatitis, particularly elevated levels of alkaline phosphatase (ALP), as well as serological positivity for anti-mitochondrial antibodies (AMA).^1^ Biochemical markers of cholestasis, such as elevated ALP, total bilirubin (TB), gamma-glutamyl transferase (GGT), alanine aminotransferase (ALT), and aspartate transaminase (AST), are used individually or as composite measures in clinical trials and are key indicators of disease stage and progression.^10^,^11^,^12^ In particular, ALP and TB are increasingly being recognized as markers to predict long-term outcomes in PBC, linked to an increased risk of liver transplantation and death,^10^,^11^ and often serve as primary efficacy endpoints in clinical trials of emerging PBC therapies.^1^,^10^,^13^ Beyond these biochemical markers, assessment of liver fibrosis and cirrhosis is required to fully understand the effects of individual treatments.

Ursodeoxycholic acid (UDCA) is currently the only approved first-line (1L) treatment for PBC. However, its biochemical efficacy is variable, with around 40% of patients experiencing an inadequate response that does not effectively prevent disease progression or poor clinical outcomes, and 3%–5% of patients have intolerable side effects.^12^,^14^,^15^ For patients who have an inadequate response to, or are intolerant of UDCA, treatments used in the second-line (2L) and later (2L+) settings include obeticholic acid (OCA) and peroxisome proliferator-activated receptor (PPAR) agonists [elafibranor and seladelpar, and unlicensed fibrates (eg, fenofibrate and bezafibrate)].^1^,^16^,^17^ Both elafibranor and seladelpar have been recently approved as 2L treatments for PBC in several regions.^18^,^19^,^20^,^21^,^22^,^23^,^24^ These approvals were based on results of the phase III ELATIVE and RESPONSE trials, respectively, representing the first approved treatments in PBC in ~8 years.^15^,^25^,^26^ Before this, OCA, a selective farnesoid X receptor agonist, was approved as a 2L treatment for PBC in 2016.^27^,^28^ However, in 2024, the European Medicines Agency (EMA) concluded that the available evidence did not sufficiently confirm that the benefits of OCA outweigh its risks, resulting in its marketing authorization being revoked in the European Union (EU).^29^,^30^,^31^ OCA has since been voluntarily withdrawn from the United States, following a request from the U.S. Food and Drug Administration (FDA).^32^

There are currently a number of definitions of biochemical response that are used in clinical trial settings and in real-world practice in PBC. In addition, several patient-reported outcomes (PROs) are used to assess changes in critical components of PBC, including fatigue, pruritus, and HRQoL. Therefore, the evidence across different aspects of treatment effects and how they have been measured needs to be considered together to decipher any differences in therapeutic profiles and to evaluate potential benefits of PBC therapies, highlighting the need for comprehensive, evidence-based frameworks to inform decision-making. This systematic literature review (SLR) aimed to identify and provide a comprehensive evidence base of currently available clinical efficacy and safety data for investigational and approved treatments in the 2L/2L+ settings.

## METHODS

### Literature searches

This SLR was conducted with a pre-specified protocol, registered on PROSPERO (ID: CRD42023382262), following current best practices as detailed in the University of York Centre for Reviews and Dissemination’s (CRD) Guidance for Undertaking Reviews in Health Care.^33^ The original SLR was conducted in November 2022 and subsequently underwent 3 updates (in December 2023, June 2024, and August/September 2025, respectively).

Electronic databases were searched from their inception date (Supplemental Tables S1–S7, https://links.lww.com/HC9/C483), and hand-searching of key conference proceedings (from 2021 to 2025) and the bibliographies of any relevant systematic reviews and (network) meta-analyses (NMAs) were carried out. Conference proceedings before 2021 were not searched on the assumption that high-quality research would have since been published in full in a peer-reviewed journal.

For the original SLR, MEDLINE and Embase were searched via the OvidSP platform. The Cochrane Database of Systematic Reviews (CDSR) and the Cochrane Central Register of Controlled Trials (CENTRAL) were searched through the Cochrane Library via the Wiley Online platform, and the Database of Abstracts of Reviews of Effects (DARE) was searched via the University of York CRD platform. All searches were conducted on November 21, 2022. For SLR Update I, MEDLINE and Embase were searched separately via the OvidSP Platform; CDSR and CENTRAL were searched through the Cochrane Library via the Wiley Online platform on December 18, 2023. For SLR Update II, MEDLINE, Embase, CDSR, and CENTRAL were searched simultaneously on June 19, 2024, via the OvidSP platform. For SLR Update III, MEDLINE and Embase were searched via the OvidSP Platform on August 7, 2025, and CDSR and CENTRAL were searched via the OvidSP Platform on September 30, 2025.

Supplementary searches of conferences, reference lists, and ClinicalTrials.gov were also conducted (Supplemental Tables S8 and S9, https://links.lww.com/HC9/C483).

### Eligibility criteria

Inclusion and exclusion criteria were developed in line with the Population, Intervention, Comparator, Outcomes (PICO) framework, as detailed in Table 1. Due to the high volume of publications identified, a two-stage prioritization strategy was applied to focus on the extraction of the evidence needed to support a health technology assessment (HTA) submission for elafibranor in PBC, by focusing on randomized controlled trials in the 2L/2L+ setting (Table 1). Each abstract and full text was screened against the eligibility criteria by 2 independent reviewers, and any disagreements were resolved by discussion, with a third reviewer if necessary, until a final decision was made.

**TABLE 1:** Eligibility criteria for included publications

Domain	Inclusion criteria	Exclusion criteria
Population	Adult patients (aged ≥18 y) with PBC	• Patients <18 y old • Patients without PBC
Intervention	• Monotherapy or combination therapy in any treatment line, including, but not limited to: • Elafibranor • Ursodeoxycholic acid • Obeticholic acid • Fibrates • Seladelpar • Other relevant molecules, eg, linerixibat, saroglitazar, volixibat, CNP-104, Setanaxib, K-808, TH-104	No planned intervention
Comparators	• Best supportive care • Placebo • Any other comparators (or none)	NA
Outcomes	• Clinical efficacy/effectiveness outcomes, including but not limited to: • Survival outcomes • Response rates • Biochemical responses (eg, CfB in ALP levels, etc.) • Pruritus • Fatigue Safety outcomes, including but not limited to: • AEs (including TRAEs, serious AEs, fatigue and fractures) • Deaths • Discontinuation due to AEs/TRAEs Pruritus-specific health-related quality of life outcome measures such as • ItchyQoL	• Studies not reporting any relevant outcomes, eg, pharmacokinetics • Studies reporting a relevant outcome but in a mixed population where results for PBC are not reported separately
Study design	• RCTs assessing any intervention • Non-randomized interventional studies assessing elafibranor • Non-randomized interventional studies (eg, single-arm trials) featuring any other interventions within scope • Observational studies *Due to the high volume of identified evidence, a two-stage prioritization strategy was ultimately implemented.* *Interventional and observational studies in the 2L/2L+ setting were prioritized following the title/abstract review stage (all other observational studies were deprioritized and not reviewed at the full-text stage)* *Any studies of elafibranor, and RCTs in the 2L/2L+ setting were then prioritized for extraction (all observational studies unless reporting on elafibranor were deprioritized for extraction)*	• Non-primary research publications, including: • Narrative reviews • Editorials • Guidelines • Commentaries • Opinion pieces
		Relevant SLRs and (N)MAs were eligible at the title/abstract review stage and hand-searched for relevant primary studies, but were excluded during the full-text review stage unless they reported primary research	
Other considerations	• Peer-reviewed literature or congress abstracts published in or after 2021 • Studies on human subjects • Any geography • Studies with an abstract or full-text published in the English language	• Peer-reviewed literature or congress abstracts published before 2021 • In vitro/animal studies

Abbreviations: 2L, second-line; AEs, adverse events; ALP, alkaline phosphatase; CfB, change from baseline; NA, not applicable; NMAs, network meta-analyses; PBC, primary biliary cholangitis; RCTs, randomized controlled trials; SAE, serious adverse event; SLRs, systematic literature reviews; TRAEs, treatment-related adverse events.

### Data extraction and quality assessment

Information and data, including study designs and characteristics, patient characteristics, efficacy, safety, liver fibrosis, and symptom-related and HRQoL-related PROs, were extracted into a pre-specified Microsoft Excel grid, in line with the University of York CRD guidelines.^33^ Absolute and relative change from baseline values and *p*-values were extracted, where reported.

The quality of all included studies was assessed using the quality assessment tool developed by the University of York CRD for randomized controlled trials (RCTs).^34^

Data extraction and quality assessment processes were conducted by a single reviewer and verified by a second reviewer. Any discrepancies were resolved by discussion, with a third reviewer if necessary, until a consensus was reached.

## RESULTS

A summary of the key findings is presented in Figure 1.

**FIGURE 1:**
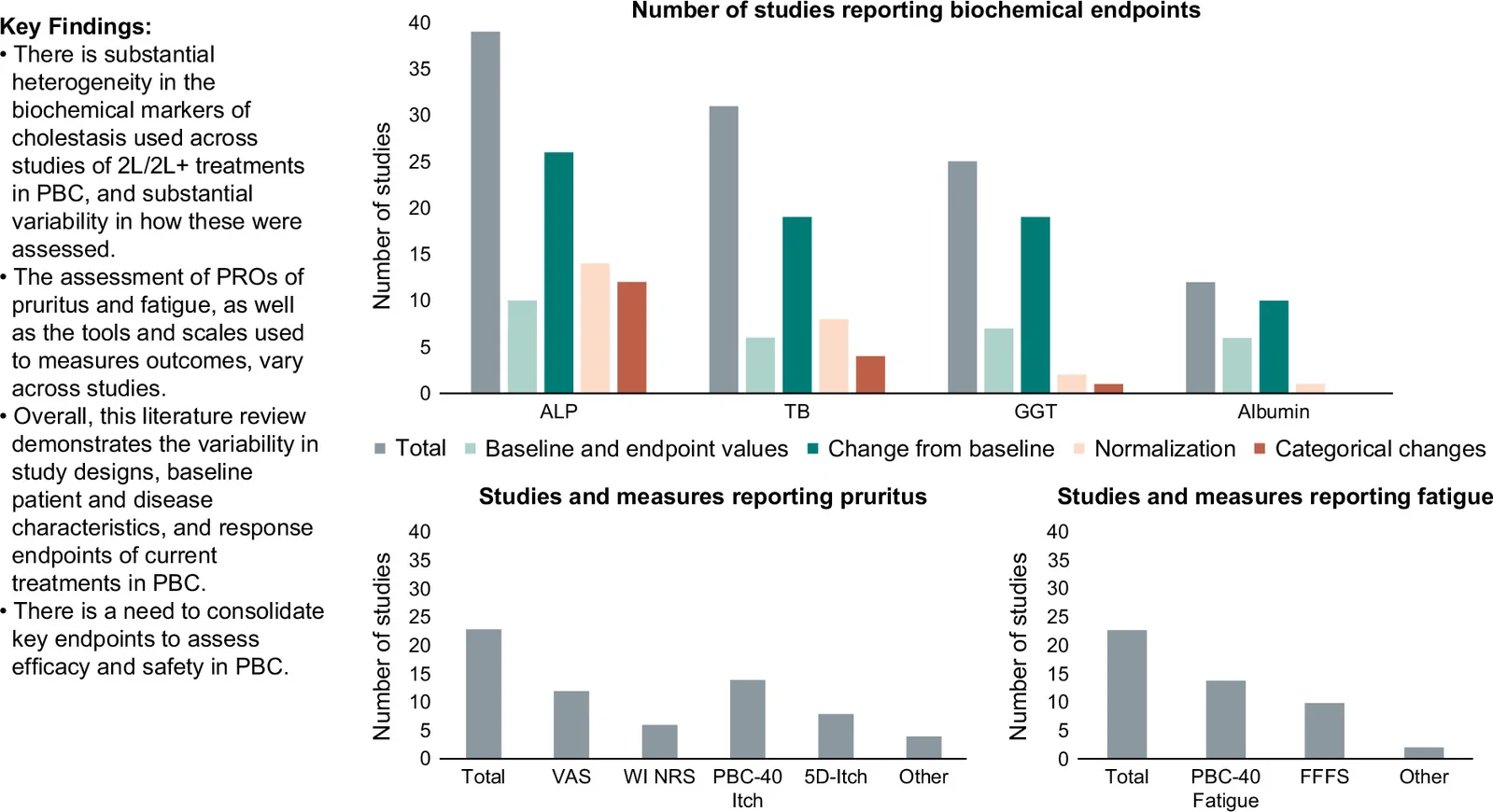
Summary of key findings. Abbreviations: 5D, 5-dimensional; ALP, alkaline phosphatase; FFS, Fisk Fatigue Severity Scale; GGT, gamma-glutamyl transferase; PBC, primary biliary cholangitis; TB, total bilirubin; VAS, visual analog scale; WI, worst itch.

### Searches and screening

The results of each stage of the screening process, including the 3 SLR updates, are presented in Figure 2. Due to the large volume of relevant records included at this stage, records reporting on interventional and observational studies in the 2L/2L+ were prioritized for inclusion, and RCTs in the 2L/2L+ were prioritized for extraction in the original SLR and updates (Table 1). All other observational studies were deprioritized for extraction.

**FIGURE 2:**
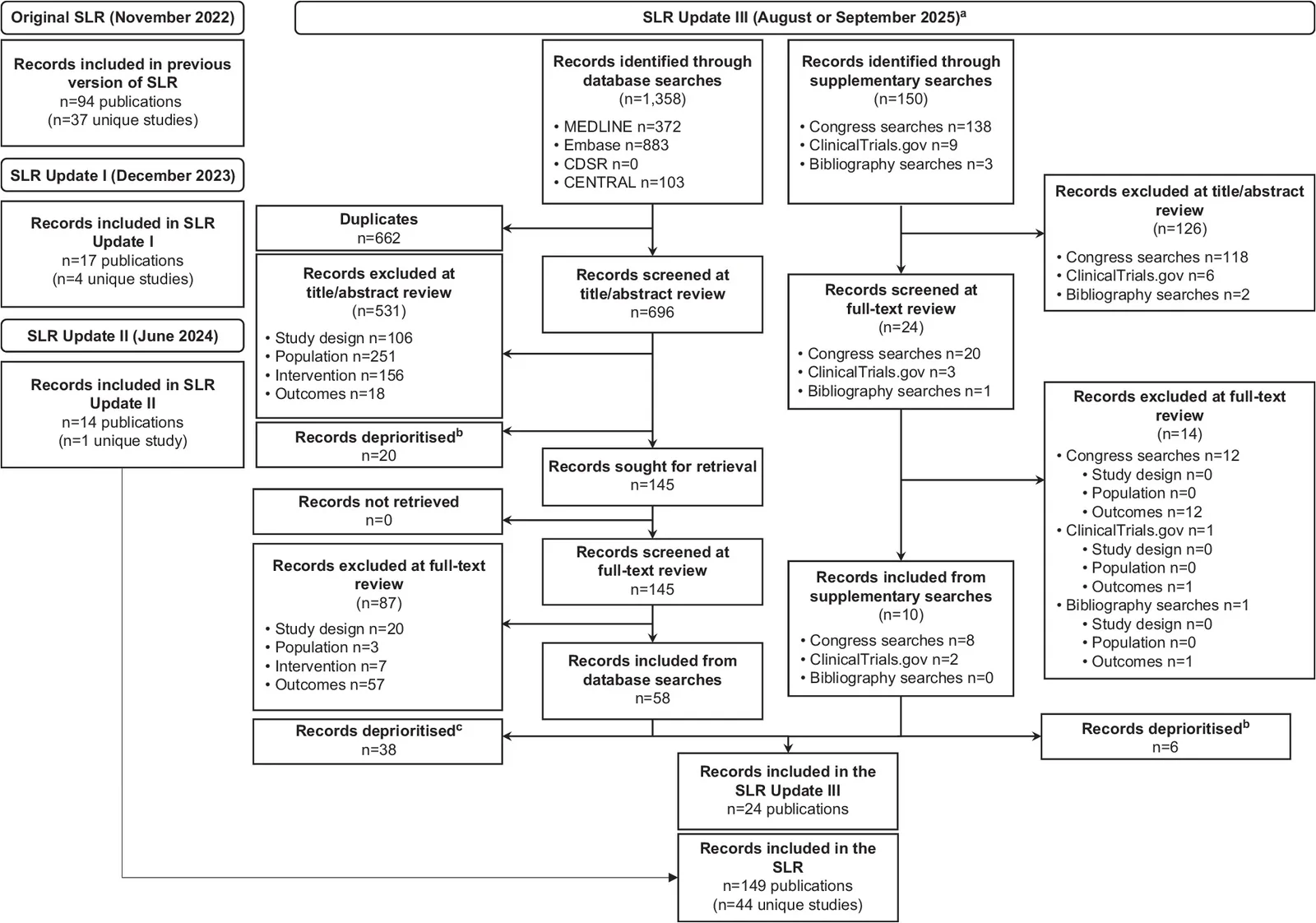
PRISMA flow diagram for studies identified in the SLR. ^a^MEDLINE and EMBASE were searched on August 7, 2025. CDSR and CENTRAL were searched on September 30, 2025. ^b^Observational studies not conducted in the 2L/2L+ setting (ie, studies of 1L UDCA, mixed treatment settings) were deprioritized and not reviewed at the full-text stage. ^c^RCTs not conducted in the 2L/2L+ treatment setting and all observational studies (unless reporting on elafibranor) were deprioritized for extraction. Abbreviations: CDSR, Cochrane Database of Systematic Reviews; CENTRAL, Cochrane Central Register of Controlled Trials; SLR, systematic literature review.

Overall, 149 publications reporting on 44 unique studies were prioritized for extraction in the SLR; a full list of included studies is included in Supplemental Table S10, https://links.lww.com/HC9/C483. In the original SLR, 353 publications were included, of which 94 publications reporting on 36 unique studies were prioritized for extraction. In SLR Update I, 48 additional publications were included, of which 17 novel publications reporting on 5 unique studies were prioritized for extraction. In SLR Update II, a total of 42 additional publications were included, of which 14 publications reporting on 1 unique study and 6 studies already captured in the SLR were prioritized for extraction. In SLR Update III, a total of 68 additional publications were included, of which 24 publications reporting on 2 novel studies and 6 studies already captured in the SLR were prioritized for extraction (Figure 2).

### Study characteristics

An overview of the designs of the prioritized studies included in the SLR is presented in Table 2. In line with the prioritization strategy, all studies were RCTs in the 2L/2L+ treatment setting (n=44), of which 33 (75.6%) were published after 2010. Among the studies included, 20 were multi-center across multiple countries, 11 were multi-center within a single country, 5 were single-center, and the remaining 8 did not report study location. Seventeen studies were phase II, 6 were phase III, 3 were phase IV studies, and 18 did not report the study phase.

**TABLE 2:** Summary of studies prioritized for extraction in the SLR

Study	Study design	Country and setting	Population	Intervention arm(s)	Comparator arm
Studies of UDCA add-on therapy					
Askari, et al^35^	Double-blind RCT (Phase: NR)	Single-center US	Patients treated with ≥13 mg/kg UDCA for ≥3 mo and an ALP 1.05×ULN	Tetrathiomolybdate (120 mg initially, escalating to 180 mg) + UDCA (N=13)	Placebo + UDCA (N=15)
BEZURSO (NCT01654731)^36^	Phase 3 double-blind RCT	Multi-center France	Adult patients with PBC, with incomplete response (defined as ALP >1.5×N, AST >1.5×N, or total bilirubin >17 µmol/L) after ≥3 mo of UDCA at 13−15 mg/kg/d	Bezafibrate 400 mg + UDCA (N=50)	Placebo + UDCA (N=50)
Combes, et al^37^	Double-blind RCT (Phase: NR)	Multi-center US	Patients with a diagnosis of chronic cholestatic liver disease of ≥6 mo duration and on UDCA ≥6 mo	Methotrexate + UDCA (Disease stages I–II, N=62; stages III–IV, N=70)	Placebo + UDCA (stages I–II, N=64; stages III–IV, N=69)
Gonzalez-Koch, et al^38^	Double-blind RCT (Phase: NR)	Setting NR	Patients with ALP or GGT levels ≥1.5×ULN. Patients with a positive test for AMAs, biopsy-proven PBC, biliary tract patency, and elevated immunoglobulin M	Methotrexate + UDCA (N=13)	Placebo + UDCA (N=12)
HAART (NCT01614405)	Double-blind RCT (Phase: NR)	Multi-center Canada	Patients with PBC >18 y of age with a lack of response to UDCA after ≥6 mo of therapy, defined as elevated ALP >2×ULN or abnormal bilirubin	Tenofovir 300 mg + emtricitabine 200 mg + lopinavir/ritonavir (800/200 mg) + UDCA (N=5)	Placebo + UDCA (N=8)
Hirschfield, et al^39^ (NCT00550862)	Double-blind RCT (Phase: NR)	International, multi-center Austria, Canada, France, Germany, Netherlands, Spain, UK, US	Patients aged 18−70 y with a stable dose of UDCA for ≥6 mo before screening, proven or likely diagnosed as PBC	OCA 10 mg + UDCA (N=38) OCA 25 mg + UDCA (N=48) OCA 50 mg + UDCA (N=41)	Placebo + UDCA (N=38)
Hirschfield, et al(NCT00746486)^40^	Phase 3 double-blind RCT	International, multi-center 13 countries (with 6 active sites in Germany, 3 in Denmark and 2 in Austria)	Patients aged ≥18 y with a biopsy-proven diagnosis of PBC who received UDCA treatment for ≥6 mo and had an incomplete response to UDCA treatment	Budesonide + UDCA (N=40)	Placebo + UDCA (N=22)
Hosonuma, et al^41^	Open-label RCT (Phase: NR)	Muti-center Japan	PBC patients with dyslipidemia	Bezafibrate 400 mg + UDCA (N=13)	UDCA (N=14)
Ikeda, et al^42^	RCT (Phase and Blinding: NR)	Setting NR Japan	Patients diagnosed with PBC, first treated with UDCA for 30 mo	Colchicine 1 mg + UDCA (N=10)	UDCA (N=12)
Iwasaki, et al^43^	RCT (Phase and Blinding: NR)	Multi-center Japan	Patients with medical history and laboratory tests consistent with chronic cholestatic liver disease. Patients with positive AMA or antipyruvate dehydrogenase complex; ALP elevation of ≥1.5×ULN, and had imaging that showed the absence of biliary tract obstruction and hyperlipoproteinemia	Bezafibrate 400 mg + UDCA (N=12)	UDCA (N=10)
Jones, et al^44^ (NCT02609048)	Phase 2 double-blind RCT	International, multi-center Canada, Germany, Poland, UK, US	Patients aged 18−75 y with a diagnosis of PBC. Patients were required to be on a stable and recommended dose of UDCA for 12 mo and have ALP of ≥1.67×ULN	Seladelpar 50 mg (N=13) Seladelpar 200 mg + UDCA (N=12)	Placebo + UDCA (N=13)
Kanda, et al^45^	RCT (Phase and Blinding: NR)	Setting NR Japan	Patients diagnosed with PBC histologically through liver biopsy, who had been treated with 600 mg/d of UDCA for ≥6 mo, and had elevated ALP [>ULN (359 IU/L)] despite receiving UDCA	Bezafibrate 400 mg + UDCA (N=11)	UDCA (N=11)
Kurihara, et al^46^	RCT (Phase and Blinding: NR)	Setting NR	Patients diagnosed with PBC through liver biopsy	Bezafibrate 400 mg + UDCA (N=12)	UDCA (N=12)
Li 2022 (ChiCTR1800020160)	Open-label RCT (Phase: NR)	Single-center China	Patients aged 18−65 y, diagnosed as PBC by the AASLD in 2020. Patients with clinical stages 2 and 3 (ie, abnormal liver function and symptomatic phase) and patients with abnormal ALP and/or GGT after 6 mo of treatment with UDCA	Fenofibrate 200 mg + UDCA (N=24)	UDCA (N=24)
Liberopoulos^47^	Open-label RCT (Phase: NR)	Setting NR	Histologically confirmed PBC (stages I and II), with persistent elevations of liver enzyme activities (specifically ALP >3×ULN) after treatment with UDCA (600 mg/d) for ≥8 mo	Fenofibrate 200 mg + UDCA (N=6)	UDCA (N=4)
Lim, et al^48^	RCT	Single-center The Netherlands	Patients with positive AMA and liver biopsies consistent with PBC	Azathioprine 50 mg + prednisone + UDCA (N=54)	Placebo + UDCA (N=55)
Mayo (NCT02026401)^49^	Phase 2 double-blind RCT	International, multi-center Australia, US	Patients with PBC who had an inadequate response to UDCA. Patients had an ALP level of ≥1.67×ULN, total bilirubin ≤2×ULN, and ALT and AST ≤10×ULN	Aldafermin (NGM282) 0.3 mg + UDCA (N=14) Aldafermin (NGM282) 3 mg + UDCA (N=16)	Placebo + UDCA (N=15)
Nakai, et al^50^	RCT (Phase: NR)	Setting NR	NR	Bezafibrate 400 mg + UDCA (N=13)	UDCA (N=10)
NCT03226067	Phase 2 double-blind RCT	International, multi-center Belgium, Canada, Germany, Greece, Israel, Italy, Spain, UK, US	Patients aged 18−80 y diagnosed with PBC, with serum ALP ≥1.5×ULN and serum GGT ≥1.5×ULN. Patients were on UDCA treatment for ≥6 mo and a stable dose for ≥3 mo	Setanaxib (GKT137831) 400 mg + UDCA (N=38) Setanaxib (GKT137831) 400 mg + UDCA (N=36)	Placebo (N=37)
Schattenberg, et al^51^ (NCT03124108)	Phase 2 double-blind RCT	International, multi-center France, Germany, Spain, UK, US	Adult patients with PBC, with incomplete response to UDCA (defined as ALP >1.67×ULN). Patients had been treated with UDCA for ≥12 mo, and had been at a stable dose for ≥6 mo	Elafibranor 80 mg + UDCA (N=15) Elafibranor 120 mg + UDCA (N=14)	Placebo + UDCA (N=15)
Schramm 2022, et al^52^(NCT02516605)	Phase 2 double-blind RCT	International, multi-center Canada, Germany, Poland, Russia, UK, US	Patients aged ≥18 y with BMI of 18−40 kg/m^2^ and a confirmed diagnosis of PBC	Tropifexor 30 µg + UDCA (N=11) Tropifexor 60 µg + UDCA (N=9) Tropifexor 90 µg + UDCA (N=12) Tropifexor 150 µg + UDCA (N=8)	Placebo + UDCA (N=21)
Poupon, et al^53^	Double-blind RCT (Phase: NR)	Multi-center Country NR	Patients with persistent abnormalities in liver function tests, in particular serum ALP activity of >1.5×ULN, in patients with PBC who have been previously treated with UDCA (13−15 mg/kg/d) for ≥8 mo	Colchicine 1 mg + UDCA (N=37)	Placebo + UDCA (N=37)
Wolfhagen, et al^54^	Double-blind RCT (Phase: NR)	Multi-center The Netherlands	Patients with an established diagnosis of PBC, treated with UDCA (10 mg/kg/d) for ≥1 y, and none of them had achieved a complete remission of the disease	Prednisone 10 mg, azathioprine 50 mg + UDCA (N=26)	Placebo + UDCA (N=24)
Xiang, et al^55^ (NCT03345589)	Phase 4, open-label RCT	Single-center China	Patients aged 18−70 y with a diagnosis of PBC, treated with 13−15 mg/kg/d UDCA for ≥6 mo and with a suboptimal response to UDCA [defined using Paris 2 criteria (ALP or AST >1.5×ULN or an abnormal total bilirubin level)]	UDCA 18−22 mg (N=37)	UDCA 13−15 mg (N=36)
Studies of patients with inadequate response to UDCA, or who cannot tolerate UDCA (in which case UDCA is not received)					
Bowlus, et al^56^ (NCT02955602)	Phase 2 open-label RCT	International, multi-center Canada, Germany, UK, US	Patients aged 18−75 y, with a diagnosis of PBC and on a stable and recommended dose of UDCA for the past 12 mo, or intolerant to UDCA	Seladelpar 2 mg (N=11) Seladelpar 5 mg (N=53) Seladelpar 10 mg (N=55)	NR
COBALT (NCT02308111)	Phase 4	International, multi-center Argentina, Australia, Austria, Belgium, Brazil, Bulgaria, Canada, Chile, Denmark, Estonia, Finland, France, Germany, Hong Kong, Hungary, Israel, Italy, Republic of Korea, Lithuania, Mexico, Netherlands, New Zealand, Poland, Portugal, Serbia, Spain, Sweden, Switzerland, Turkey, UK, US	Patients ≥18 y with a definite or probable diagnosis of PBC; a mean total bilirubin >ULN and ≤5×ULN and/or a mean ALP >3×ULN; either not taking UDCA (no UDCA dose in the past 3 mo) or have been taking UDCA for at least 12 mo with a stable dose for ≥3 mo before day 0	OCA 5–10 mg (N=168)	Placebo (N=166)
ELATIVE (NCT04526665)	Phase 3 double-blind RCT	International, multi-center Argentina, Belgium, Brazil, Canada, Chile, France, Germany, Italy, South Africa, Spain, Switzerland, Turkey, UK, US	Patients aged 18−75 y with a definite or probable PBC diagnosis. Patients with ALP ≥1.67×ULN, total bilirubin ≤2×ULN; taking UDCA for ≥12 mo (stable dose ≥3 mo), or unable to tolerate UDCA treatment (no UDCA for ≥3 mo) before screening	Elafibranor 80 mg (N=108)	Placebo (N=53)
GLIMMER (NCT02966834)	Phase 2 double-blind RCT	International, multi-center Italy, Spain, UK, US, France, Australia, Poland, Japan, Canada, Germany	Patients aged 18−80 y with proven PBC. Patients concurrently taking UDCA were required to be on stable doses for >8 wk, and patients who had stopped taking UDCA because of intolerance could be enrolled 8 wk after their last dose of UDCA	Linerixibat 20 mg (N=16) Linerixibat 40 mg (N=23) Linerixibat 90 mg (N=23) Linerixibat 180 mg (N=27) Linerixibat 90 mg (N=22)	Placebo (N=36)
Hegade, et al^57^ (NCT01899703)	Phase 2 double-blind RCT	Multi-center UK	Patients 18−75 y with proven or likely PBC, and with ongoing pruritus. Patients were on stable doses of UDCA for >8 wk at the time of screening. Patients not taking UDCA due to intolerance were also eligible	Linerixibat (GSK2330672) 45 mg (twice per day; N=21)	Placebo (N=21)
ENHANCE (NCT03602560)	Phase 3 double-blind RCT	International, multi-center Argentina, Australia, Austria, Belgium, Canada, Chile, France, Germany, Greece, Hungary, Israel, Italy, Korea, Republic of, Mexico, Netherlands, New Zealand, Poland, Romania, Russian Federation, Serbia, Spain, UK, and US	Patients aged 18−75 y with a diagnosis of PBC. Patients were on a stable and recommended dose of UDCA for the past 12 mo or were intolerant to UDCA (last dose of UDCA >3 mo before screening)	Seladelpar 5−10 mg (N=89) Seladelpar 10 mg (N=89)	Placebo (N=87)
EPICS (NCT03112681)	Phase 2 double-blind RCT	Multi-center US	Patients aged 18−75 y, with a confirmed PBC diagnosis. Patients were on therapeutic doses of UDCA for ≥12 mo and stable therapy for ≥3 mo before enrollment. Patients who were unable to tolerate UDCA and did not receive UDCA for ≥3 mo from the date of screening were eligible	Saroglitazar 2 mg (N=14) Saroglitazar 4 mg (N=12)	Placebo (N=10)
NCT02943447	Phase 2 double-blind RCT	International, multi-center Austria, Canada, UK, US	Patients with definite or probable PBC with UDCA use at a stable dose for ≥12 mo or intolerant of UDCA with no UDCA use for ≥12 mo	Cilofexor 30 mg (N=30) Cilofexor 100 mg (N=28)	Placebo (N=13)
CLARITY (NCT01904058)	Phase 2 double-blind RCT	International, multi-center Canada, UK, US	Patients diagnosed with PBC and patients with moderate to severe pruritus. Patients were taking UDCA for at ≥6 mo or were unable to tolerate UDCA	LUM001 10 mg + UDCA (N=21) (Cohort A) LUM001 20 mg + UDCA (N=21) (Cohort B)	Placebo + UDCA (Cohort A N=11; Cohort B N=13)
NCT03394924	Phase 2 triple-blind RCT	International, multi-center Australia, Austria, Belgium, Canada, France, Germany, Netherlands, Spain, UK, US	Patients aged 18−75 y, with a diagnosis of PBC and on a stable dose of UDCA 12−20 mg/kg/d for ≥6 mo before screening, or intolerant of UDCA	EDP-305 1 mg (N=31) EDP-305 2.5 mg (N=28)	Placebo
NCT03633227	Phase 4 quadruple-blind RCT	International, multi-center Argentina, Australia, Belgium, Brazil, Canada, Estonia, Germany, Hungary, Italy, Lithuania, Spain, US	Patients with a definite or probable diagnosis of PBC with evidence of cirrhosis (biopsy consistent with PBC stage 4 liver stiffness, or clinical evidence of absence of acute liver failure consistent with cirrhosis); taking UDCA for ≥12 mo (stable dose for ≥3 mo) before day 1, or unable to tolerate or unresponsive to UDCA (no UDCA for ≥3 mo)	OCA 5−20 mg weekly (N=10)	Placebo (N=12)
POISE (NCT01473524)	Phase 3 double-blind RCT	International, multi-center Australia, Austria, Belgium, Canada, France, Germany, Italy, Netherlands, Poland, Spain, Sweden, UK, US	Patients ≥18 y who had received a definite or probable diagnosis of PBC, taking UDCA for ≥12 mo (stable dose for 3 mo), or unable to tolerate UDCA (no UDCA for 3 mo)	OCA 10 mg (N=73) OCA 5–10 mg (N=70)	Placebo (N=73)
RESPONSE (NCT04620733)	Phase 3, double-blind RCT	International, multi-center 24 countries (including EUR)	Patients aged 18–75 y with a diagnosis of PBC; received UDCA for the past 12 mo (stable dose >3 mo before screening) or intolerant to UDCA (last dose of UDCA >3 mo before screening)	Seladelpar 10 mg (N=128)	Placebo (N=65)
RDG-1/PBC	Phase 2, double-blind RCT	Multi-center NR	Patients with PBC and an inadequate response to UDCA, defined by serum ALP levels between 1.5×ULN and 10×ULN after at least 6 mo of UDCA therapy	RhuDex 50 mg (N=20) RhuDex 100 mg (N=21) RhuDex 200 mg (N=19)	Placebo (N=19)
Study 213	Phase 2, double-blind RCT	Setting NR	Adult patients with PBC who had ALP ≥ 1.5×ULN, had total bilirubin >ULN but <2×ULN, and took UDCA for at least 12 mo before day 1, or had not taken UDCA for 3 mo before day 1	Bezafibrate 200 mg IR + OCA (N=19) Bezafibrate 400 mg SR + OCA (N=18) Bezafibrate 200 mg IR + placebo (N=19) Bezafibrate 400 mg SR + placebo (N=19)	NR
Study 214	Phase 2, double-blind RCT	Setting NR	Patients with PBC who had an inadequate response to or were unable to tolerate UDCA	Bezafibrate 100 mg IR (N=NR) Bezafibrate 400 mg IR (N=NR) OCA 5 mg + bezafibrate 100 mg IR (N=NR) OCA 5 mg + bezafibrate 400 mg IR (N=NR)	NR
Tanaka 2025 (NCT03092765)^58^	Phase 2 double-blind RCT	Multi-center Japan	Patients aged ≥20 and <80 years old, with PBC who met at least 2 of the following: (a) elevation of cholestatic enzymes, (b) positivity for AMA, (c) histology compatible with PBC	E6011 10 mg/kg/EOW (n=14) E6011 15 mg/kg/mo (n=4) E6011 15 mg/kg/mo (n=4)	Placebo (n=7)
TRANSFORM (NCT05014672)	Phase 2 double-blind RCT	Multi-center US, Australia, Austria, Belgium, Canada, Czechia, France, Germany, Greece, Hungary, Israel, Italy, New Zealand, Poland, Spain, Sweden, Switzerland, UK	Patients aged ≥18 y with a definite or probable PBC diagnosis with 2 of the 3 criteria: ALP levels ≥1.67×ULN; documented history of positive AMA titer or positive PBC-specific antibodies; historical liver biopsy consistent with PBC	Setanaxib 1200 mg/d (n=24) Setanaxib 1600 mg/d (n=25)	Placebo (n=27)
Unspecified UDCA setting					
FITCH (NCT02701166)^a^	Double-blind RCT (Phase: NR)	International, multi-center Netherlands, Spain	Patients aged ≥18 y with a diagnosis of PBC, PSC, and SSC as defined by EASL 2009, suffering from itch without primary dermatological abnormalities, and with an intensity score of 5 or higher on a scale from 0 to 10, scored twice within the week before treatment	Bezafibrate 400 mg (N=11^b^) *Concomitant UDCA was permitted*	Placebo (N=15^b^) *Concomitant UDCA was permitted*
Silveira 2017^c^ ^59^	Double-blind RCT (Phase: NR)	Single-center US	Patients with PBC with fatigue for >6 mo, and had received UDCA (13–15 mg/kg/d) for ≥6 mo before enrollment into this study	Modafinil 100 mg (N=17)	Placebo (N=19)

^a^ Concomitant UDCA was permitted, but it was not reported whether patients had an inadequate response to prior UDCA, or how many patients were unable to continue UDCA.

^b^ Patients with PBC.

^c^ UDCA for at least 6 months was required for enrollment in the trial, but it is unclear if patients continued on UDCA throughout the trial, and/or whether these patients had an inadequate response or intolerance to UDCA.

Abbreviations: AASLD, American Association for the Study of Liver Diseases; ALP, alkaline phosphatase; ALT, alanine transaminase; AMA, anti-mitochondrial antibody; AST, aspartate aminotransferase; BID, twice a day; BMI, body mass index; CVD, cardiovascular disease; EASL, European Association for the Study of the Liver; EOW, every other week; GGT, gamma-glutamyl transferase; IR, immediate release; MTX, methotrexate; NR, not reported; OCA, obeticholic acid; PBC, primary biliary cholangitis; PSC, primary sclerosing cholangitis; QD, once daily; RCT, randomized controlled trial; SLR, systematic literature review; SR, sustained release; SSC, secondary sclerosing cholangitis; UDCA, ursodeoxycholic acid; UK, United Kingdom; ULN, upper limit of normal; US, United States.

The most frequently reported interventions were bezafibrate (n=9), OCA (n=4), and seladelpar (n=4). Colchicine, elafibranor, fenofibrate, linerixibat, and methotrexate were all reported twice, and other drugs, including budesonide, saroglitazar, and setanaxib, were reported in one study each (Table 2). Studies had variable sample sizes ranging from 10 to 334 patients.

### Patient population

Across the 44 studies, 40 reported sex, 17 reported race, and all reported age. Most patients were female (range: 60%–100%), predominantly White (range: 63%–100%), and the mean age range was 45.0–64.0 years (Supplemental Table S11, https://links.lww.com/HC9/C483).

### Baseline disease characteristics

Disease staging at baseline was reported in 13 studies (29.5%; Supplemental Table S11, https://links.lww.com/HC9/C483), with several different scales used. The Ludwig scale and Scheuer scale were reported in 3 and 6 studies, respectively.^41^,^43^,^45^,^46^,^50^,^53^,^54^,^59^,^60^ The Nakamura scale was reported in 1 study,^53^,^58^and the method of disease staging was not reported in 3 studies.^38^,^42^,^48^ Disease duration before study entry was not consistently reported [16 studies (36.4%)], with a mean disease duration range of 2.7–12.8 years.^53^,^58^ Serum levels of biochemical markers were measured at baseline for ALP [n=38 studies (86.4%)], TB [n=33 (75%)], GGT [n=22 (50%)], and albumin [n=19 (48.2%)].^61^ Baseline values of measures of pruritus and fatigue were not as frequently reported [n=14 (34.1%), n=4 (9.1%), respectively].

### Treatment history

Treatment history was reported in the majority of studies [32 studies (72.7%)], where concomitant UDCA treatment was reported in 31 studies (70.5%), with 81%–100% of patients receiving UDCA during the clinical trial period. Baseline daily UDCA dosage was reported in 20 studies (45.5%) with the mean dose ranging from 10.0 mg/kg to 17.3 mg/kg (Supplemental Table S12, https://links.lww.com/HC9/C483). Previous treatment with OCA and fibrates was reported in 3 (6.8%) and 2 studies (4.5%), respectively.

### Biochemical response

Composite biochemical response endpoints were reported in 15 studies (34.1%; Table 3). Of these, 12 studies (27.3%) specified the criteria used to define response (Table 3), where 5 studies (18.2%) used multiple criteria.

**TABLE 3:** Biochemical response to treatment

Study	Response denominator	Response	Response outcome definitions
Askari, et al^37^	NA	NR	NA
BEZURSO^36^ (NCT01654731)	Bezafibrate + UDCA: 50 placebo + UDCA: 50	*All responses measured at month 24* *Normal serum levels of ALP, AST, total bilirubin, and albumin with a normal prothrombin index:* Bezafibrate + UDCA: 31% Placebo + UDCA: 0% *Paris I:* Bezafibrate + UDCA: 39 (85%) Placebo + UDCA: 23 (55%) *Paris II* *:* Bezafibrate + UDCA: 32 (70%) Placebo + UDCA: 4 (10%) *Barcelona:* Bezafibrate + UDCA: 31 (67%) Placebo + UDCA: 4 (10%) *Toronto:* Bezafibrate + UDCA: 41 (89%) Placebo + UDCA: 9 (21%) *Rotterdam:* Bezafibrate + UDCA: 40 (89%) Placebo + UDCA: 31 (78%) *Globe score:* Bezafibrate + UDCA: 37 (83%) Placebo + UDCA: 23 (61%)	• Normal serum levels of ALP, AST, ALT, total bilirubin, and albumin, with a normal prothrombin index • Paris I, Paris II, Barcelona, Toronto, Rotterdam criteria
Bowlus, et al^56^ (NCT02955602)	**POISE criteria:** *Week 12:* Seladelpar 2 mg: 11 Seladelpar 5 mg: 47 Seladelpar 10 mg: 51 *Week 52:* Seladelpar 2 mg: 11 Seladelpar 5 mg: 45 Seladelpar 10 mg: 49	**POISE criteria:** *Week 12* : Seladelpar 2 mg: 5 (46%) Seladelpar 5 mg: 23 (49%) Seladelpar 10 mg: 34 (67%) *Week 52:* Seladelpar 2 mg: 7 (64%) Seladelpar 5 mg: 24 (53%) Seladelpar 10 mg: 33 (67%)	• POISE criteria
COBALT (NCT02308111)	NA	NR	NA
Combes, et al^37^	NA	NR	NA
ELATIVE (NCT04526665)	**All criteria:** *Week 4–Week 52:* Placebo: 53 Elafibranor 80 mg: 108 *Week 78, POISE criteria:* Placebo: 13 Elafibranor 80 mg: 27	**POISE criteria:** *Week 4:* Placebo: 0% Elafibranor 80 mg: 41% *Week 13* : Placebo: 4% Elafibranor 80 mg: 59% *Week 26:* Placebo: 0% Elafibranor 80 mg: 55% *Week 39:* Placebo: 2% Elafibranor 80 mg: 57% *Week 52:* Placebo: 2 (4%) Elafibranor 80 mg: 55 (51%) *Week 78:* Placebo: 0 (0%) Elafibranor 80 mg: 19 (70%) **Paris I:** *Week 52* : Placebo: 47% Elafibranor 80 mg: 69% **Paris II:** *Week 52:* Placebo: 6% Elafibranor 80 mg: 43% **Barcelona:** *Week 52:* Placebo: 0% Elafibranor 80 mg: 55% **Momah/Lindor:** *Week 52:* Placebo: 9% Elafibranor 80 mg: 52% **Complete biochemical response:** *Week 52:* Placebo: 0% Elafibranor 80 mg: 9%	• POISE criteria • Paris I, Paris II, Barcelona, Momah/Lindor criteria • Complete biochemical response: normal ALP, total bilirubin, AST, ALT, albumin, and international normalized ratio
ENHANCE (NCT03602560)	**POISE criteria:** *Month 1:* Placebo: 78 Seldapar 5 mg: 80 Seldapar 10 mg: 79 *Month 3:* Placebo: 56 Seldapar 5 mg: 56 Seldapar 10 mg: 55 *Month 6:* Placebo: 23 Seldapar 5 mg: 26 Seldapar 10 mg: 20	**POISE criteria:** *Month 1:* Placebo: 8 (10.3%) Seldapar 5 mg: 38 (47.5%) Seldapar 10 mg: 51 (64.6%) *Month 3:* Placebo: 7 (12.5%) Seldapar 5 mg: 32 (57.1%) Seldapar 10 mg: 43 (78.2%) *Month 6:* Placebo: 5 (21.7%) Seldapar 5 mg: 16 (61.5%) Seldapar 10 mg: 14 (70.0%)	• POISE criteria
EPICS (NCT03112681)	**POISE criteria:** *All timepoints:* Placebo: 10 Saroglitazar 2 mg: 14 Saroglitazar 4 mg: 13	**POISE criteria:** *Week 4:* Placebo: 0% Saroglitazar 2 mg: 64% Saroglitazar 4 mg: 77% *Week 8:* Placebo: 0% Saroglitazar 2 mg: 69% Saroglitazar 4 mg: 73% *Week 12:* Placebo: 0% Saroglitazar 2 mg: 77% Saroglitazar 4 mg: 70% *Week 16:* Placebo: 10% Saroglitazar 2 mg: 71% Saroglitazar 4 mg: 69%	• POISE criteria
FITCH (NCT02701166)	NA	NR	NA
GLIMMER (NCT02966834)	NA	NR	NA
Gonzalez-Koch^38^	NA	NR	NA
HAART (NCT01614405)	NA	NR	NA
Hegade, et al^57^ (NCT01899703)	NA	NR	NA
Hirschfield, et al^39^ (NCT00550862)	**All criteria:** *All timepoints:* Placebo + UDCA: 38 OCA 10 mg + UDCA: 38 OCA 25 mg + UDCA: 48 OCA 50 mg + UDCA: 41	**Paris I:** *Baseline:* Placebo + UDCA: 5 OCA 10 mg + UDCA: 11 OCA 25 mg + UDCA: 10 OCA 50 mg + UDCA: 10 *Day 85 (biochemical response):* Placebo + UDCA: 5 OCA 10 mg + UDCA: 7 OCA 25 mg + UDCA: 3 OCA 50 mg + UDCA: 5 Placebo + UDCA: 0% OCA 10 mg + UDCA: 36% OCA 25 mg + UDCA: 70% OCA 50 mg + UDCA: 50% **Paris II:** *Baseline: * Placebo + UDCA: 34 OCA 10 mg + UDCA: 34 OCA 25 mg + UDCA: 46 OCA 50 mg + UDCA: 40 *Day 85 (biochemical response):* Placebo + UDCA: 30 OCA 10 mg + UDCA: 22 OCA 25 mg + UDCA: 30 OCA 50 mg + UDCA: 25 *Day 85 (baseline non-responders with treatment effect):* Placebo + UDCA: 12% OCA 10 mg + UDCA: 35% OCA 25 mg + UDCA: 35% OCA 50 mg + UDCA: 35% **Toronto I** *Baseline:* Placebo + UDCA: 32 OCA 10 mg + UDCA: 30 OCA 25 mg + UDCA: 39 OCA 50 mg + UDCA: 35 *Day 85 (biochemical response):* Placebo + UDCA: 28 OCA 10 mg + UDCA: 17 OCA 25 mg + UDCA: 22 OCA 50 mg + UDCA: 19 *Day 85 (baseline non-responders with treatment effect)* : Placebo + UDCA: 13% OCA 10 mg + UDCA: 43% OCA 25 mg + UDCA: 44% OCA 50 mg + UDCA: 43% **Toronto II:** *Baseline:* Placebo + UDCA: 29 OCA 10 mg + UDCA: 28 OCA 25 mg + UDCA: 38 OCA 50 mg + UDCA: 30 *Day 85 (biochemical non-response):* Placebo + UDCA: 24 OCA 10 mg + UDCA: 17 OCA 25 mg + UDCA: 20 OCA 50 mg + UDCA: 14 *Day 85 (baseline non-responders with treatment effect):* Placebo + UDCA: 17% OCA 10 mg + UDCA: 39% OCA 25 mg + UDCA: 47% OCA 50 mg + UDCA: 50%	• Paris I and II, Toronto I and II criteria
Hirschfield, et al^40^ (NCT00746486)	NA	NR	NA
Hosonuma, et al^41^	NA	NR	NA
Ikeda, et al^42^	NA	NR	NA
Iwasaki, et al^43^	NA	NR	NA
Jones, et al^44^ (NCT02609048)	NA	NR	NA
Kanda, et al^45^	NA	NR	NA
Kurihara, et al^46^	NA	NR	NA
Li 2022 (ChiCTR1800020160)	**All criteria:** *All timepoints* : UDCA alone: 24 Fenofibrate + UDCA: 24	**Normalization of ALP, GGT and total bilirubin:** *Month 0* *:* UDCA alone: 0% Fenofibrate + UDCA: 0% *Month 3:* UDCA alone: 0% Fenofibrate + UDCA: 12.5% *Month 6:* UDCA alone: 0% Fenofibrate + UDCA: 16.7% *Month 12:* UDCA alone: 0% Fenofibrate + UDCA: 20.8% **Barcelona** *Month 0:* UDCA alone: 8.3% Fenofibrate + UDCA: 16.7% *Month 3:* UDCA alone: 0% Fenofibrate + UDCA: 50% *Month 6:* UDCA alone: 4.2% Fenofibrate + UDCA: 45.8% *Month 12:* UDCA alone: 4.2% Fenofibrate + UDCA: 54.2% **Paris I:** *Month 0:* UDCA alone: 62.5% Fenofibrate + UDCA: 54.2% *Month 3:* UDCA alone: 58.3% Fenofibrate + UDCA: 54.2% *Month 6:* UDCA alone: 58.3% Fenofibrate + UDCA: 66.7% *Month 12:* UDCA alone: 50% Fenofibrate + UDCA: 62.5% **Paris II:** *Month 0* : UDCA alone: 12.5% Fenofibrate + UDCA: 25% *Month 3* : UDCA alone: 12.5% Fenofibrate + UDCA: 41.7% *Month 6:* UDCA alone: 8.3% Fenofibrate + UDCA: 50% *Month 12:* UDCA alone: 8.3% Fenofibrate + UDCA: 54.2% **Rochester** *Month 0:* UDCA alone: 33.3% Fenofibrate + UDCA: 54.2% *Month 3:* UDCA alone: 41.7% Fenofibrate + UDCA: 87.5% *Month 6:* UDCA alone: 41.7% Fenofibrate + UDCA: 87.5% *Month 12:* UDCA alone: 29.2% Fenofibrate + UDCA: 91.7% **Toronto** *Month 0:* UDCA alone: 25% Fenofibrate + UDCA: 45.8% *Month 3:* UDCA alone: 20.8% Fenofibrate + UDCA: 87.5% *Month 6:* UDCA alone: 25% Fenofibrate + UDCA: 87.5% *Month 12:* UDCA alone: 16.7% Fenofibrate + UDCA: 75%	• Normalization of ALP, GGT, and total bilirubin • Barcelona, Paris I, Paris II, Rochester, Toronto criteria
Liberopoulos^47^	NA	NR	NA
Lim, et al^48^	NA	NR	NA
Mayo^49^ (NCT02026401)	NA	NR	NA
Nakai, et al^50^	NA	NR	NA
CLARITY (NCT01904058)	NA	NR	NA
NCT02943447	NA	NR	NA
NCT03226067	NA	NR	NA
NCT03394924	NA	NR	NA
NCT03633227	NA	NR	NA
POISE (NCT01473524)	**POISE criteria:** *All timepoints:* Placebo: 73 OCA 5–10 mg: 70 OCA 10 mg: 73 **GGT <3.2×ULN and ALP <1.5×ULN:** *Month 3:* All OLE patients: 118 *Month 6:* Placebo: NR *Month 9:* Placebo: NR OCA 5–10 mg: 66 OCA 10 mg: 68 *Month 12:* OCA 5–10 mg: 66 OCA 10 mg: 68 *Month 51:* All OLE patients: 91 *Month 60:* All OLE patients: 46	**POISE criteria:** *Month 6* : Placebo: 7% OCA 5–10 mg: 34% OCA 10 mg: 51% *Month 12:* Placebo: 10% OCA 5–10 mg: 46% OCA 10 mg: 47% **GGT <3.2×ULN and ALP <1.5×ULN:** *Month 3* : All OLE patients: 19 (16%) *Month 6:* Placebo: 1 (NR) *Month 9:* Placebo: 1 (NR) OCA 5–10 mg: 11 (17%) OCA 10 mg: 21 (31%) *Month 12:* OCA 5–10 mg: 11 (17%) OCA 10 mg: 18 (26%) *Month 51:* All OLE patients: 35 (38%) *Month 60:* All OLE patients: 17 (37%)	• POISE criteria • ALP ≤1.5 ULN, AST ≤1.5 ULN and total bilirubin ≤ULN • Total bilirubin ≤ULN and albumin ≥LLN • ALP ≤1.67×ULN and total bilirubin ≤ULN • ALP ≤3×ULN, AST ≤2×ULN and total bilirubin ≤ULN • GGT <3.2×ULN and ALP <1.5×ULN
Poupon, et al^53^	NA	NR	NA
RDG-1/PBC	NA	NR	NA
RESPONSE (NCT04620733)	**POISE criteria:** *All timepoints:* Placebo: 65 Seladelpar 10 mg: 128	**POISE criteria:** *Month 1:* Placebo: 5 (7.7%) Seladelpar 10 mg: 76 (59.4%) *Month 3:* Placebo: 7 (10.8%) Seladelpar 10 mg: 79 (61.7%) *Month 6:* Placebo: 12 (18.5%) Seladelpar 10 mg: 85 (66.4%) *Month 9:* Placebo: 12 (18.5%) Seladelpar 10 mg: 79 (61.7%) *Month 12:* Placebo: 13 (20.0%) Seladelpar 10 mg: 79 (61.7%)	• POISE criteria
Schattenberg^51^ (NCT03124108)	**POISE criteria:** *All timepoints:* Placebo + UDCA: 15 Elafibranor 80 mg + UDCA: 15 Elafibranor 120 mg + UDCA: 14	**POISE criteria:** *Week 12:* Placebo + UDCA: 1 (6.7%) Elafibranor 80 mg + UDCA: 10 (66.7%) Elafibranor 120 mg + UDCA: 11 (78.6%) **Paris I:** Placebo + UDCA: 53.3% Elafibranor 80 mg + UDCA: 80% Elafibranor 120 mg + UDCA: 78.6% **Paris II:** Placebo + UDCA: 0% Elafibranor 80 mg + UDCA: 53.3% Elafibranor 120 mg + UDCA: 50% **Toronto I:** Placebo + UDCA: 6.7% Elafibranor 80 mg + UDCA: 66.7% Elafibranor 120 mg + UDCA: 78.6% **Toronto II:** Placebo + UDCA: 6.7% Elafibranor 80 mg + UDCA: 66.7% Elafibranor 120 mg + UDCA: 78.6% **ALP <1.5 ULN, total bilirubin <ULN, ALP reduction >40%:** Placebo + UDCA: 0% Elafibranor 80 mg + UDCA: 53.3% Elafibranor 120 mg + UDCA: 35.7%	• POISE criteria • Paris I, Paris II, Toronto I, Toronto II criteria • ALP <1.5 ULN, total bilirubin <ULN, ALP reduction >40%
Schramm, et al^52^ \(NCT02516605)	NA	NR	NA
Silveira, et al^59^	**50% or greater improvement in fatigue severity (quantified by the FFIS) after 12 wk of treatment:** *Week 12:* Placebo: 19 Modafinil: 17	**50% or greater improvement in fatigue severity (quantified by the FFIS) after 12 wk of treatment:** *Week 12:* Placebo: 2 (12.5%) Modafinil:3 (17.6%)	• 50% or greater improvement in fatigue severity (quantified by the FFIS) after 12 wk of treatment
Study 213	**All criteria:** *All timepoints:* Bezafibrate 200 mg + placebo: 19 Bezafibrate 200 mg + OCA: 19 Bezafibrate 400 mg + placebo: 19 Bezafibrate 400 mg + OCA: 18	**POISE criteria:** *Week 12:* Bezafibrate 200 mg + placebo: 84.2% Bezafibrate 200 mg + OCA: 78.9% Bezafibrate 400 mg + placebo: 89.5% Bezafibrate 400 mg + OCA: 83.3% **ALP, GGT, ALT and AST levels ≤ULN, with total bilirubin ≤0.6×ULN:** *Week 12:* Bezafibrate 200 mg immediate release:15.8% Bezafibrate 400 mg slow release: 31.6% OCA 5–10 mg + bezafibrate 200 mg immediate release: 31.6% OCA 5–10 mg + bezafibrate 400 mg sustained release: 44.4% **Rate of biochemical remission:** *Month 6:* Bezafibrate 200 mg + placebo: 31.6% Bezafibrate 200 mg + OCA: 47.4% Bezafibrate 400 mg + placebo: 42.1% Bezafibrate 400 mg + OCA: 66.7%	• POISE criteria • Normalization of ALP, ALT, AST, GGT, and total bilirubin levels • Rate of biochemical remission
Study 214	**ALP, GGT, ALT, and AST levels ≤ULN, with total bilirubin ≤0.6×ULN** *Week 12:* Bezafibrate 100 mg immediate release: 11 Bezafibrate 400 mg immediate release: 9 OCA 5 mg + bezafibrate 100 mg immediate release: 11 OCA 5 mg + bezafibrate 400 mg immediate release: 10	**ALP, GGT, ALT and AST levels ≤ULN, with total bilirubin ≤0.6×ULN** *Week 12:* Bezafibrate 100 mg immediate release: 9.1% Bezafibrate 400 mg immediate release: 18.2% OCA 5 mg + bezafibrate 100 mg immediate release: 11.1% OCA 5 mg + bezafibrate 400 mg immediate release: 40.0%	• ALP, GGT, ALT and AST levels ≤ULN, with total bilirubin ≤0.6×ULN
Tanaka et al^58^(NCT03092765)	NA	NR	NA
TRANFORM (NCT05014672)	**POISE** *Week 24:* Setanaxib 1200 mg: 24 Setanaxib 1600 mg: 25 Placebo: 27 **ALP <1.67×ULN and Total Bilirubin ≤1×ULN and ALP reduction ≥30%** *Week 24:* Setanaxib 1200 mg: 24 Setanaxib 1600 mg: 25 Placebo: 27 **ALP <1.67×ULN and Total Bilirubin ≤1×ULN and ALP reduction ≥40%** *Week 24:* Setanaxib 1200 mg: 24 Setanaxib 1600 mg: 25 Placebo: 27 **ALP <1.67×ULN and Total Bilirubin ≤1×ULN and ALP reduction ≥70%** *Week 24:* Setanaxib 1200 mg: 24 Setanaxib 1600 mg: 25 Placebo: 27 **ALP <1×ULN and total bilirubin ≤1×ULN** *Week 24:* Setanaxib 1200 mg: 24 Setanaxib 1600 mg: 25 Placebo: 27 **ALP reduction to <1.5×ULN and total bilirubin ≤1×ULN and ALP reduction ≥40%** *Week 24:* Setanaxib 1200 mg: 24 Setanaxib 1600 mg: 25 Placebo: 27	**POISE criteria** *Week 24:* Setanaxib 1200 mg: 1 Setanaxib 1600 mg: 4 Placebo: 1 **ALP <1.67×ULN and Total Bilirubin ≤1×ULN and ≥30% ALP** *Week 24:* Setanaxib 1200 mg: 1 Setanaxib 1600 mg: 2 Placebo: 0 **ALP <1.67×ULN and Total Bilirubin ≤1×ULN and ≥40% ALP** *Week 24:* Setanaxib 1200 mg: 0 Setanaxib 1600 mg: 2 Placebo: 0 **ALP <1.67×ULN and Total Bilirubin ≤1×ULN and ≥70% ALP** *Week 24:* Setanaxib 1200 mg: 0 Setanaxib 1600 mg: 0 Placebo: 0 **ALP <1×ULN and total bilirubin ≤1×ULN** *Week 24:* Setanaxib 1200 mg: 0 Setanaxib 1600 mg: 1 Placebo: 0 **ALP reduction to <1.5×ULN and total bilirubin ≤1×ULN and a ≥40% ALP reduction** *Week 24* : Setanaxib 1200 mg: 0 Setanaxib 1600 mg: 2 Placebo: 0	• POISE criteria • ALP <1.67×ULN and Total Bilirubin ≤1×ULN and ALP reduction ≥30% • ALP <1.67×ULN and Total Bilirubin ≤1×ULN and ALP reduction ≥40% • ALP <1.67×ULN and Total Bilirubin ≤1×ULN and ALP reduction ≥70% • ALP <1×ULN and total bilirubin ≤1×ULN • ALP reduction to <1.5×ULN and total bilirubin ≤1×ULN and ALP reduction ≥40%
Wolfhagen et al^54^	NA	NR	NA
Xiang et al^55^ (NCT03345589)	**Both criteria:** *Both timepoints:* UDCA 13–15 mg: 36 UDCA 18–22 mg: 37	**Barcelona:** *Month 6:* UDCA 13–15 mg: 9 (25%) UDCA 18–22 mg: 12 (32.4%) *Month 12:* UDCA 13–15 mg: 14 (38.9%) UDCA 18–22 mg: 18 (48.6%) **Paris II:** *Month 6:* UDCA 13–15 mg: 13 (36.1%) UDCA 18–22 mg: 22 (59.4%) *Month 12:* UDCA 13–15 mg: 17 (47.2%) UDCA 18–22 mg: 22 (59.4%)	• Barcelona, Paris II criteria

Abbreviations: NA, not applicable; NR, not reported; OCA, obeticholic acid; SD, standard deviation; SE, standard error; UDCA, ursodeoxycholic acid.

Upper limit of normal (ULN) values for biochemical markers that were used in definitions of response were reported in 5 out of 6 phase III studies and varied between studies. For example, the ULN value for ALP was defined as 104 U/L for women and 129 U/L for men in Schattenberg 2021 (elafibranor), ELATIVE (elafibranor), and Hirschfield 2021 (budesonide),^15^,^40^,^51^,^62^ 150 U/L in ENHANCE (seladelpar),^63^ and 116 U/L in RESPONSE (seladelpar).^26^ The ULN values for other biochemical markers, such as TB and ALT, also differed across studies (Supplemental Table S13, https://links.lww.com/HC9/C483).

POISE criteria, defined as ALP <1.67×ULN, with a reduction of ≤15% from baseline, and TB ≤ULN, were the most commonly used criteria, reported in 8 studies (18.2%), all of which were phase II (5 studies) or phase III (3 studies). Paris I criteria (defined as ALP <3.0×ULN, AST <2.0×ULN, and bilirubin <1 mg/dL) were reported in 5 studies (11.4%), and Paris II criteria (defined as ALP <1.5×ULN, AST <1.5×ULN, and bilirubin <1 mg/dL) in 6 studies (13.6%; Table 3). Barcelona criteria (defined as ALP normalization or >40% reduction) were reported in 4 studies (9.1%; Table 3). Toronto criteria (defined as ALP ≤1.67×ULN by 2 years of treatment), Toronto I criteria (defined as ALP <1.67×ULN), and Toronto II criteria (ALP <1.76×ULN) were reported in 2 studies (4.5%) each (Table 3). Other criteria, including complete biochemical response, modified versions of Toronto I, combinations of normalization of ALT, AST, GGT, or TB levels, and biochemical remission, were also reported.

While all placebo-controlled studies demonstrated a higher percentage of responders in treatment groups compared with placebo groups, notably, rates of response in placebo groups were highly variable between studies (Table 3).

### Biochemical measures

ALP, TB, GGT, and albumin were the most commonly reported biochemical markers. Other measures included AST, ALT, indirect and conjugated bilirubin, serum tumor necrosis factor-alpha (TNF-α), platelet counts, triglycerides, bile acid composition, IgA, IgG, and IgM, and changes in prothrombin.

#### Alkaline phosphatase

ALP was reported in 39 studies (88.6%; Table 4), of which 10 (22.7%) reported absolute baseline and end-of-study values. Changes from baseline in ALP, expressed as absolute and/or relative values, were reported across most treatments; changes were reported as mean values in 20 studies (43.2%) and as median values in 5 studies (11.4%; Table 4).

**TABLE 4:** Change from baseline in alkaline phosphatase (ALP) and total bilirubin (TB)

Study name	Timepoint	Treatment arm	Denominator	ALP				TB			
				Unit	Baseline value, mean unless otherwise specified	Value at timepoint, mean unless otherwise specified	Change from baseline	Unit	Baseline value, mean unless otherwise specified	Value at timepoint, mean unless otherwise specified	Change from baseline
Askari, et al^35^	NR	Tetrathiomolybdate + UDCA	13	IU/L	281 (SE=31.9)	277 (SE=32.4)	NR	NR	0.9 (SE=0.1)	NR	NR
		Placebo + UDCA	15		257 (SE=32.6)	244 (SE=33.1**)**	NR	NR	1.1 (SE=0.2)	NR	NR
BEZURSO^36^ (NCT01654731)	Month 24	Bezafibrate + UDCA	50	IU/L, %	NR	NR	Median % CfB: −60%	%	NR	NR	Median % CfB: −14%
		Placebo + UDCA	50		NR	NR	Median % CfB: 0%		NR	NR	Median % CfB: 18%
Bowlus, et al^56^ (NCT02955602)	Week 52	Seladelpar 2 mg	10	U/L	NR	NR	Mean CfB: −101.20 (SE=107.956)	mg/dL, %	0.6 (SE=0.04)	0.6 (SE=0.05)	Mean CfB: 0.002 (SD=0.152, SE=0.05); Mean % CfB: 2.3% (SE=7.7)
		Seladelpar 5 mg	42		NR	NR	Mean CfB: −158.3 (SE=143.668)		0.8 (SE=0.05)	0.7 (SE=0.05)	Mean CfB: −0.03 (SD=0.263, SE=0.04); Mean % CfB: −0.4% (SE=4.2)
		Seladelpar 10 mg	48		NR	NR	Mean CfB: −133.8 (SE=76.151)		0.8 (SE=0.05)	0.7 (SE=0.05)	Mean CfB: −0.07 (SD=0.068, SE=0.03); Mean % CfB: −7% (SE=3.2)
		Seladelpar 2 mg	10	U/L, %	300.4 (SE=36.6)	206.9 (SE=41.9)	Mean % CfB: −32.7% (SE=6.8)	NR	NR	NR	NR
		Seladelpar 5 mg	42		353.3 (SE=27.5)	206 (SE=19.4)	Mean % CfB: −40.1% (SE=3.6)	NR	NR	NR	NR
		Seladelpar 10 mg	48		301 (SE=19.1)	166.7 (SE=13.2)	Mean % CfB: −44.2% (SE=2.2)	NR	NR	NR	NR
COBALT (NCT02308111)	Month 12	OCA 5 mg titrated to 10 mg	126	U/L	NR	NR	LS Mean CfB: −144.8 (SE=12.05)	NR	NR	NR	LS Mean CfB: 0.26 (SE=0.113)
		Placebo	138		NR	NR	LS Mean CfB: −68.8 (SE=11.72)	NR	NR	NR	LS Mean CfB: 0.29 (SE=0.111)
	Month 24	OCA 5 mg titrated to 10 mg	98		NR	NR	LS Mean CfB: −156.4 (SE=14.93)	NR	NR	NR	LS Mean CfB: 0.3 (SE=0.141)
		Placebo	102		NR	NR	LS Mean CfB: −113.1 (SE=14.60)	NR	NR	NR	LS Mean CfB: 0.63 (SE=0.138)
ELATIVE (NCT04526665)	Week 52	Placebo	47	U/L, %	NR	NR	Mean CfB: −8.8 (SD=91.2) Mean % CfB: 2% LS Mean CfB: −5.3	μmol/L	NR	NR	LS Mean CfB: 1.1
								mg/dL	NR	NR	Mean CfB: 0.05 (SD=0.25)
		Elafibranor 80 mg	94		NR	NR	Mean CfB: −117.7 (SD=96.5); Mean % CfB: −39%; LS Mean CfB: −117.0	μmol/L	−0.67 (n=93)	NR	LS Mean CfB: −0.1 (n=93)
								mg/dL	NR	NR	Mean CfB: 0.05 (SD=0.25)
		Placebo	53	U/L	321.3 (SD=121.9)	NR	NR	μmol/L	9.4 (SD=5.0)	NR	NR
		Elafibranor 80 mg	108		323.1 (SD=198.6)	NR	NR		9.7 (SD=5.1)	NR	NR
ENHANCE (NCT03602560)	Month 3	Seladelpar 5 mg	56	U/L, %	290.5 (SD=104.2)	NR	LS Mean CfB: −35.7	mg/dL, %	0.76 (SD=0.35)	NR	Mean % CfB: 0.76 (SD=0.35); LS Mean CfB: −6.1
		Seldapar 10 mg	55		290.8 (SD=109.1)	NR	Mean % CfB: 45 LS Mean CfB: −44.2		0.72 (SD=0.32)	NR	Mean % CfB: 0.72 (SD=0.32); LS Mean CfB: −4.0
		Placebo	56		293.4 (SD=106.2)	NR	LS Mean CfB: −3.7		0.71 (SD=0.32)	NR	Mean % CfB: 0.71 (SD=0.32); LS Mean CfB: 0.9
EPICS (NCT03112681)	Week 16	Saroglitazar 4 mg	13	U/L	323.2 (SD=111)	NR	Mean CfB: −162.31 (SD=113.98)	mg/dL	0.7 (SD=0.2)	NR	NR
		Saroglitazar 2 mg	14		351.3 (SD=161.3)	NR	Mean CfB: −163.82 (SD=111.84)		0.6 (SD=0.2)	NR	NR
		Placebo	10		294.9 (SD=73.3)	NR	Mean CfB: −11.15 (SD=27.36)		0.7 (SD=0.2)	NR	NR
Gonzalez-Koch, et al^38^	Week 48	Methotrexate + UDCA	13	U/L	1062 (SD=608)	NR	Mean CfB: −655	mg/dL	1.58 (SD=1.25)	NR	−0.5
		Placebo + UDCA	12		803 (SD=491)	NR	Mean CfB: −405	mg/dL	1.44 (SD=0.79)	NR	−0.55
HAART (NCT01614405)	Month 6	TDC/FTC/LPRr + UDCA	5	U/L, %	Median: 546	Median: 410	Median % CfB: −136	μmol/L	13	21	8
		Placebo + UDCA	6		Median: 355	Median: 315	Median % CfB: −40		29	17	−12
		TDC/FTC/LPRr + UDCA	5	×ULN, %	Median: 4.2	Median: 3.15	Median % CfB: −1.05	×ULN	0.65	1.05	0.4
		Placebo + UDCA	6		Median: 2.73	Median: 2.42	Median % CfB: −0.31		1.45	0.85	−0.6
Hegade, et al^38^ (NCT01899703)	Day 14	Post GSK2330672 sequence 1	11	IU/L	264 (SD=174.1)	229.4 (SD=114.5)	NR	μmol/L	12.2 (SD=5.49)	10.9 (SD=4)	NR
		Post GSK2330672 placebo 1	11		264 (SD=174.1)	247.1 (SD=126.3)	NR		12.2 (SD=5.49)	12.6 (SD=6.1)	NR
		Post GSK2330672 sequence 2	10		264 (SD=174.1)	291.8 (SD=202.4)	NR		12.2 (SD=5.49)	15.7 (SD=8.8)	NR
		Post GSK2330672 placebo 2	10		264 (SD=174.1)	318.1 (SD=252.3)	NR		12.2 (SD=5.49)	14.4 (SD=9.54)	NR
Hirschfield, et al^39^ (NCT00550862)	Day 85	Placebo + UDCA	38	%	NR	NR	Mean % CfB: −2.6% (SD=12.5)	mg/dL	Median: 0.15	Median: 0.19	NR
		OCA 10 mg + UDCA	38		NR	NR	Mean % CfB: −23.7% (SD=17.8)	mg/dL	Median: 0.2	Median: 0.19	NR
		OCA 25 mg + UDCA	48	%	NR	NR	Mean % CfB: −24.7% (SD=17.9)	mg/dL	Median: 0.2	Median: 0.12	NR
		OCA 50 mg + UDCA	41		NR	NR	Mean % CfB: −21% (SD=27.6)	mg/dL	Median: 0.25	Median: 0.2	NR
Hirschfield, et al^40^	Month 36	Budesonide + UDCA	40	U/L	327 (SD=227)	NR	Mean CfB: −95 (SD=166)	mg/dL	0.75 (SD=0.46)	NR	Mean CfB: −0.02 (SD=0.44)
		Placebo + UDCA	22		367 (SD=289)	NR	Mean CfB: −9 (SD=177)		0.67 (SD=0.41)	NR	Mean CfB: 0.59 (SD=2.22)
Hosonuma, et al^42^	Year 8	UDCA	14	IU/L	Median: 454	461	NR	NR	NR	NR	NR
		Bezafibrate + UDCA	13		Median: 423	290	NR	NR	NR	NR	NR
Ikeda, et al^42^	Year 2	Colchicine + UDCA	5	NR	NR	NR	Mean CfB: 0.76 (SD=0.05)	NR	NR	NR	Mean CfB: 0.81 (SE=0.04)
		UDCA	4	NR	NR	NR	Mean CfB: 1.26 (SD=0.19)	NR	NR	NR	Mean CfB: 0.85 (SE=0.14)
Iwasaki, et al^43^	Week 52	Bezafibrate + UDCA	NR	IU/L	643 (SD=262)	NR	NR	mg/dL	0.8 (SD=0.4)	NR	NR
		UDCA	NR		608 (SD=145)	NR	NR		0.9 (SD=0.4)	NR	NR
Jones, et al^44^ (NCT02609048)	Week 12	Placebo + UDCA	12	U/L %	NR	NR	Mean % CfB: −2% (SD=16)	%	NR	NR	Mean % CfB: −4.9%
		Seladelpar 50 mg + UDCA	13		NR	NR	Mean % CfB: −54% (SD=14)		NR	NR	Mean % CfB: −17.8%
		Seladelpar 200 mg + UDCA	10		NR	NR	Mean % CfB: −63% (SD=8)		NR	NR	Mean % CfB: 2.9%
Kanda, et al^45^	Month 6	Bezafibrate + UDCA	NR	IU/L	700 (SD=270)	390	NR	NR	NR	NR	NR
		UDCA alone	NR		550 (SD=220)	540	NR	NR	NR	NR	NR
Kurihara, et al^46^	Month 12	UDCA	12	NR	NR	NR	NR	NR	NR	NR	NR
		Bezafibrate + UDCA	12	NR	NR	NR	NR	NR	NR	NR	NR
Li 2022 (ChiCTR1800020160)	Month 12	Fenofibrate + UDCA	24	U/L	Median: 174	NR	NR	NR	Median: 15.45	NR	NR
		UDCA alone	24		Median: 221	NR	NR	NR	Median: 14.05	NR	NR
Liberopoulos^47^	Week 8	Fenofibrate + UDCA	6	IU/L, %	195 (SD=73)	132 (SD=70)	Mean % CfB: −32.6%	IU/L, %	0.7 (SD=0.14)	0.64 (SD=0.29)	Mean % CfB: −8.5%
		UDCA alone	4		232 (SD=74)	245 (SD=81)	Mean % CfB: 5.6%		0.73 (SD=0.18)	0.65 (SD=0.24)	Mean % CfB: −11%
Lim, et al^48^	Month 18	Azathioprine, prednisone + UDCA	12	IU/L	239	152 (SE=32)	NR	μmol/L	16 (SE=2)	16 (SE=2)	NR
		Placebo + UDCA	12		241	235 (SE=35)	NR	μmol/L	16 (SE=2)	17 (SE=3)	NR
Mayo^49^ (NCT02026401)		Aldafermin (NGM282) 0.3 mg + UDCA	14	IU/L	277.8 (SD=117.4)	NR	NR	mg/dL	0.82 (SD=0.65)	NR	Mean CfB: −0.2 (SD=0.4)
		Aldafermin (NGM282) 3 mg + UDCA	13		332.7 (SD=122.2)	NR	NR		0.92 (SD=0.5)	NR	Mean CfB: 0 (SD=0.2)
		Placebo + UDCA	15		294.4 (SD=135.9)	NR	NR		0.74 (SD=0.29)	NR	Mean CfB: 0 (SD=0.1)
Nakai, et al^49^	Month 12	UDCA 600 mg	NR	NR	NR	NR	NR	NR	NR	NR	NR
		Bezafibrate + UDCA	NR	NR	NR	NR	NR	NR	NR	NR	NR
NCT01904058	Week 13	LUM001 10 mg + UDCA (cohort A)	17	U/L	288.2 (SD=193.91)	NR	Mean CfB: −15.2 (SD=97.19)	NR	NR	NR	NR
		LUM001 20 mg + UDCA (Cohort B)	20		257.6 (SD=190.38)	NR	Mean CfB: 18.5 (SD=56.21)	NR	NR	NR	NR
		Placebo + UDCA (Cohort A)	10		253.9 (SD=96.83)	NR	Mean CfB: 24.3 (SD=76.48)	NR	NR	NR	NR
		Placebo + UDCA (Cohort B)	12		274.2 (SD=190.85)	NR	Mean CfB: −7.2 (SD=83.7)	NR	NR	NR	NR
	Week 13/early termination	LUM001 10 mg + UDCA (cohort A)	21	U/L	288.2 (SD=193.91)	NR	Mean CfB: −8 (SD=93.02)	NR	NR	NR	NR
		LUM001 20 mg + UDCA (Cohort B)	21		257.6 (SD=190.38)	NR	Mean CfB: 16.4 (SD=55.6)	NR	NR	NR	NR
		Placebo + UDCA (Cohort A)	11		253.9 (SD=96.83)	NR	Mean CfB: 22.9 (SD=72.7)	NR	NR	NR	NR
		Placebo + UDCA (Cohort B)	13		274.2 (SD=190.85)	NR	Mean CfB: −7.9 (SD=80.19)	NR	NR	NR	NR
NCT03226067	Week 24	GKT137831 400 mg once daily	38 (TB: 32)	U/L, %	NR	NR	Mean CfB: −32.5 (SD=65.3); % CfB: −9.7% (SD=21.10)	μmol/L, %	NR	NR	Mean CfB: 0.5 (SD=3.77); % CfB: 5.4% (SD=29.77)
		GKT137831 400 mg twice daily	35 (TB: 27)		NR	NR	Mean CfB: −45.2 (SD=84.41); % CfB: −12.9% (SD=19.55)		NR	NR	Mean CfB: 1.2 (SD=2.71); % CfB: 14.5% (SD=31.6)
		Placebo	37 (TB:32)		NR	NR	Mean CfB: −12.4 (SD=53.48); % CfB: −3.1% (SD=15.99)		NR	NR	Mean CfB: 0.7 (SD=3.23) % CfB: 10.5% (SD=32.27)
NCT03394924	Week 12	EDP-305 1 mg	31	NR	NR	NR	NR	µmol/L	NR	NR	LS Mean CfB: −0.04
		EDP-305 2.5 mg	28	NR	NR	NR	NR		NR	NR	LS Mean CfB: −0.31
		Placebo	9	NR	NR	NR	NR		NR	NR	LS Mean CfB: −0.5
NCT03633227	Month 12	Placebo	1	U/L	Median: 216	NR	Median CfB: −77	μmol/L	Median: 45.38	NR	Median CfB: −14.54
		OCA	1		Median: 267.5	NR	Median CfB: 122		Median: 41.5	NR	Median CfB: 9.12
POISE (NCT01473524)	Month 12	OCA 10 mg	62	U/L	316.3 (SD=103.9)	192.3 (SD=61.3)	Mean CfB: −117.7 (SD=73.7)	mg/dL	0.66 (SD=0.39)	0.57 (SD=0.27)	Mean CfB: −0.07 (SD=0.25); LS Mean CfB: −0.05 (SE=0.04)
		OCA 5–10 mg	64		325.9 (SD=116.2)	219.5 (SD=99.8)	Mean CfB: −103.5 (SD=87)		0.6 (SD=0.33)	0.58 (SD=0.28)	Mean CfB: −0.03 (SD=0.2); LS Mean CfB: −0.02 (SE=0.04)
		Placebo	70		327.5 (SD=115)	321.3 (SD=142.9)	Mean CfB: −7.7 (SD=88)		0.69 (SD=0.42)	0.77 (SD=0.51)	Mean CfB: 0.08 (SD=0.24); LS Mean CfB: 0.12 (SE=0.04)
Poupon, et al^53^	Year 2	Colchicine + UDCA	37	N×ULN	2.4 (SD=0.2)	1.8 (SD=0.2)	NR	μmol/L NR	14 (SD=1.2)	12.8 (SD=1.1)	NR
		Placebo + UDCA	37		2.6 (SD=0.3)	2.1 (SD=0.3)	NR		14.5 (SD=1.2)	15.8 (SD=1.6)	NR
RDG-1/PBC	NR	Placebo	19	%	NR	NR	Mean % CfB: −1.53%	NR	NR	NR	NR
		RhuDex 50 mg	20		NR	NR	Mean % CfB: 3.50%	NR	NR	NR	NR
		RhuDex 100 mg	21		NR	NR	Mean % CfB: 1.75%	NR	NR	NR	NR
		RhuDex 200 mg	19		NR	NR	Mean % CfB: 9.86%	NR	NR	NR	NR
RESPONSE	Month 12	Placebo	65	U/L, %	313.8 (SD=117.7)	NR	Mean CfB: −16.9; LS Mean % CfB: −4.3	mg/dL	0.74 (SD=0.3)	NR	LS Mean CfB: 3.6
		Seladelpar 10 mg	128		314.6 (SD=123.0)	NR	Mean CfB: −133.9; LS Mean % CfB: −42.4		0.77 (SD=0.3)	NR	LS Mean CfB: −0.4
Schattenberg, et al^51^	Week 12	Elafibranor 80 mg + UDCA	15	IU/L	350.6 (SD=152.1)	NR	Mean CfB: −48.3 (SD=14.8)	μmol/L	9.9 (SD=6.7)	9.9 (SD=6.7)	Mean CfB: −0.2 (SD=3.4)
		Elafibranor 120 mg + UDCA	14		263.7 (SD=137.6)	NR	Mean CfB: −40.6 (SD=17.4)		9.7 (SD=5.3)	9.7 (SD=5.3)	Mean CfB: −0.5 (SD=2.8)
		Placebo + UDCA	15		296.2 (SD=115.5)	NR	Mean CfB: 3.2 (SD=14.8)		11.1 (SD=4.4)	11.1 (SD=4.4)	Mean CfB: 0 (SD=3.6)
Silveira, et al^59^	Week 12	Modafinil	17	U/L, %	Median: 142	NR	Median % CfB: 8.3%	mg/dL	Median: 0.5	Median: 0.4	Median CfB: −0.05
		Placebo	19		Median: 156	NR	Median % CfB: 21.4%		Median: 0.5	Median: 0.4	Median CfB: 0.06
Study 213	Month 3	Bezafibrate 200 mg + placebo	19	%	246.7 (SD=69.7)	NR	Mean % CfB: −39.6	mg/dL	0.5 (SD=0.2)	NR	NR
		Bezafibrate 200 mg + OCA	19		277.3 (SD=178.2)	NR	Mean % CfB: −39.8%		0.6 (SD=0.3)	NR	NR
		Bezafibrate 400 mg + placebo	19		277.4 (SD=108.2)	NR	Mean % CfB: −45.1%		0.6 (SD=0.3)	NR	NR
		Bezafibrate 400 mg + OCA	18		344.0 (SD=156.3)	NR	Mean % CfB: −62.3%		0.6 (SD=0.3)	NR	NR
Study 213/Study 214	Week 12	Bezafibrate 100 mg immediate release	NR	%	NR	NR	Mean % CfB: −32.2%	%	NR	NR	Mean % CfB: −5.5
		Bezafibrate 200 mg immediate release	NR		NR	NR	Mean % CfB: −39.9%		NR	NR	Mean % CfB: −14.1%
		Bezafibrate 400 mg immediate release	NR		NR	NR	Mean % CfB: −53.2%		NR	NR	Mean % CfB: −1.7%
		OCA 5 mg + bezafibrate 100 mg immediate release	NR		NR	NR	Mean % CfB: −44.1%		NR	NR	Mean % CfB: −15.5%
		OCA 5 mg + bezafibrate 400 mg immediate release	NR		NR	NR	Mean % CfB: −65.4%		NR	NR	Mean % CfB: −21.1
		OCA 5–10 mg + bezafibrate 200 mg immediate release	NR		NR	NR	Mean % CfB: −40.3%		NR	NR	Mean % CfB: −20.6%
		Bezafibrate 400 mg slow release	NR		NR	NR	Mean % CfB: −46.4%		NR	NR	Mean % CfB: −14.0%
		OCA 5–10 mg + bezafibrate 400 mg sustained release	NR		NR	NR	Mean % CfB: −60.6%		NR	NR	Mean % CfB: −24.7%
Tanaka, et al^58^ (NCT03092765)	Week 12	E6011 10 mg/kg/mo	4	IU/L, %	794 (SD=180)	NR	Mean CfB: 0.65 (SD=12.9)	NR	NR	NR	NR
		E6011 15 mg/kg/mo	4		976 (SD=275)	NR	Mean CfB: 1.23 (SD=8.8)	NR	NR	NR	NR
		E6011 10 mg/kg/EOW	14		1080 (SD=461)	NR	Mean CfB: 1.19 (SD=15.1)	NR	NR	NR	NR
		Placebo	7		987 (SD=259)	NR	Mean CfB: 0.45 (SD=15.8)	NR	NR	NR	NR
TRANSFORM (NCT05014672)	Week 24	Setanaxib 1200 mg	24	Ratio	NR	NR	LS mean CfB: 0.89 (*p*=0.0206 vs. placebo)	NR	NR	NR	NR
		Setanaxib 1600 mg	25		NR	NR	LS mean CfB: 0.84 (*p*=0.0057 vs. placebo)	NR	NR	NR	NR
		Placebo	27		NR	NR	LS mean CfB: 1.03	NR	NR	NR	NR
		Setanaxib doses combined	49		NR	NR	LS mean CfB: 0.87 (*p*=0.0039 vs. placebo)	NR	NR	NR	NR
Xiang, et al^55^ (NCT03345589)	Month 12	UDCA 13–15 mg	36	IU/L, %	Median: 281	NR	Median % CfB: 19.89%	μmol/L, %	Median: 17.4	NR	Median CfB: 8.3 Median % CfB: 17.4%
		UDCA 18–22 mg	37		Median: 238	NR	Median % CfB: 21.16%		Median: 15.3	NR	Median CfB: 2.25 Median % CfB: 15.3%

*Note:* Values presented as means, unless otherwise specified.

Abbreviations: ALP, alkaline phosphatase; CfB, change from baseline; EOW, every other week; IU, international unit; LS, least squares; NR, not reported; OCA, obeticholic acid; SD, standard deviation; SE, standard error; TB, total bilirubin; UDCA, ursodeoxycholic acid; ULN, upper limit of normal.

In addition, normalization of ALP (ie, reduction of ALP to the study-assigned ULN value or lower) was reported in 12 studies (27.3%), and categorical changes in ALP were reported in 7 studies (15.9%). Of the studies that reported categorical changes from baseline, the proportion of patients with reductions in ALP of ≥20% and ≥40% from baseline was most frequently used [6 studies each (13.6%)]. Other categories included a ≥15% reduction from baseline and various thresholds, which were reported in 5 studies (11.4%) and 1 study (2.3%), respectively.

#### Total bilirubin

TB was reported in 31 studies (70.5%), of which 30 (68.2%) studies reported baseline and endpoint values, and/or change from baseline values (Table 4). Changes from baseline in TB were reported for most treatments; changes were reported as mean values in 15 studies (34.1%) and as median values in 4 studies (9.1%; Table 4). Similarly to ALP, there was variability in how change from baseline was expressed across studies, where absolute and relative changes from baseline, or a combination of both, were used (Table 4).

Normalization of TB was reported in 4 studies (9.1%),^52^,^64^,^65^,^66^ and 1 study (2.3%) reported proportions of patients with categorical changes in TB.^52^

#### Gamma-glutamyl transferase

GGT was reported in 25 studies (56.8%), of which 24 (54.5%) studies reported baseline and endpoint values, and/or change from baseline (Table 5). Rates of GGT normalization were reported in 2 studies (4.5%), both of which defined ULN as <65 U/L; one investigated treatment with tropifexor and the other investigated bezafibrate + OCA.^64^,^65^ In addition, 1 study (2.3%) reported proportions of patients with categorical changes in GGT.^52^

**TABLE 5:** Change from baseline in gamma-glutamyl transferase (GGT) and albumin

Study name	Timepoint	Treatment arm	Denominator	GGT				Albumin			
				Units	Baseline value, mean unless otherwise specified	Value at timepoint, mean unless otherwise specified	Change from baseline	Units	Baseline value, mean unless otherwise specified	Value at timepoint, mean unless otherwise specified	Change from baseline
BEZURSO^36^ (NCT01654731)	Month 24	Bezafibrate + UDCA	50	NR	NR	NR	Median CfB: −38	NR	NR	NR	Median CfB: 0
		Placebo + UDCA	50	NR	NR	NR	Median CfB: 7	NR	NR	NR	Median CfB : −3
Bowlus, et al^56^ (NCT02955602)	Week 52	Seladelpar 2 mg	10	U/L	254.5	176.1	Mean CfB: −78.6; % CfB: −32.3%	NR	NR	NR	NR
		Seladelpar 5 mg	42		243.8	163.6	Mean CfB: −91.4; % CfB: −34.2%	NR	NR	NR	NR
		Seladelpar 10 mg	48		239.3	155.1	Mean CfB: −88.1; % CfB: −32.5%	NR	NR	NR	NR
COBALT (NCT02308111)	Month 12	OCA 5 mg titrated to 10 mg	126	U/L	NR	NR	Mean CfB: −152.2	g/L	NR	NR	LS Mean CfB: −0.3
	Month 12	Placebo	138		NR	NR	Mean CfB: −63.4		NR	NR	LS Mean CfB: −0.3
	Month 24	OCA 5 mg titrated to 10 mg	98		NR	NR	Mean CfB: −175.6		NR	NR	LS Mean CfB: −0.1
	Month 24	Placebo	102		NR	NR	Mean CfB: −127.7		NR	NR	LS Mean CfB: −1.1
ELATIVE (NCT04526665)	Week 52	Placebo	47	U/L	NR	NR	Mean CfB: −17.4	g/L	NR	NR	LS Mean CfB: −0.9
		Elafibranor 80 mg	94		−44.0	NR	Mean CfB: −47.6		NR	NR	LS Mean CfB: 0.7
		Placebo	53		220.0	NR	NR	g/dL	NR	NR	Mean CfB: −0.12 (SD=0.27)
		Elafibranor 80 mg	108		213.3	NR	NR		NR	NR	Mean CfB: 0.1 (SD=0.28)
ENHANCE (NCT03602560)	Month 3	Seldapar 5 mg	54	mg/dL, %	231.3	NR	% CfB: −30.3%	NR	NR	NR	NR
		Seldapar 10 mg	53		243.1	NR	% CfB: −36.4%	NR	NR	NR	NR
		Placebo	56		228.9	NR	% CfB: −6.5%	NR	NR	NR	NR
EPICS (NCT03112681)	Week 16	Saroglitazar 4 mg	13	U/L	279.9	NR	NR	NR	NR	NR	NR
		Saroglitazar 2 mg	14		383	NR	NR	NR	NR	NR	NR
		Placebo	10		188.6	NR	NR	NR	NR	NR	NR
Gonzalez-Koch, et al^38^	Week 48	Methotrexate + UDCA	13	U/L	373	NR	Mean CfB: −297	g/dL	4.33 (SD=0.34)	NR	Mean CfB: 0.05
		Placebo + UDCA	12		314	NR	Mean CfB: −235		4.28 (SD=0.48)	NR	Mean CfB: −0.11
HAART (NCT01614405)	Month 6	TDC/FTC/LPRr + UDCA	5	U/L	Median: 412	Median: 502	Median CfB: 90	NR	NR	NR	NR
		Placebo + UDCA	6		Median: 513	Median: 374	Median CfB: −139	NR	NR	NR	NR
Hegade, et al^57^ (NCT01899703)	Day 14	Post GSK2330672 sequence 1	11	IU/L	211	197.8	NR	g/L	41.9 (SD=4.2)	40.7 (SD=3.9)	NR
		Post GSK2330672 placebo 1	11		211	182.9	NR		41.9 (SD=4.2)	39.5 (SD=3.6)	NR
		Post GSK2330672 sequence 2	10		211	312.3	NR		41.9 (SD=4.2)	40.9 (SD=3.1)	NR
		Post GSK2330672 placebo 2	10		211	349.3	NR		41.9 (SD=4.2)	41.6 (SD=2.0)	NR
Iwasaki, et al^43^	Week 52	Bezafibrate + UDCA	NR	IU/L	254	NR	NR	NR	NR	NR	NR
		UDCA	NR		145	NR	NR				
Jones, et al^44^ (NCT02609048)	Week 12	Placebo + UDCA	12	%	NR	NR	% CfB: −1.8%	NR	NR	NR	NR
		Seladelpar 50 mg + UDCA	13		NR	NR	% CfB: −42.7%	NR	NR	NR	NR
		Seladelpar 200 mg + UDCA	10		NR	NR	% CfB: −47.1%	NR	NR	NR	NR
Kanda, et al^45^	Month 6	Bezafibrate + UDCA	NR	NR	46 (SD=30)	NR	NR	NR	NR	NR	NR
		UDCA alone	NR	NR	34 (SD=14)	NR	NR	NR	NR	NR	NR
Li 2022 (ChiCTR1800020160)	Month 12	Fenofibrate + UDCA	24	U/L	Median: 218	NR	Median CfB <0.001	NR	NR	NR	NR
		UDCA alone	24		Median: 169	NR	NR	NR	NR	NR	NR
Liberopoulos^47^	Week 8	Fenofibrate + UDCA	6	IU/L, %	Median: 96	Median: 54	Median CfB <0.049; % CfB: −44%	NR	NR	NR	NR
		UDCA alone	4		Median: 112	Median: 120	Median % CfB: 1%	NR	NR	NR	NR
Mayo^49^ (NCT02026401)	Day 28	Aldafermin (NGM282) 0.3 mg + UDCA	14	IU/L	NR	NR	Mean CfB: −28.2	g/L	NR	NR	Mean CfB: 0 (SD=0.2)
		Aldafermin (NGM282) 3 mg + UDCA	13		NR	NR	Mean CfB: −50.8		NR	NR	Mean CfB: 0.1 (SD=0.2)
		Placebo + UDCA	15		NR	NR	Mean CfB: −5.6		NR	NR	Mean CfB: 0 (SD=0.3)
NCT03226067	Week 24	GKT137831 400 mg once daily	38	U/L	NR	NR	Mean CfB: −17.9; CfB: −4.9%	NR	NR	NR	NR
		GKT137831 400 mg twice daily	35		NR	NR	Mean CfB: −43.6; % CfB: −19.0%	NR	NR	NR	NR
		Placebo	37		NR	NR	Mean CfB: −10.7; % CfB: −8.4%	NR	NR	NR	NR
NCT03394924	Week 12	EDP-305 1 mg	31	U/L	NR	NR	Mean CfB: −95.91	NR	NR	NR	NR
		EDP-305 2.5 mg	28		NR	NR	Mean CfB: −124.55	NR	NR	NR	NR
		Placebo	9		NR	NR	Mean CfB: −9.42	NR	NR	NR	NR
NCT03633227	Week 48	Placebo	6	U/L	Median: 98	NR	Median CfB: −9	NR	Median: 34.5	NR	Median CfB: −0.5
		OCA	6		Median: 103	NR	Median CfB: −15	NR	Median: 33	NR	Median CfB: 0.5
POISE (NCT01473524)	Month 12	OCA 10 mg	62	U/L	261.1	91.9	Mean CfB: −177.7; LS mean= −176.7	g/L	43.7 (SD=2.7)	43.1 (SD=3.3)	Mean CfB: −0.9; LS Mean CfB: −0.9
		OCA 5–10 mg	64		252.8	114.2	Mean CfB: −138.2; LS mean=−140.8		43 (SD=3.1)	42.7 (SD=3.5)	Mean CfB: −0.5; LS Mean CfB: −0.6
		Placebo	70		309.6	301.8	Mean CfB: −8.2; LS mean=6.7		42.8 (SD=3.1)	41.8 (SD=3.6)	Mean CfB: −1.1; LS Mean CfB: −1.2
Poupon, et al^53^	Year 2	Colchicine + UDCA	37	ULN	4.67	3.66	NR	g/L	39.6 (SD=0.9)	39.8 (SD=0.9)	NR
		Placebo + UDCA	37		5.78	4.86	NR		40.6 (SD=0.7)	39.8 (SD=0.8)	NR
RESPONSE	Month 12	Placebo	65	U/L	287.5	NR	Mean CfB: −18.3	NR	NR	NR	NR
		Seladelpar 10 mg	128		269.0	NR	Mean CfB: −108.0	NR	NR	NR	NR
Schattenberg, et al^51^	Week 12	Elafibranor 80 mg + UDCA	15	IU/L	282.3	190.8	Mean CfB: −91.5	g/L	41 (SD=3.3)	43.2 (SD=3.6)	Mean CfB: 2.2 (SD=2.5)
		Elafibranor 120 mg + UDCA	14		158.5	96.6	Mean CfB: −61.9		41.1 (SD=2.7)	43.4 (SD=3.6)	Mean CfB: 2.3 (SD=2.7)
		Placebo + UDCA	15		229.6	230.2	Mean CfB: 0.6		42.5 (SD=2)	42.5 (SD=1.8)	Mean CfB: 0 (SD=2.2)
Silveira, et al^59^	Week 12	Modafinil	17	NR	NR	NR	NR	g/dL	Median 4.2	Median 4.2	Median 0.04
		Placebo	19	NR	NR	NR	NR		Median 4.2	Median 4.2	Median 0.03
Study 213	Month 6	Bezafibrate 400 mg + OCA	18	NR	NR	NR	Mean CfB: −81.6	NR	NR	NR	NR
Xiang, et al^55^ (NCT03345589)	Month 12	UDCA 13–15 mg	36	IU/L, %	Median: 220	NR	Median % CfB: −27.9%	g/L, %	Median: 43.3	NR	Median % CfB: −1.88
		UDCA 18–22 mg	37		Median: 161	NR	Median % CfB: 31.86%		Median: 45.4	NR	Median % CfB: −0.44

*Note:* Values presented as means, unless otherwise specified.

Abbreviations: GGT, gamma-glutamyl transferase; IU, international units; LS, least squares; NR, not reported; OCA, obeticholic acid; SD, standard deviation; SE, standard error; UDCA, ursodeoxycholic acid; ULN, upper limit of normal.

#### Albumin

Albumin was reported in 12 studies (27.3%); change from baseline was reported in 10 studies, and only baseline and/or endpoint values were reported in 2 studies (Table 5). Of the 10 studies that reported change from baseline, mean or LS mean absolute change was reported in 6 studies, median absolute change in 3 studies, and mean relative change in 1 study (Table 5). Rates of albumin normalization were only reported in 1 study, which defined a normal range as 3.5–5.2 g/dL for ages 18–60 years and 3.2–4.6 g/dL for ages 61–91 years.^51^

### Liver fibrosis

Non-invasive tests (NITs) for liver fibrosis were reported through liver stiffness measurement (LSM) and enhanced liver fibrosis (ELF) in 7 (15.9%) and 5 (11.4%) studies, respectively (Table 6), where 5 studies reported both LSM and ELF.

**TABLE 6:** Change from baseline in liver fibrosis

Liver fibrosis measure	Study name	Timepoint	Units	Treatment arm	Denominator	Value at timepoint, mean unless otherwise specified	Change from baseline, mean unless otherwise specified
LSM	BEZURSO^36^ (NCT01654731)	Month 24	%	Bezafibrate + UDCA	50	NR	% CfB: −15%
				Placebo + UDCA	50	NR	% CfB: 22%
	COBALT (NCT02308111)	Month 12	kPa	OCA 5 mg titrated to 10 mg	88	0.25^a^	NR
				Placebo	90	0.90^a^	NR
		Month 24		OCA 5 mg titrated to 10 mg	67	−0.60^a^	NR
				Placebo	67	1.50^a^	NR
	ELATIVE (NCT04526665)	Week 52	kPa	Placebo	44	NR	LS Mean CfB: 0.3
				Elafibranor 80 mg	90	NR	LS Mean CfB: 0.2
	NCT03226067	Week 24	kPa, %	GKT137831 400 mg once daily	33	NR	Mean CfB: −0.5 (SD=5.24); % CfB: 3.3%
				GKT137831 400 mg twice daily	26	NR	Mean CfB: −0.3 (SD=7.9); % CfB: 7.9%
				Placebo	32	NR	Mean CfB: 0.7; (SD=3.63); % CfB: 10.1%
	POISE (NCT01473524)	Month 12	kPa	OCA 10 mg	73	11.18 (SD=12.477)	LS Mean CfB: 0.46 (SE=1.39)
				OCA 5–10 mg	70	10.06 (SD=6.67)	LS Mean CfB: 1.28 (SE=1.34)
				Placebo	73	13.49 (SD=10.62)	LS Mean CfB: 1.82 (SE=1.35)
	RESPONSE	Month 12	kPa	Placebo	55	9.84 (SD=10)	% CfB: 1.34%; LS Mean CfB: 7.56
				Seladelpar 10 mg	199	9.76 (SD=8)	% CfB: 0.24%; LS Mean CfB: 4.39
	TRANSFORM (NCT05014672)	Week 24	NR	Setanaxib 1200 mg	24	NR	Geometric least squares mean: 0.78
				Setanaxib 1600 mg	25	NR	Geometric least squares mean: 0.88
				Placebo	27	NR	Geometric least squares mean: 0.92
ELF	BEZURSO (NCT01654731)	Month 24	NR	Bezafibrate + UDCA	50	NR	% CfB: −1%
				Placebo + UDCA	50	NR	% CfB: 3%
	COBALT (NCT02308111)	Month 12	NR	OCA 5 mg titrated to 10 mg	126	0.10^a^	NR
				Placebo	127	0.10^a^	NR
		Month 24		OCA 5 mg titrated to 10 mg	95	0.00^a^	NR
				Placebo	95	0.20^a^	NR
	ELATIVE (NCT04526665)	Week 52	NR	Placebo	44	NR	LS Mean CfB: 0.2
				Elafibranor 80 mg	89	NR	LS Mean CfB: 0.1
	POISE (NCT01473524)	Month 12	NR	OCA 10 mg	73	9.77 (SD=1.177)	LS Mean CfB: 0.2 (SE=0.12)
				OCA 5–10 mg	70	9.73 (SD=0.994)	LS Mean CfB: 0.24 (SE=0.12)
				Placebo	73	10.12 (SD=1.285)	LS Mean CfB: 0.33 (SE=0.13)
	RESPONSE	Month 12	NR	Placebo	65	10.21 (SD=0.99)	Mean CfB: −0.002 (SD=0.56); % CfB: 0.007%
				Seladelpar 10 mg	128	10.14 (SD=1.07)	Mean CfB: 0.06 (SD=0.60); % CfB: 0.74%
FIB-4 Fibrosis Index	NCT03394924	Week 12	NR	EDP-305 1 mg	24	NR	LS Mean CfB: −0.14
			NR	EDP-305 2.5 mg	20	NR	LS Mean CfB: −0.05
			NR	Placebo	8	NR	LS Mean CfB: 0.21
	Xiang, et al^55^ (NCT03345589)	Month 12	NR	UDCA 13–15 mg	NR	2.874^a^	NR
			NR	UDCA 18–22 mg	NR	2.036^a^	NR
Procollagen III animo terminal peptide (PIIIP)	NCT03394924	Week 12	μg/L	EDP-305 1 mg	28	NR	LS Mean CfB: −0.08
				EDP-305 2.5 mg	21	NR	LS Mean CfB: −0.77
				Placebo	9	NR	LS Mean CfB: 3.01
Tissue inhibitor of metalloproteinase 1 (TIMP 1)	NCT03394924	Week 12	μg/L	EDP-305 1 mg	28	NR	LS Mean CfB: −16.29
				EDP-305 2.5 mg	21	NR	LS Mean CfB: −20.9
				Placebo	9	NR	LS Mean CfB: 25.66
N-terminal type III collagen propeptide (PRO-C3)	NCT03394924	Week 12	μg/L	EDP-305 1 mg	19	NR	LS Mean CfB: −0.67
				EDP-305 2.5 mg	18	NR	LS Mean CfB: 2.33
				Placebo	8	NR	LS Mean CfB: 9.06
AST to Platelet Ratio Index score	NCT03394924	Week 12	Index	EDP-305 1 mg	24	NR	LS Mean CfB: −0.16
				EDP-305 2.5 mg	20	NR	LS Mean CfB: −0.12
				Placebo	8	NR	LS Mean CfB: 0.22
Hyaluronic acid	NCT03394924	Week 12	μg/L	EDP-305 1 mg	NR	NR	LS Mean CfB: 1.17
				EDP-305 2.5 mg	NR	NR	LS Mean CfB: −1.16
				Placebo	NR	NR	LS Mean CfB: 27.83
Fibrosis scored on a qualitative scale (0–3)	Wolfhagen, et al^54^	Month 12	NR	Prednisone, azathioprine + UDCA	26	2 (SD=1.1)	*p*=0.22
			NR	Placebo + UDCA	24	2.55 (SD=1)	*p*=1

Values presented as means, unless otherwise specified.

^a^ Median values reported.

Abbreviations: LS, least squares; NR, not reported; OCA, obeticholic acid; PIIP, procollagen III amino-terminal peptide; PRO-C3, N-terminal type III collagen propeptide; SD, standard deviation; SE, standard error; TIMP 1, tissue inhibitor of metalloproteinase 1; UDCA, ursodeoxycholic acid.

Of the studies that reported LSM, OCA was investigated in 2 studies, and bezafibrate, elafibranor, GKT137831, seladelpar, and setanaxib were investigated in 1 study each. Overall, changes from baseline in non-invasive markers of fibrosis were small (Table 6). ELF scoring was reported for OCA in 2 studies, and bezafibrate, elafibranor, and seladelpar in 1 study each (Table 6). There were differences in how measures of liver fibrosis were reported, where some studies evaluated mean, or LS mean change from baseline (3 studies reporting LSM and ELF each) or as geometric LS mean (1 study reporting LSM), and others evaluated percentage change from baseline (3 studies reporting LSM and 2 studies reporting ELF).

Other measures of liver fibrosis included AST to Platelet Ratio Index (APRI), Fibrosis-4 (FIB-4) score, and markers including procollagen III amino-terminal peptide (PIIIP), tissue inhibitor of metalloproteinase 1 (TIMP 1), and N-terminal type III Collagen propeptide (PRO-C3; Table 6).

### Pruritus

Alongside changes in biochemical markers and measures of liver fibrosis, PROs are available to evaluate treatment effects on debilitating symptoms of PBC, including pruritus. Pruritus PROs were reported in 23 studies (52.3%) using a variety of assessment tools to measure the severity or intensity of pruritus, or the impact of pruritus on patients’ HRQoL (Table 7). The visual analog scale (VAS) and the Worst-Itch Numeric Rating Scale (WI NRS) were the most frequently used to measure itch intensity, reported in 12 studies (27.3%) and 6 studies (13.6%), respectively (Table 7). In addition, the Itch domain of the PBC-40 and the 5-dimension (5D)-Itch questionnaire were most frequently used to measure pruritus-related impact on HRQoL, reported in 14 studies (31.8%) and 8 studies (18.2%), respectively (Table 7). Other measures, including bothersome itch, itch intensity (NRS), itch reporter outcome (RO), Patient Global Impression of Change (PGIC) and Severity (PGIS), monthly itch score, and sleep interference NRS, were less commonly used.

**TABLE 7:** Change from baseline in pruritus

Reporting	Study name	Timepoint	Treatment arm	Denominator (pruritus)	Baseline value	Value at timepoint	Change from baseline, mean unless otherwise specified
VAS	BEZURSO (NCT01654731)	24 mo	Bezafibrate + UDCA	50	NR	NR	Mean CfB: −1
			Placebo + UDCA	50	NR	NR	Mean CfB: 0.04
	Bowlus, et al^56^ (NCT02955602)	Week 52	Seladelpar 2 mg	10	NR	NR	Mean CfB: −3.3 (SD=11.7)
			Seladelpar 5 mg	42	NR	NR	Mean CfB: −9.6 (SD=22.5)
			Seladelpar 10 mg	48	NR	NR	Mean CfB: −16.5 (SD=23)
	FITCH (NCT02701166)	Day 21	Bezafibrate	11	NR	NR	NR
			Placebo	15	NR	NR	NR
	Hirschfield, et al^40^	Month 36	Budesonide + UDCA	40	1.2^a^	NR	Mean CfB: −0.3 (SD=2.3)
			Placebo + UDCA	22	2.2^a^	NR	Mean CfB: 0.6 (SD=3.1)
	Jones, et al^44^ (NCT02609048)	Week 12	Placebo + UDCA	12	18 (SD=18)	27 (SD=27)	NR
			Seladelpar 50 mg + UDCA	13	21 (SD=22)	21 (SD=24)	NR
			Seladelpar 200 mg + UDCA	10	33 (SD=25)	31 (SD=31)	NR
	Mayo^49^ (NCT02026401)	Day 28	Aldafermin (NGM282) 0.3 mg + UDCA	14	NR	NR	Mean CfB: −3.8 (SD=12.9)
			Aldafermin (NGM282) 3 mg + UDCA	13	NR	NR	Mean CfB: −12.6 (SD=24.9)
			Placebo + UDCA	15	NR	NR	Mean CfB: −6.2 (SD=15.6)
	NCT02955602	Week 52	Seladelpar 5 mg	49	NR	5^a^	NR
			Seladelpar 10 mg	52	NR	5^a^	NR
	NCT03226067	Week 24	GKT137831 400 mg QD	38	NR	NR	Mean CfB: −1 (SE=2.32); % CfB: −36.9 (SE=58.6)
			GKT137831 400 mg BID	34	NR	NR	Mean CfB: 0.1 (SE=2.3); % CfB: −0.3 (SE=92.71)
			Placebo	37	NR	NR	Mean CfB: 0.7 (SE=1.83); % CfB: 27.3 (SE=72.88)
	NCT03394924	Week 12	EDP-305 1 mg	26	NR	NR	LS Mean CfB: 0.55
			EDP-305 2.5 mg	22	NR	NR	LS Mean CfB: 13.64
			Placebo	9	NR	NR	LS Mean CfB: −11.93
	POISE (NCT01473524)	Month 12	OCA 10 mg	73	NR	NR	NR
			OCA 5–10 mg	70	NR	NR	NR
			Placebo	73	NR	NR	NR
	Schattenberg, et al^51^	NR	Elafibranor 80 mg + UDCA	10	NR	NR	% CfB: −24^a^
			Elafibranor 120 mg + UDCA	10	NR	NR	% CfB: −49^a^
			Placebo + UDCA	10	NR	NR	% CfB: −7^a^
			Elafibranor 80 mg + UDCA	14	NR	NR	NR
			Elafibranor 120 mg + UDCA	14	NR	NR	NR
			Placebo + UDCA	15	NR	NR	NR
	Schramm, et al^52^	Day 84	Tropifexor 30 µg + UDCA	9	NR	NR	Median CfB: −5.14
			Tropifexor 60 µg + UDCA	9	NR	NR	Median CfB: 0.89
			Tropifexor 90 µg + UDCA	12	NR	NR	Median CfB: −6.44
			Tropifexor 150 µg + UDCA	8	NR	NR	Median CfB: −5.14
			Placebo + UDCA	20	NR	NR	Median CfB: 11.79
WI NRS	ELATIVE (NCT04526665)	Week 52	Elafibranor 80 mg	32	NR	NR	LS Mean CfB: −1.93
			Placebo	12	NR	NR	LS Mean CfB: −1.15
	ENHANCE (NCT03602560)	Month 3	Seladelpar 5	17	6.1 (SD=1.4)	NR	Mean CfB: −1.95 (SD=2.226); LS Mean CfB: −2.01
			Seladelpar 10 mg	18	6.2 (SD=1.4)	NR	Mean CfB: −3.01 (SD=1.952); LS Mean CfB : −3.14
			Placebo	18	6.1 (SD=1.2)	NR	Mean CfB: −1.44 (SD=1.831); LS Mean CfB: −1.55
	Hegade, et al^57^ (NCT01899703)	Day 14	GSK2330672 sequence 1	11	NR	2.91 (SE=0.733)	% CfB: −36% (SE=11)
			Placebo sequence 1	11	NR	3 (SE=0.733)	% CfB: −42 (SD=11)
			Placebo sequence 2	10	NR	4.86 (SE=0.757)	% CfB: −25 (SD=11)
			GSK2330672 sequence 2	10	NR	1.52 (SE=0.757)	% CfB: −76 (SD=11)
	RESPONSE	Month 12	Placebo^b^	65	3 (SD=3.0)	NR	LS Mean CfB: −0.5
			Seladelpar 10 mg^b^	128	3 (SD=2.8)	NR	LS Mean CfB: −1.4
			Placebo^c^	65	6.6 (SD=1.4)	NR	LS Mean CfB: −1.5
			Seladelpar 10 mg^c^	128^c^	6.1 (SD=1.4)	NR	LS Mean CfB: −3.3
	TRANSFORM (NCT05014672)	Week 24	Setanaxib 1200 mg	13	NR	NR	Mean CfB: −0.23 (SD=2.28)
			Setanaxib 1600 mg	15	NR	NR	Mean CfB: 0.61 (SD=1.39)
			Placebo	16	NR	NR	Mean CfB: −0.56 (SD=2.27)
PBC-40 Itch	Bowlus, et al^56^ (NCT02955602)	Week 52	Seladelpar 2 mg	10	NR		Mean CfB: 0.4 (SD=2.7)
			Seladelpar 5 mg	42	NR	NR	Mean CfB: −1.3 (SD=3.2)
			Seladelpar 10 mg	48	NR	NR	Mean CfB: −1.4 (SD=3.7)
	ELATIVE (NCT04526665)	Week 52	Placebo	16	NR	NR	LS Mean CfB: −0.1
			Elafibranor 80 mg	42	NR	NR	LS Mean CfB: v2.5
	EPICS (NCT03112681)	Week 16	Saroglitazar 4 mg	13	NR	NR	Mean CfB: −1.52
			Saroglitazar 2 mg	14	NR	NR	Mean CfB: −1.08
			Placebo	10	NR	NR	Mean CfB: −2.31
	GLIMMER (NCT02966834)	Day 1	All patients	NR	NR	10^a^ (SD=3.06)	NR
	HAART (NCT01614405)	Month 6	TDC/FTC/LPRr + UDCA	5	NR	9.5^a^	*p*=0.285
			Placebo + UDCA	6	NR	7^a^	*p*=0.916
	Hegade, et al^57^ (NCT01899703)	Day 14	GSK2330672 sequence 1	11	NR	NR	% CfB: −13% (SE=8)
			Placebo sequence 1	11	NR	NR	% CfB: −18% (SE=8)
			Placebo sequence 2	10	NR	NR	% CfB: −16% (SE=8)
			GSK2330672 sequence 2	10	NR	NR	% CfB: −48%(SE=8)
	Hirschfield, et al^40^	Month 36	Budesonide + UDCA	40	NR	NR	Mean CfB: v0.5 (SD=2.8)
			Placebo + UDCA	22	NR	NR	Mean CfB: 0.5 (SD=1.2)
	NCT03226067	Week 24	GKT137831 400 mg QD	37	NR	NR	Mean CfB: −0.4 (SE=0.5)
			GKT137831 400 mg BID	30	NR	NR	Mean CfB: 0.1 (SE=0.5)
			Placebo	36	NR	NR	Mean CfB: 0.1 (SE=0.5)
			GKT137831 400 mg QD	38	NR	NR	% CfB: −11.4 (SE=52.75)
			GKT137831 400 mg BID	34	NR	NR	% CfB: −9.5 (SE=41.83)
			Placebo	37	NR	NR	% CfB: −6.8 (SE=40.62)
	NCT03394924	Week 12	EDP-305 1 mg	28	NR	NR	LS Mean CfB: 0.18
			EDP-305 2.5 mg	21	NR	NR	LS Mean CfB: 1.74
			Placebo	9	NR	NR	LS Mean CfB: −1.73
	POISE (NCT01473524)	Month 12	OCA 10 mg	73	NR	NR	NR
			OCA 5–10 mg	70	NR	NR	NR
	Schattenberg, et al^51^	NR	Elafibranor 80 mg + UDCA	1415	NR	NR	% CfB: −0.9% (SD=6.19)
		NR	Elafibranor 120 mg + UDCA	1416	NR	NR	% CfB: −4.1% −0.56(SD=6.56)
		NR	Placebo + UDCA	15	NR	NR	% CfB: 2.1% (SD=5.78)
	Schramm, et al^52^	Day 84	Tropifexor 30 µg + UDCA	9	NR	NR	Median CfB: −2
			Tropifexor 60 µg + UDCA	9	NR	NR	Median CfB: −1
			Tropifexor 90 µg + UDCA	12	NR	NR	Median CfB: v3.5
			Tropifexor 150 µg + UDCA	8	NR	NR	Median CfB: −1
			Placebo + UDCA	18	NR	NR	Median CfB: 1.5
	Tanaka, et al^58^ (NCT03092765)	Week 12	E6011 10 mg/kg/mo	4	9.0 (SE=1.8)	NR	Mean CfB: 0.0 (SD=0.8)
			E6011 15 mg/kg/mo	4	8.8 (SE=3.5)	NR	Mean CfB: −1.3 (SD=3.7)
			E6011 10 mg/kg/EOW	14	5.6 (SE=3.4)	NR	Mean CfB: 0.6 (SD=3.0)
			Placebo	7	6.6 (SE=4.8)	NR	Mean CfB: −0.4 (SD=2.2)
	TRANSFORM (NCT05014672)	Week 24	Setanaxib 1200 mg	18	NR	NR	Mean CfB: −1.11 (SD=2.11)
			Setanaxib 1600 mg	19	NR	NR	Mean CfB: 0.32 (SD=2.65)
			Placebo	20	NR	NR	Mean CfB: −0.70 (SD=3.11)
5D-Itch	ELATIVE (NCT04526665)	Week 52	Placebo	16	NR	NR	LS Mean CfB: −1.2
			Elafibranor 80 mg	42	NR	NR	LS Mean CfB: −4.2
	Hegade, et al^57^ (NCT01899703)	Day 14	GSK2330672 sequence 1	11	NR	NR	% CfB: −23% (SE=7)
			Placebo sequence 1	11	NR	NR	% CfB: −16% (SE=7)
			Placebo sequence 2	10	NR	NR	% CfB: −14% (SE=7)
			GSK2330672 sequence 2	10	NR	NR	% CfB: −47% (SE=7)
	Jones, et al^44^ (NCT02609048)	Week 12	Placebo + UDCA	12	NR	11 (SD=4)	NR
			Seladelpar 50 mg + UDCA	13	NR	12 (SD=6)	NR
			Seladelpar 200 mg + UDCA	10	NR	11 (SD=4)	NR
	Mayo^49^ (NCT02026401)	Day 28	Aldafermin (NGM282) 0.3 mg + UDCA	14	NR	NR	Mean CfB: −1.4 (SD=4.3)
			Aldafermin (NGM282) 3 mg + UDCA	13	NR	NR	Mean CfB: −2.1 (SD=4.7)
			Placebo + UDCA	15	NR	NR	Mean CfB: −0.2 (SD=3.8)
	NCT01904058	Week 13	LUM001 10 mg + UDCA (Cohort A)	18	NR	NR	Mean CfB: −7.4 (SD=3.68)
			LUM001 20 mg + UDCA (Cohort B)	21	NR	NR	% CfB: −7 (SD=5.89)
			Placebo + UDCA (Cohort A)	10	NR	NR	% CfB: −7.8 (SD=5.94)
			Placebo + UDCA (Cohort B)	12	NR	NR	% CfB: −6.1 (SD=4.68)
		Week 13/early termination	LUM001 10 mg + UDCA (Cohort A)	21	NR	NR	% CfB: −6.5 (SD=4.11)
			LUM001 20 mg + UDCA (Cohort B)	21	NR	NR	% CfB: −7 (SD=5.89)
			Placebo (Cohort A)	10	NR	NR	% CfB: −7.8 (SD=5.94)
			Placebo (Cohort B)	13	NR	NR	% CfB: −5.6 (SD=4.79)
	NCT02955602	Week 52	Seladelpar 5 mg	49	NR	NR	NR
			Seladelpar 10 mg	52	NR	NR	NR
	NCT03394924	Week 12	EDP-305 1 mg	24	NR	NR	LS Mean CfB: −0.95
			EDP-305 2.5 mg	21	NR	NR	LS Mean CfB: 3.19
			Placebo	8	NR	NR	LS Mean CfB: −3.16
	POISE (NCT01473524)	Month 12	OCA 10 mg	73	NR	NR	NR
			OCA 5–10 mg	70	NR	NR	NR
			Placebo	73	NR	NR	NR
PGIS	TRANSFORM (NCT05014672)	Week 24	Setanaxib 1200 mg	14	NR	NR	Mean CfB: −0.50 (SD=0.94)
			Setanaxib 1600 mg	16	NR	NR	Mean CfB: 0.25 (SD=0.78)
			Placebo	18	NR		Mean CfB: −0.11 (SD=0.76)
				15	NR	NR	Mean CfB: 2.73 (SD=1.75)
				21	NR	NR	Mean CfB: 3.38 (SD=1.24)
				19	NR	NR	Mean CfB: 3.37 (SD=1.54)

*Note:* Values presented as means, unless otherwise specified.

^a^ Median values reported.

^b^ All patients who underwent randomization and received ≥1 dose.

^c^ All patients who underwent randomization and received ≥1 dose, patients with a baseline pruritus NRS score ≥4.

Abbreviations: 5D, 5-dimensional; EOW, every other week; LS, least squares; NR, not reported; NRS, numerical rating score; OCA obeticholic acid; PBC, primary biliary cholangitis; PGIC, Patient Global Impression of Change; PGIS, Patient Global Impression of Severity; SE, standard error; UDCA, ursodeoxycholic acid; VAS, visual analog scale; WI, worst itch.

Multiple pruritus measures were used in 15 studies (34.1%), highlighting that different assessments of pruritus may be considered together when evaluating any potential treatment effect.

### Fatigue

Fatigue PROs were reported in 14 (31.8%) studies. Of these, 12 studies (27.3%) reported fatigue according to the PBC-40 Fatigue domain (Table 8), with changes from baseline reported in 9 studies (20.5%). In addition to the PBC-40 Fatigue domain, one study reported fatigue by the Fisk Fatigue Severity Scale (FFSS),^59^ one reported Patient-Reported Outcomes Measurement Information System (PROMIS) Short Form Fatigue, PGIS-Fatigue and PGIC-Fatigue,^66^ and another study scored daily fatigue levels on a 5-point scale, summed over 5 days.^54^

**TABLE 8:** Change from baseline in fatigue, assessed by the PBC-40 fatigue domain

Study name	Timepoint	Treatment arm	Denominator	Baseline value	Value at timepoint	Change from baseline, mean unless otherwise specified	Change from baseline, %	Change from baseline, LS mean	*p* vs. baseline	Treatment vs. comparator effect, mean difference	Treatment vs. comparator effect, LS mean difference	*p* vs. comparator
Bowlus, et al^56^ (NCT02955602)	Week 52	Seladelpar 2 mg	10	NR	NR	−1.2 (SD=7.8)	NR	NR	NR	NR	NR	NR
		Seladelpar 5 mg	42	NR	NR	−3 (SD=8.5)	NR	NR	NR	NR	NR	NR		
		Seladelpar 10 mg	48	NR	NR	−3.4 (SD=6.3)	NR	NR	NR	NR	NR	NR
ELATIVE (NCT04526665)	Week 52	Placebo	16	NR	NR	NR	NR	−1.0	NR	NR	NR	NR
		Elafibranor 80 mg	42	NR	NR	NR	NR	−2.2	NR	NR	−1.2 (vs. placebo)	NR
GLIMMER (NCT02966834)	Day 1	All patients	NR	NR	34 (SD=10.68); median=36	NR	NR	NR	NR	NR	NR	NR
HAART (NCT01614405)	Month 6	TDC/FTC/LPRr + UDCA	5	33^a^	37.5^a^	NR	NR	NR	0.285	NR	NR	NR
		Placebo + UDCA	6	31.5^a^	33^a^	NR	NR	NR	0.833	NR	NR	NR		
Hegade, et al^57^ (NCT01899703)	Day 14	GSK2330672 sequence 1	11	3.25 (SD=0.329)	3.06 (SE=0.329)	−7 (SE=5)	NR	NR	NR	NR	NR	NR
		Placebo sequence 1	11	3.25 (SD=0.329)	3.17 (SE=0.329)	−4 (SE=5)	NR	NR	NR	NR	NR	NR		
		Placebo sequence 2	10	3.34 (SD=0.329)	3.04 (SE=0.331)	−10 (SE=5)	NR	NR	NR	NR	NR	NR
		GSK2330672 sequence 2	10	3.34 (SD=0.329)	2.46 (SE=0.331)	−26 (SE=5)	NR	NR	NR	NR	NR	NR		
Hirschfield, et al^40^	Month 36	Budesonide + UDCA	40	26.8 (SD=11.8)	NR	−1.6 (SD=7.1)	NR	NR	NR	NR	NR	NR
		Placebo + UDCA	22	32.8 (SD=13.3)	NR	−0.4 (SD=9.3)	NR	NR	NR	NR	NR	NR		
Mayo, et al^49^ (NCT02026401)	Day 28	Aldafermin (NGM282) 0.3 mg + UDCA	14	NR	NR	−4.6 (SD=10.3)	NR	NR	NR	NR	NR	0.67 (vs. placebo)
		Aldafermin (NGM282) 3 mg + UDCA	13	NR	NR	−1.9 (SD=4.3)	NR	NR	NR	NR	NR	0.8 (vs. placebo)		
		Placebo + UDCA	15	NR	NR	−3.1 (SD=6.2)	NR	NR	NR	NR	NR	NR
NCT03226067	Week 24	GKT137831 400 mg QD	37	26.7 (SE=1.7)	26.1 (SD=1.7)	−0.8 (SE=1.0)	0.3 (SD=24.9)	NR	NR	NR	NR	NR
		GKT137831 400 mg BID	30	29.5 (SE=1.9)	25.2 (SD=1.8)	−3.6 (SE=1.3)	−9.9 (SD=19.8)	NR	NR	−12.2	NR	0.027 (vs. placebo)
		Placebo	36	28.4 (SE=2.0)	29.2 (SD=2.2)	0.6 (SE=0.9)	2.4 (SD=23.1)	NR	NR	NR	NR	NR		
NCT03394924	Week 12	EDP-305 1 mg	28	NR	NR	−0.36	NR	NR	NR	NR	NR	0.839 (vs. placebo)
		EDP-305 2.5 mg	21	NR	NR	−0.22	NR	NR	NR	NR	NR	0.891 (vs. placebo)		
		Placebo	9	NR	NR	0.1	NR	NR	NR	NR	NR	NR
Silveira, et al^59^	Week 12	Modafinil 100 mg	17	NR	28^a^	−6^a^	NR	NR	0.19	NR	NR	0.49 (vs. placebo)
		Placebo	19	NR	30.5^a^	−2^a^	NR	NR	NR	NR	NR	NR
Tanaka, et al^58^ (NCT03092765)	Week 12	E6011 10 mg/kg/mo	4	NR	NR	2.8 (SD=3.0)	NR	NR	NR	NR	NR	NR
		E6011 15 mg/kg/mo	4	NR	NR	−1.3 (SD=2.4)	NR	NR	NR	NR	NR	NR
		E6011 10 mg/kg/EOW	14	NR	NR	−1.6 (SD=5.9)	NR	NR	NR	NR	NR	NR		
		Placebo	7	NR	NR	−1.3 (SD=6.4)	NR	NR	NR	NR	NR	NR
TRANSFORM (NCT05014627)	Week 24	Setanaxib 1200 mg	24	NR	NR	−1.3 (SD=6.4)	NR	−1.44	0.42	NR	0.42	0.57
		Setanaxib 1200 mg	23	NR	NR	2.8 (SD=3.0)	NR	−1.83	0.02	NR	0.02	0.50
		Setanaxib combined doses	47	NR	NR	−1.6 (SD=5.9)	NR	1.64	0.21	NR	0.21	0.54		
		Placebo	25	NR	NR	−1.3 (SD=2.4)	NR	−1.85	NR	NR		NR

^a^ Median values reported.

Abbreviations: EOW, every other week; LS, least squares; NR, not reported; OCA, obeticholic acid; SD, standard deviation; SE, standard error; UDCA, ursodeoxycholic acid.

### Long-term outcomes

Event-free survival (EFS) was reported in 1 (2.3) study, and mortality was reported in 18 studies (40.9%). Overall survival (OS) was reported in 2 studies (4.5%), and transplant-free survival (TFS) was reported in 3 studies each (2.3%).^37^

### Safety outcomes

The incidence of adverse events (AEs) and serious AEs were reported in 23 (52.3%) and 25 (56.8%) studies, respectively. Treatment discontinuation due to AEs was reported in 22 studies (50.0%), and severe AEs were reported in 9 studies (20.5%). While safety outcomes may vary by drug class, gastrointestinal AEs were most commonly reported across all interventions, particularly diarrhea [n=23 studies (52.8%)], nausea [22 studies (50.0%)], and abdominal pain in 22 studies (50.0%). Neurological AEs of headache [n=23 (52.3%)], fatigue [n=21 (47.7%)], and dizziness [n=15 (34.9%)], as well as dermatological AEs of pruritus [n=21 (47.7%)] and rash [n=10 (22.7%)] were also commonly reported. Fractures were reported in 6 studies (13.6%).

Hepatic AEs of autoimmune hepatitis, hepatic cirrhosis, hepatic injury, hepatic failure, abnormal hepatic function, and hepatic encephalopathy were listed in 1 study (2.3%) each. Renal AEs, including acute kidney injury and renal impairment/dysfunction, were listed in 2 studies each (4.8%). Elevated transaminases were listed in 2 studies (4.5%), and hepatic enzyme increases were listed in 4 studies (9.1%). However, AEs of special interest data were not extracted in full.

### Quality assessment

A summary of the quality assessment conducted for each study is presented in Supplemental Table S14, https://links.lww.com/HC9/C483. Appropriate randomization was reported in 22 studies (50.0%), and 5 additional studies (11.4%) reported randomization with no further details. Treatment allocation concealment was reported in 20 studies (45.5%), and blinding of outcome assessors was reported in 28 studies (63.6%).

## DISCUSSION

This SLR captured a large body of relevant RCT data (149 publications reporting 44 unique studies) on the PBC treatment landscape in the 2L/2L+ setting, which provided a comprehensive evidence base for this review.

Several key biochemical markers of cholestasis were commonly reported across studies, including ALP, TB, and GGT. ALP is an integral component of various composite response criteria, and changes in ALP alone (presented as absolute change over time or relative changes from baseline), as well as ALP normalization, were often used as key endpoints. This reporting is in line with a shifting paradigm from using reduction to set multiples of ULN values (eg, 1.67×ULN) towards normalization as a treatment target. Assessment of biochemical markers varied across studies, with differences in assays used and definitions of ULN (Supplemental Table S13, https://links.lww.com/HC9/C483). Reporting of each biochemical marker also differed, with some studies reporting baseline and endpoint values, and others reporting percentage change or categorical changes from baseline, or normalization. In addition, baseline biochemistries varied markedly across study populations. As treatment response endpoints are often defined by reaching a set threshold of biochemical markers, higher baseline values and lower ULN values yield a lower likelihood of achieving a response because the relative reduction required to reach the threshold for response is larger.^61^ This effect is also evidenced by the variability in placebo responses observed in the RCTs captured here, highlighting the impact that differing baseline values and definitions of response may have on the interpretation of clinical data. Altogether, this variability presents challenges in evaluating the biochemical efficacy of different treatments in PBC, particularly given the lack of head-to-head trials for treatments in PBC.

Assessment of liver fibrosis and cirrhosis, alongside cholestatic and liver function markers, is useful to understand the potential impact of individual treatments on disease progression; NITs for fibrosis can provide some indication of a treatment effect. ELF, LSM, and PRO-C3 were the most commonly used NITs across the included studies; however, they were reported in less than a third of RCTs (n=11). Notably, among the 7 studies that reported LSM, values were not available for all patients included in these studies due to the limitations of costs, laboratory equipment, capabilities, and availability of technologies required for LSM evaluation (eg, FibroScan). Given that the included studies were largely RCTs, marked changes in NITs would not be expected within their relatively short timeframes, and the effects of novel treatments on disease progression and clinical outcomes, measured by progression-free survival (PFS) and TFS, will be established in ongoing open-label studies and with real-world data in the long term.^67^,^68^,^69^

Alongside improvements in liver biochemistry and slowing disease progression as a treatment goal in PBC, improvement in symptom burden has increasingly become a key focus for newer therapies. Pruritus and fatigue were the most often evaluated symptoms, but were not consistently reported. In addition, the PRO measures used to evaluate pruritus and fatigue, as well as the approaches to reporting these data, varied across studies, highlighting differences across studies in the evaluation of symptom burden.

Reported AEs were commonly gastrointestinal in nature, in line with the pathology of PBC, and treatment discontinuations due to AEs were not frequent. Notably, patient populations differed across studies due to differences in study design, disease characteristics, and eligibility criteria. Furthermore, potential variations in how AEs were defined and reported, relatively small population sizes, and contraindications of some drugs in different patients may influence AE outcomes; therefore, safety profiles across treatments cannot be compared.

The variability in study designs, timepoints of assessment, baseline patient and disease characteristics, and response endpoints, as evidenced in this review, as well as the lack of head-to-head trials, highlights the fragmented evidence base of current treatments in PBC. Consequently, clinicians face challenges in applying clinical trial results to inform personalized treatment decisions for individual patients. This heterogeneity of endpoints for clinical, safety, and PRO outcomes across studies emphasizes the importance of efforts such as the EASL/AASLD joint program for Global Consensus PBC Endpoints, which outlines consensus recommendations on endpoints, study of symptoms, and use of real-world evidence to advance the development of reliable surrogate endpoints for RCTs in PBC.^70^,^71^

### Strengths and limitations

In this review, multiple electronic databases and supplementary reference lists of relevant SLRs and NMAs were searched following a systematic approach. Two reviewers performed each stage of study selection independently to minimize selection bias and ensure all relevant literature was captured and included. While the prioritization strategies implemented may have led to the exclusion of potentially relevant studies, they ensured that only the most pertinent evidence for the purpose of the review was included, given the high volume of literature available. The pre-defined protocol detailed the robust methodology employed here and enables systematic updating of the SLR in the future.

## CONCLUSION

This SLR provides a comprehensive evidence base of key clinical efficacy and safety data for 2L/2L+ treatments in PBC. Due to the heterogeneity in study designs, response endpoints, eligibility criteria, and reporting, as well as in patient populations, direct comparisons of the relative effectiveness and safety of these therapies are limited. Furthermore, while some validated disease-specific PRO tools exist, heterogeneity in the selection and application of different measurement scales across studies introduces substantial variation in treatment outcome reporting. In light of this fragmented evidence base, further research is needed to inform decision-making in the context of the current 2L/2L+ landscape in PBC.

## Supplementary Material
