# Supplementary material for: Clinical, safety, and patient-reported outcomes associated with second-line and later therapies in primary biliary cholangitis: A systematic review
Source: Hepatol Commun. 2026 Oct 14;10(11):e01048. doi: 10.1097/HC9.0000000000001048 (PMC13668302; doi:10.1097/HC9.0000000000001048)
Supplement: Supplementary file 1 [file hc9-10-e01048-s001.docx]

# Clinical, Safety, and Patient-Reported Outcomes Associated with Second-Line and Later Therapies in Primary Biliary Cholangitis: A Systematic Review

**Corresponding Author:** Marco Carbone

[**Supplementary Table 1.** Search terms used for MEDLINE (via Ovid SP) in original SLR and SLR Update I	2](#_Toc228530808)

[**Supplementary Table 2.** Search terms used for Embase (via Ovid SP) in original SLR and SLR Update I	4](#_Toc228530809)

[**Supplementary Table 3.** Search terms used in CDSR and CENTRAL (via the Wiley platform) in original SLR and SLR Update I	6](#_Toc228530810)

[**Supplementary Table 4.** Search terms used in the DARE (via the University of York CRD platform) in original SLR	7](#_Toc228530811)

[**Supplementary Table 5.** Search terms used in MEDLINE, Embase, CDSR and CENTRAL (via Ovid SP) in SLR Update II	8](#_Toc228530812)

[**Supplementary Table 6.** Search terms used in MEDLINE and Embase (via Ovid SP) in SLR Update III	11](#_Toc228530813)

[**Supplementary Table 7.** Search terms used in CDSR and CENTRAL (via Ovid SP) in SLR Update III	13](#_Toc228530814)

[**Supplementary Table 8**. Search terms used for congress websites in the SLR	15](#_Toc228530815)

[**Supplementary Table 9**. Search terms used for ClinicalTrials.gov	18](#_Toc228530816)

[**Supplementary Table 10.** Studies included and prioritized for extraction in the SLR	19](#_Toc228530817)

[**Supplementary Table 11.** Baseline patient characteristics	30](#_Toc228530818)

[**Supplementary Table 12.** Baseline treatment characteristics	40](#_Toc228530819)

[**Supplementary Table 13**. ULN Values Across Studies	47](#_Toc228530820)

[**Supplementary Table 14.** Quality assessment	48](#_Toc228530821)

**Supplementary Table 1.** Search terms used for MEDLINE (via Ovid SP) in original SLR and SLR Update I

| Term group | # | Searches | Results  11.21.2022 | Results 12.18.2023 |
| --- | --- | --- | --- | --- |
| Disease area: primary biliary cholangitis | 1 | *Liver Cirrhosis, Biliary/ | 6690 | 6895 |
|  | 2 | (primary biliary cholangitis or primary biliary cholestasis or primary biliary cirrhosis or PBC).ti,ab,kf. | 11323 | 11882 |
|  | 3 | 1 or 2 | 12480 | 13050 |
| Interventional studies (RCTs and non-RCTs) | 4 | Randomized Controlled Trials as Topic/ | 158804 | 165474 |
|  | 5 | Randomized Controlled Trial/ | 581137 | 604215 |
|  | 6 | Random Allocation/ | 106894 | 107041 |
|  | 7 | Double-Blind Method/ | 173639 | 176795 |
|  | 8 | Single-Blind Method/ | 32303 | 33081 |
|  | 9 | Placebos/ | 35924 | 35935 |
|  | 10 | exp Clinical Trials as topic/ | 378425 | 386313 |
|  | 11 | Clinical Trial/ | 536481 | 539133 |
|  | 12 | Clinical Trial, Phase I/ or Clinical Trial, Phase II/ or Clinical Trial, Phase III/ or Clinical Trial, Phase IV/ | 78653 | 82298 |
|  | 13 | Controlled Clinical Trial/ or Adaptive Clinical Trial/ | 95139 | 95510 |
|  | 14 | randomized controlled trial.pt. | 581137 | 604215 |
|  | 15 | clinical trial.pt. | 536481 | 539133 |
|  | 16 | (clinical trial, phase i or clinical trial, phase ii or clinical trial, phase iii or clinical trial, phase iv).pt. | 78653 | 82298 |
|  | 17 | (controlled clinical trial or multicenter study).pt. | 419005 | 432168 |
|  | 18 | (clinical adj trial$).ti,ab,kf. | 466974 | 508526 |
|  | 19 | ((singl$ or doubl$ or treb$ or tripl$) adj (blind$3 or mask$3)).ti,ab,kf. | 192862 | 201304 |
|  | 20 | placebo$.ti,ab,kf. | 241949 | 252567 |
|  | 21 | (allocat$ adj2 random$).ti,ab,kf. | 42013 | 45098 |
|  | 22 | Randomi?ed adj2 trial$.ti,ab,kf. | 407080 | 446042 |
|  | 23 | rct.ti,ab,kf. | 31749 | 35777 |
|  | 24 | (single arm adj3 (trial$ or stud$)).ti,ab,kf. | 8388 | 9945 |
|  | 25 | (open label adj (trial$ or stud$)).ti,ab,kf. | 13048 | 13768 |
|  | 26 | (non blinded adj (trial$ or stud$)).ti,ab,kf. | 224 | 243 |
|  | 27 | (pragmatic trial$ or pragmatic stud$).ti,ab,kf. or Pragmatic Clinical Trial/ | 4246 | 4664 |
|  | 28 | or/4-27 | 1983660 | 2079478 |
| Observational studies | 29 | Epidemiologic Studies/ | 9193 | 9443 |
|  | 30 | Observational Study/ | 134780 | 149384 |
|  | 31 | Cohort Studies/ | 321739 | 335173 |
|  | 32 | exp Case-Control Studies/ | 1369979 | 1462987 |
|  | 33 | Cross-Sectional Studies/ | 446870 | 485289 |
|  | 34 | Clinical Study/ | 5331 | 5716 |
|  | 35 | Follow-Up Studies/ | 688450 | 694423 |
|  | 36 | Longitudinal Studies/ | 161468 | 168223 |
|  | 37 | Retrospective Studies/ | 1074257 | 1162360 |
|  | 38 | Prospective Studies/ not Randomized Controlled Trials as Topic/ | 636789 | 666225 |
|  | 39 | (observational adj (study or studies)).ti,ab,kf. | 150834 | 171221 |
|  | 40 | (cohort adj (study or studies)).ti,ab,kf. | 296874 | 338391 |
|  | 41 | cohort analy$.ti,ab,kf. | 11797 | 13284 |
|  | 42 | case control.ti,ab,kf. | 149419 | 159415 |
|  | 43 | cross sectional.ti,ab,kf. | 479274 | 538561 |
|  | 44 | (follow up adj (study or studies)).ti,ab,kf. | 56705 | 59248 |
|  | 45 | longitudinal.ti,ab,kf. | 310639 | 339180 |
|  | 46 | retrospective.ti,ab,kf. | 700115 | 778500 |
|  | 47 | (chart adj3 review$).ti,ab,kf. | 52057 | 55944 |
|  | 48 | exp Registries/ | 115210 | 118720 |
|  | 49 | (registry or registries).ti,ab,kf. | 167044 | 184293 |
|  | 50 | (prospective adj (study or studies)).ti,ab,kf. | 202477 | 214167 |
|  | 51 | (epidemiologic$ adj (study or studies)).ti,ab,kf. | 93730 | 97592 |
|  | 52 | (evaluation adj (study or studies)).ti,ab,kf. | 7144 | 7723 |
|  | 53 | (chart adj3 review$).ti,ab,kf. | 52057 | 55944 |
|  | 54 | or/29-53 | 3900849 | 4164577 |
| Exclusion terms | 55 | exp animals/ not exp humans/ | 5067266 | 5175456 |
|  | 56 | (comment or editorial or case reports or historical article).pt. | 4052655 | 4197494 |
|  | 57 | (case stud$ or case report$).ti. | 362167 | 395457 |
|  | 58 | or/55-57 | 9111076 | 9368540 |
| Combined | 59 | 3 and (28 or 54) | 2969 | 3144 |
|  | 60 | 59 not 58 | 2814 | 2981 |
| **Date limit** | 61 | limit 60 to yr=2022-current | **-** | 328 |

Ovid MEDLINE(R) and Epub Ahead of Print, In-Process, In-Data-Review & Other Non-Indexed Citations and Daily 1946 to November 21, 2022 (original SLR) to December 18, 2023 (SLR Update I).

PBC: primary biliary cholangitis; RCT: randomized controlled trial; SLR: systematic literature review.

**Supplementary Table 2.** Search terms used for Embase (via Ovid SP) in original SLR and SLR Update I

| **Term group** | **#** | **Searches** | **Results 11.21.2022** | **Results 12.18.2023** |
| --- | --- | --- | --- | --- |
| **Disease area: primary biliary cholangitis** | 1 | *biliary cirrhosis/ or *primary biliary cirrhosis/ | 8979 | 9426 |
|  | 2 | (primary biliary cholangitis or primary biliary cholestasis or primary biliary cirrhosis or PBC).ti,ab,kf. | 16850 | 17930 |
|  | 3 | 1 or 2 | 17853 | 18947 |
| **Interventional studies (RCTs and non-RCTs)** | 4 | "randomized controlled trial (topic)"/ | 238877 | 266139 |
|  | 5 | randomized controlled trial/ | 737030 | 795567 |
|  | 6 | randomization/ | 95509 | 98914 |
|  | 7 | double blind procedure/ | 200828 | 213246 |
|  | 8 | single blind procedure/ | 48290 | 52660 |
|  | 9 | crossover procedure/ | 72089 | 76088 |
|  | 10 | placebo/ | 387937 | 405856 |
|  | 11 | exp "clinical trial (topic)"/ | 409495 | 450701 |
|  | 12 | clinical trial/ | 1048666 | 1076786 |
|  | 13 | phase 1 clinical trial/ or phase 2 clinical trial/ or phase 3 clinical trial/ or phase 4 clinical trial/ | 205835 | 225449 |
|  | 14 | controlled clinical trial/ or adaptive clinical trial/ or multicenter study/ | 747426 | 787967 |
|  | 15 | (clinical adj trial$).ti,ab,kf. | 674319 | 735953 |
|  | 16 | ((singl$ or doubl$ or treb$ or tripl$) adj (blind$3 or mask$3)).ti,ab,kf. | 270418 | 284726 |
|  | 17 | placebo$.ti,ab,kf. | 352307 | 372069 |
|  | 18 | (allocat$ adj2 random$).ti,ab,kf. | 51998 | 55892 |
|  | 19 | Randomi?ed adj2 trial$.ti,ab,kf. | 547249 | 598498 |
|  | 20 | rct.ti,ab,kf. | 52363 | 58560 |
|  | 21 | (single arm adj3 (trial$ or stud$)).ti,ab,kf. | 17498 | 20292 |
|  | 22 | (open label adj (trial$ or stud$)).ti,ab,kf. | 23301 | 24853 |
|  | 23 | (non blinded adj (trial$ or stud$)).ti,ab,kf. | 335 | 351 |
|  | 24 | (pragmatic trial$ or pragmatic stud$).ti,ab,kf. or pragmatic trial/ | 4272 | 5093 |
|  | 25 | or/4-24 | 2812930 | 3007300 |
| **Observational studies** | 26 | observational study/ | 296009 | 347384 |
|  | 27 | cohort analysis/ | 920982 | 1082815 |
|  | 28 | exp case control study/ | 213641 | 228503 |
|  | 29 | cross-sectional study/ | 517701 | 597044 |
|  | 30 | clinical study/ | 161049 | 164846 |
|  | 31 | follow up/ | 1924442 | 2109731 |
|  | 32 | longitudinal study/ | 181456 | 201480 |
|  | 33 | retrospective study/ | 1339939 | 1527998 |
|  | 34 | prospective study/ not "randomized controlled trial (topic)"/ | 799840 | 884329 |
|  | 35 | (observational adj (study or studies)).ti,ab,kf. | 233236 | 264920 |
|  | 36 | (cohort adj (study or studies)).ti,ab,kf. | 430095 | 488963 |
|  | 37 | cohort analy$.ti,ab,kf. | 17921 | 20283 |
|  | 38 | case control.ti,ab,kf. | 196954 | 211008 |
|  | 39 | cross sectional.ti,ab,kf. | 624534 | 702065 |
|  | 40 | (follow up adj (study or studies)).ti,ab,kf. | 74657 | 78626 |
|  | 41 | longitudinal.ti,ab,kf. | 419162 | 459450 |
|  | 42 | retrospective.ti,ab,kf. | 1162214 | 1290712 |
|  | 43 | (chart adj3 review$).ti,ab,kf. | 109806 | 119049 |
|  | 44 | exp disease registry/ | 63100 | 69043 |
|  | 45 | (registry or registries).ti,ab,kf. | 277542 | 305527 |
|  | 46 | (prospective adj (study or studies)).ti,ab,kf. | 307815 | 328891 |
|  | 47 | (epidemiologic$ adj (study or studies)).ti,ab,kf. | 121243 | 126785 |
|  | 48 | (evaluation adj (study or studies)).ti,ab,kf. | 9861 | 10559 |
|  | 49 | (chart adj3 review$).ti,ab,kf. | 109806 | 119049 |
|  | 50 | or/26-49 | 5793326 | 6367060 |
| **Exclusion terms** | 51 | ("conference abstract" or "conference review").pt. | 4607453 | 4992469 |
|  | 52 | limit 51 to yr="1974-2020" | 4132436 | 4165404 |
|  | 53 | exp animal/ not exp human/ | 5032838 | 5176772 |
|  | 54 | editorial.pt. | 743715 | 787672 |
|  | 55 | editorial/ or case report/ | 3510721 | 3694639 |
|  | 56 | (case stud$ or case report$).ti. | 439545 | 478574 |
|  | 57 | or/52-56 | 12150738 | 12512631 |
| **Combined** | 58 | 3 and (25 or 50) | 5634 | 6195 |
|  | 59 | 58 not 57 | 3236 | 3739 |
| **Date limit** | 60 | limit 59 to yr=2022-current | - | 831 |

Embase 1974 to November 11, 2022 (original SLR), Embase 2022 to December 18, 2023 (SLR Update I).

PBC: primary biliary cholangitis; RCT: randomized controlled trial; SLR: systematic literature review.

**Supplementary Table 3.** Search terms used in CDSR and CENTRAL (via the Wiley platform) in original SLR and SLR Update I

| Term group | # | Searches | Results  11.21.2022 | Results 12.18.2023 |
| --- | --- | --- | --- | --- |
| Disease area: primary biliary cholangitis | 1 | [mh “Liver Cirrhosis, Biliary”] | 336 | 372 |
|  | 2 | (primary biliary cholangitis or primary biliary cholestasis or primary biliary cirrhosis or PBC):ti,ab,kw | 1630 | 1825 |
|  | 3 | #1 or #2 | 1645 | 1841 |
| Limits (original SLR) | 4 | #3 in Cochrane Reviews and Cochrane Protocols | 59 | - |
|  | 5 | #3 in Trials | 1584 | - |
| **Limits (SLR Update I)** | 6 | #3 in with Cochrane Library publication date from Nov 2022 to Dec 2023, in Cochrane Reviews and Cochrane Protocols | - | 1 |
|  | 7 | #3 with Publication Year from 2022 to 2023, in Trials | - | 187 |

Cochrane Central Register of Controlled Trials Issue 10 of 12, October 2022 (original SLR), December 2023 (SLR Update I); Cochrane Database of Systematic Reviews Issue 11 of November 12, 2022 (original SLR), Issue 10 of December 18, 2023 (SLR Update I).

CDSR: Cochrane Database of Systematic Reviews; CENTRAL: Cochrane Central Register of Controlled Trials; PBC: primary biliary cholangitis; SLR, systematic literature review.

**Supplementary Table 4.** Search terms used in the DARE (via the University of York CRD platform) in original SLR

| Term group | # | Searches | Results  11.21.2022 |
| --- | --- | --- | --- |
| Disease area: primary biliary cholangitis | 1 | MeSH DESCRIPTOR Liver Cirrhosis, Biliary | 17 |
|  | 2 | "primary biliary cholangitis" or "primary biliary cholestasis" or "primary biliary cirrhosis" or PBC | 54 |
|  | 3 | #1 OR #2 | 57 |
| Total | 4 | #3 in DARE | 37 |

Database of Abstracts of Reviews of Effect: Issue 2 of April 4, 2015 (original SLR). DARE was not searched for the updates as this database was last updated in 2016.

DARE: Database of Abstracts of Reviews of Effect; CRD: Centre for Reviews and Dissemination; PBC: primary biliary cholangitis; SLR: systematic literature review.

**Supplementary Table 5.** Search terms used in MEDLINE, Embase, CDSR and CENTRAL (via Ovid SP) in SLR Update II

| **Term Group** | **#** | **Searches** | **Results**  **06.19.2024** |
| --- | --- | --- | --- |
| **Disease area: primary biliary cholangitis** | 1 | *Liver Cirrhosis, Biliary/ or *biliary liver cirrhosis/ or "liver cirrhosis, biliary".kw. | 9462 |
|  | 2 | (primary biliary cholangitis or primary biliary cholestasis or primary biliary cirrhosis or PBC).ti,ab,kf,kw. | 32139 |
|  | 3 | 1 or 2 | 34060 |
| **Interventional studies (RCTs and non-RCTs)** | 4 | Randomized Controlled Trials as Topic/ or "randomized controlled trial (topic)"/ | 492407 |
|  | 5 | Randomized Controlled Trial/ | 1443716 |
|  | 6 | Random Allocation/ or randomization/ | 232820 |
|  | 7 | Double-Blind Method/ or double blind procedure/ | 570342 |
|  | 8 | Single-Blind Method/ or single blind procedure/ | 116158 |
|  | 9 | crossover procedure/ | 78461 |
|  | 10 | Placebos.mp. or placebo/ [mp=ti, bt, ab, ot, nm, hw, fx, kf, ox, px, rx, ui, sy, ux, mx, tn, dm, mf, dv, dq] | 483508 |
|  | 11 | exp Clinical Trials as Topic/ or exp "clinical trial (topic)"/ | 949383 |
|  | 12 | Clinical Trial/ | 1624297 |
|  | 13 | Clinical Trial, Phase I/ or phase 1 clinical trial/ or Clinical Trial, Phase II/ or phase 2 clinical trial/ or Clinical Trial, Phase III/ or phase 3 clinical trial/ or Clinical Trial, Phase IV/ or phase 4 clinical trial/ | 329011 |
|  | 14 | Controlled Clinical Trial/ or Adaptive Clinical Trial/ or multicenter study/ | 1245872 |
|  | 15 | randomized controlled trial.pt. | 615985 |
|  | 16 | clinical trial.pt. | 540075 |
|  | 17 | (clinical trial, phase i or clinical trial, phase ii or clinical trial, phase iii or clinical trial, phase iv).pt. | 84440 |
|  | 18 | (controlled clinical trial or multicenter study).pt. | 440867 |
|  | 19 | (clinical adj trial$).ti,ab,kf,kw. | 1764490 |
|  | 20 | ((singl$ or doubl$ or treb$ or tripl$) adj (blind$3 or mask$3)).ti,ab,kf. | 837229 |
|  | 21 | placebo$.ti,ab,kf. | 1017128 |
|  | 22 | (allocat$ adj2 random$).ti,ab,kf. | 164469 |
|  | 23 | (Randomi?ed adj2 trial$).ti,ab,kf. | 1550790 |
|  | 24 | rct.ti,ab,kf. | 139674 |
|  | 25 | (single arm adj3 (trial$ or stud$)).ti,ab,kf. | 36031 |
|  | 26 | (open label adj (trial$ or stud$)).ti,ab,kf. | 52999 |
|  | 27 | (non blinded adj (trial$ or stud$)).ti,ab,kf. | 905 |
|  | 28 | (pragmatic trial$ or pragmatic stud$).ti,ab,kf. or Pragmatic Clinical Trial/ or pragmatic trial/ | 13792 |
|  | 29 | or/4-28 | 6384853 |
| **Observational studies** | 30 | Epidemiologic Studies/ or epidemiology/ | 287693 |
|  | 31 | Observational Study/ | 536145 |
|  | 32 | Cohort Studies/ or cohort analysis/ | 1535181 |
|  | 33 | exp Case-Control Studies/ or exp case control study/ | 1781873 |
|  | 34 | Cross-Sectional Studies/ or cross-sectional study/ | 1158819 |
|  | 35 | Clinical Study/ | 173154 |
|  | 36 | Follow-Up Studies/ or follow up/ | 2979440 |
|  | 37 | Longitudinal Studies/ or longitudinal study/ | 397166 |
|  | 38 | Retrospective Studies/ or retrospective study/ | 2868742 |
|  | 39 | (Prospective Studies/ not Randomized Controlled Trials as Topic/) or (prospective study/ not "randomized controlled trial (topic)"/) | 1731907 |
|  | 40 | (observational adj (study or studies)).ti,ab,kf. | 475042 |
|  | 41 | (cohort adj (study or studies)).ti,ab,kf. | 896053 |
|  | 42 | cohort analy$.ti,ab,kf. | 36355 |
|  | 43 | case control.ti,ab,kf. | 387764 |
|  | 44 | cross sectional.ti,ab,kf. | 1327583 |
|  | 45 | (follow up adj (study or studies)).ti,ab,kf. | 147849 |
|  | 46 | longitudinal.ti,ab,kf. | 855908 |
|  | 47 | retrospective.ti,ab,kf. | 2209603 |
|  | 48 | (prospective adj (study or studies)).ti,ab,kf. | 591632 |
|  | 49 | (epidemiologic$ adj (study or studies)).ti,ab,kf. | 233408 |
|  | 50 | (evaluation adj (study or studies)).ti,ab,kf. | 20139 |
|  | 51 | (chart adj3 review$).ti,ab,kf. | 184930 |
|  | 52 | Or/30-51 | 11206342 |
| **Exclusion terms** | 53 | conference$.pt. | 6215960 |
|  | 54 | limit 53 to yr="1974-2022" | 5790426 |
|  | 55 | exp animals/ not exp humans/ | 10506517 |
|  | 56 | (comment or editorial or case reports or historical article).pt. | 5082068 |
|  | 57 | editorial/ or case report/ | 4476605 |
|  | 58 | (case stud$ or case report$).ti. | 913547 |
|  | 59 | or/54-58 | 23610255 |
| **Combined** | 60 | 3 and (29 or 52) | 10653 |
|  | 61 | 60 not 59 | 7334 |
|  | 62 | 3 use coch | 18 |
|  | 63 | 3 use cctz | 1298 |
|  | 64 | 63 not 59 | 990 |
|  | 65 | Or/61,62,64 | 7696 |
|  | 66 | limit 65 to yr="2023-current" | 1108 |
|  | 67 | remove duplicates from 66 | 799 |

Cochrane Central Register of Controlled Trials May 2024, EBM Reviews - Cochrane Database of Systematic Reviews 2005 to June 12^th^, 2024, Embase 1974 to June 18, 2024, Ovid MEDLINE(R) Epub Ahead of Print and In-Process, In-Data-Review & Other Non-Indexed Citations and Daily June 18, 2024.

PBC: primary biliary cholangitis; RCT: randomized controlled trial; SLR: systematic literature review.

**Supplementary Table 6.** Search terms used in MEDLINE and Embase (via Ovid SP) in SLR Update III

| Term Group | # | Searches | Results  08.07.2025 |
| --- | --- | --- | --- |
| **Disease area: primary biliary cholangitis** | 1 | *Liver Cirrhosis, Biliary/ or *biliary liver cirrhosis/ | 9888 |
|  | 2 | (primary biliary cholangitis or primary biliary cholestasis or primary biliary cirrhosis or PBC).ti,ab,kf. | 33007 |
|  | 3 | or/1-2 | 34935 |
| **Interventional studies (RCTs and non-RCTs)** | 4 | Randomized Controlled Trials as Topic/ or "randomized controlled trial (topic)"/ | 483226 |
|  | 5 | Randomized Controlled Trial/ | 1735540 |
|  | 6 | Random Allocation/ or randomization/ | 209851 |
|  | 7 | Double-Blind Method/ or double blind procedure/ | 492048 |
|  | 8 | Single-Blind Method/ or single blind procedure/ | 116939 |
|  | 9 | crossover procedure/ | 103994 |
|  | 10 | Placebos.mp. or placebo/ [mp=ti, bt, ab, ot, nm, hw, fx, kf, ox, px, rx, ui, sy, ux, mx, tn, dm, mf, dv, dq] | 541300 |
|  | 11 | exp Clinical Trials as Topic/ or exp "clinical trial (topic)"/ | 907889 |
|  | 12 | Clinical Trial/ | 2173079 |
|  | 13 | Clinical Trial, Phase I/ or phase 1 clinical trial/ or Clinical Trial, Phase II/ or phase 2 clinical trial/ or Clinical Trial, Phase III/ or phase 3 clinical trial/ or Clinical Trial, Phase IV/ or phase 4 clinical trial/ | 471275 |
|  | 14 | Controlled Clinical Trial/ or Adaptive Clinical Trial/ or multicenter study/ | 1373661 |
|  | 15 | randomized controlled trial.pt. | 643371 |
|  | 16 | clinical trial.pt. | 1070955 |
|  | 17 | (clinical trial, phase i or clinical trial, phase ii or clinical trial, phase iii or clinical trial, phase iv).pt. | 90746 |
|  | 18 | (controlled clinical trial or multicenter study).pt. | 468749 |
|  | 19 | (clinical adj trial$).ti,ab,kf. | 1490716 |
|  | 20 | ((singl$ or doubl$ or treb$ or tripl$) adj (blind$3 or mask$3)).ti,ab,kf. | 593448 |
|  | 21 | placebo$.ti,ab,kf. | 745484 |
|  | 22 | (allocat$ adj2 random$).ti,ab,kf. | 120462 |
|  | 23 | (Randomi?ed adj2 trial$).ti,ab,kf. | 1279318 |
|  | 24 | rct.ti,ab,kf. | 122168 |
|  | 25 | (single arm adj3 (trial$ or stud$)).ti,ab,kf. | 48834 |
|  | 26 | (open label adj (trial$ or stud$)).ti,ab,kf. | 54561 |
|  | 27 | (non blinded adj (trial$ or stud$)).ti,ab,kf. | 804 |
|  | 28 | (pragmatic trial$ or pragmatic stud$).ti,ab,kf. or Pragmatic Clinical Trial/ or pragmatic trial/ | 13874 |
|  | 29 | or/4-28 | 6097680 |
| **Observational studies** | 30 | Epidemiologic Studies/ or epidemiology/ | 345498 |
|  | 31 | Observational Study/ | 658797 |
|  | 32 | Cohort Studies/ or cohort analysis/ | 1781230 |
|  | 33 | exp Case-Control Studies/ or exp case control study/ | 1884249 |
|  | 34 | Cross-Sectional Studies/ or cross-sectional study/ | 1304518 |
|  | 35 | Clinical Study/ | 177265 |
|  | 36 | Follow-Up Studies/ or follow up/ | 3205332 |
|  | 37 | Longitudinal Studies/ or longitudinal study/ | 438770 |
|  | 38 | Retrospective Studies/ or retrospective study/ | 3180065 |
|  | 39 | (Prospective Studies/ not Randomized Controlled Trials as Topic/) or (prospective study/ not "randomized controlled trial (topic)"/) | 1711456 |
|  | 40 | (observational adj (study or studies)).ti,ab,kf. | 548981 |
|  | 41 | (cohort adj (study or studies)).ti,ab,kf. | 1019832 |
|  | 42 | cohort analy$.ti,ab,kf. | 41382 |
|  | 43 | case control.ti,ab,kf. | 410798 |
|  | 44 | cross sectional.ti,ab,kf. | 1486056 |
|  | 45 | (follow up adj (study or studies)).ti,ab,kf. | 150371 |
|  | 46 | longitudinal.ti,ab,kf. | 925188 |
|  | 47 | retrospective.ti,ab,kf. | 2462769 |
|  | 48 | (prospective adj (study or studies)).ti,ab,kf. | 611868 |
|  | 49 | (epidemiologic$ adj (study or studies)).ti,ab,kf. | 240207 |
|  | 50 | (evaluation adj (study or studies)).ti,ab,kf. | 20867 |
|  | 51 | (chart adj3 review$).ti,ab,kf. | 198619 |
|  | 52 | or/30-51 | 12038825 |
| **Exclusion terms** | 53 | ("conference abstract" or "conference review").pt. | 5580780 |
|  | 54 | limit 53 to yr="1974-2022" | 4860919 |
|  | 55 | exp animals/ not exp humans/ | 11181523 |
|  | 56 | (comment or editorial or case reports or historical article).pt. | 5248190 |
|  | 57 | editorial/ or case report/ | 4689834 |
|  | 58 | (case stud$ or case report$).ti. | 995700 |
|  | 59 | or/54-58 | 23763551 |
| **Combined** | 60 | 3 and (29 or 52) | 10918 |
|  | 61 | 60 not 59 | 7885 |
|  | 62 | limit 61 to yr=2024-current | 1255 |

Ovid MEDLINE(R) Epub Ahead of Print and In-Process, In-Data-Review & Other Non-Indexed Citations and Daily August 5, 2025, Embase 1974 to 2025, August 5.

PBC: primary biliary cholangitis; RCT: randomized controlled trial; SLR: systematic literature review

**Supplementary Table 7.** Search terms used in CDSR and CENTRAL (via Ovid SP) in SLR Update III

| Term Group | # | Searches | Results  09.30.2025 |
| --- | --- | --- | --- |
| **Disease area: primary biliary cholangitis** | 1 | *Liver Cirrhosis, Biliary/ or *biliary liver cirrhosis/ | 19 |
|  | 2 | (primary biliary cholangitis or primary biliary cholestasis or primary biliary cirrhosis or PBC).ti,ab,kf. | 1413 |
|  | 3 | or/1-2 | 1413 |
| **Interventional studies (RCTs and non-RCTs)** | 4 | Randomized Controlled Trials as Topic/ or "randomized controlled trial (topic)"/ | 51688 |
|  | 5 | Randomized Controlled Trial/ | 34 |
|  | 6 | Random Allocation/ or randomization/ | 25204 |
|  | 7 | Double-Blind Method/ or double blind procedure/ | 169306 |
|  | 8 | Single-Blind Method/ or single blind procedure/ | 27589 |
|  | 9 | crossover procedure/ | 0 |
|  | 10 | Placebos.mp. or placebo/ [mp=ti, ot, ab, fx, sh, hw, kw, tx, ct] | 29581 |
|  | 11 | exp Clinical Trials as Topic/ or exp "clinical trial (topic)"/ | 95719 |
|  | 12 | Clinical Trial/ | 9 |
|  | 13 | Clinical Trial, Phase I/ or phase 1 clinical trial/ or Clinical Trial, Phase II/ or phase 2 clinical trial/ or Clinical Trial, Phase III/ or phase 3 clinical trial/ or Clinical Trial, Phase IV/ or phase 4 clinical trial/ | 446 |
|  | 14 | Controlled Clinical Trial/ or Adaptive Clinical Trial/ or multicenter study/ | 10 |
|  | 15 | randomized controlled trial.pt. | 0 |
|  | 16 | clinical trial.pt. | 0 |
|  | 17 | (clinical trial, phase i or clinical trial, phase ii or clinical trial, phase iii or clinical trial, phase iv).pt. | 0 |
|  | 18 | (controlled clinical trial or multicenter study).pt. | 0 |
|  | 19 | (clinical adj trial$).ti,ab,kf. | 270025 |
|  | 20 | ((singl$ or doubl$ or treb$ or tripl$) adj (blind$3 or mask$3)).ti,ab,kf. | 351947 |
|  | 21 | placebo$.ti,ab,kf. | 393013 |
|  | 22 | (allocat$ adj2 random$).ti,ab,kf. | 62448 |
|  | 23 | (Randomi?ed adj2 trial$).ti,ab,kf. | 490705 |
|  | 24 | rct.ti,ab,kf. | 44452 |
|  | 25 | (single arm adj3 (trial$ or stud$)).ti,ab,kf. | 3512 |
|  | 26 | (open label adj (trial$ or stud$)).ti,ab,kf. | 13912 |
|  | 27 | (non blinded adj (trial$ or stud$)).ti,ab,kf. | 303 |
|  | 28 | (pragmatic trial$ or pragmatic stud$).ti,ab,kf. or Pragmatic Clinical Trial/ or pragmatic trial/ | 3084 |
|  | 29 | or/4-28 | 1115468 |
| **Observational studies** | 30 | Epidemiologic Studies/ or epidemiology/ | 157 |
|  | 31 | Observational Study/ | 0 |
|  | 32 | Cohort Studies/ or cohort analysis/ | 12224 |
|  | 33 | exp Case-Control Studies/ or exp case control study/ | 29546 |
|  | 34 | Cross-Sectional Studies/ or cross-sectional study/ | 10068 |
|  | 35 | Clinical Study/ | 0 |
|  | 36 | Follow-Up Studies/ or follow up/ | 73681 |
|  | 37 | Longitudinal Studies/ or longitudinal study/ | 8939 |
|  | 38 | Retrospective Studies/ or retrospective study/ | 20979 |
|  | 39 | (Prospective Studies/ not Randomized Controlled Trials as Topic/) or (prospective study/ not "randomized controlled trial (topic)"/) | 127640 |
|  | 40 | (observational adj (study or studies)).ti,ab,kf. | 12201 |
|  | 41 | (cohort adj (study or studies)).ti,ab,kf. | 14051 |
|  | 42 | cohort analy$.ti,ab,kf. | 687 |
|  | 43 | case control.ti,ab,kf. | 5618 |
|  | 44 | cross sectional.ti,ab,kf. | 17435 |
|  | 45 | (follow up adj (study or studies)).ti,ab,kf. | 7110 |
|  | 46 | longitudinal.ti,ab,kf. | 23492 |
|  | 47 | retrospective.ti,ab,kf. | 26042 |
|  | 48 | (prospective adj (study or studies)).ti,ab,kf. | 32187 |
|  | 49 | (epidemiologic$ adj (study or studies)).ti,ab,kf. | 4489 |
|  | 50 | (evaluation adj (study or studies)).ti,ab,kf. | 1258 |
|  | 51 | (chart adj3 review$).ti,ab,kf. | 2754 |
|  | 52 | or/30-51 | 307229 |
| **Exclusion terms** | 53 | ("conference abstract" or "conference review").pt. | 0 |
|  | 54 | limit 53 to yr="1974-2022" | 0 |
|  | 55 | exp animals/ not exp humans/ | 1304 |
|  | 56 | (comment or editorial or case reports or historical article).pt. | 4655 |
|  | 57 | editorial/ or case report/ | 0 |
|  | 58 | (case stud$ or case report$).ti. | 1291 |
|  | 59 | or/54-58 | 7236 |
| **Combined** | 60 | 3 and (29 or 52) | 962 |
|  | 61 | 60 not 59 | 959 |
|  | 62 | limit 61 to yr=2024-current | 103 |

EBM Reviews - Cochrane Central Register of Controlled Trials August 2025, EBM Reviews - Cochrane Database of Systematic Reviews 2005 to September 24, 2025.

PBC: primary biliary cholangitis; RCT: randomized controlled trial; SLR: systematic literature review.

**Supplementary Table 8**. Search terms used for congress websites in the SLR

| **SLR** | **Congress** | **Link** | **Search strategy** | **Retrieved records** | **Included records** |
| --- | --- | --- | --- | --- | --- |
| **Original SLR** | **AASLD Liver Meeting 2021** | <https://aasldpubs.onlinelibrary.wiley.com/toc/15273350/2021/74/S1> | Oral and poster abstracts in the 'PBC/PSC and Other Cholestatic Disease' category of the online platform were screened for relevance | 72 | 2 |
|  | **AASLD Liver Meeting 2022** | <https://aasldpubs.onlinelibrary.wiley.com/toc/15273350/2022/76/S1> | Abstracts included in the abstract book under the 'PBC/PSC and Other Cholestatic Disease' category of the AASLD Liver Meeting 2022 Category Index were searched for and screened for relevance against the eligibility criteria | 59 | 5 |
|  | **EASL International Liver Congress 2021** | <https://easl.eu/wp-content/uploads/2021/06/EASL_2021_Version-5-new.pdf> | The abstract book was searched for the following terms using the advanced search function in Adobe Acrobat:  Primary biliary cholangitis  Primary biliary cirrhosis  PBC  All retrieved abstracts were screened for relevance | 184 | 4 |
|  | **EASL International Liver Congress 2022** | <https://easl.eu/wp-content/uploads/2022/06/EASL_2022_Version-4_Latest-version_compressed-1.pdf> | The abstract book was searched for the following terms using the advanced search function in Adobe Acrobat:  Primary biliary cholangitis  Primary biliary cirrhosis  PBC  All retrieved abstracts were screened for relevance | 324 | 1 |
|  | **EASL Digital Liver Cancer Summit 2021** | <https://easl.eu/wp-content/uploads/2021/01/Digital-Liver-Cancer-Summit-2021-Abstract-book.pdf> | The abstract book was searched for the following terms using the advanced search function in Adobe Acrobat:  Primary biliary cholangitis  Primary biliary cirrhosis  PBC  All retrieved abstracts were screened for relevance | 0 | 0 |
|  | **EASL Digital Liver Cancer Summit 2022** | <https://easl.eu/wp-content/uploads/2022/02/LCS-2022-Abstract-book-final.pdf> | The abstract book was searched for the following terms using the advanced search function in Adobe Acrobat:  Primary biliary cholangitis  Primary biliary cirrhosis  PBC  All retrieved abstracts were screened for relevance | 0 | 0 |
| **SLR Update I** | **AASLD Liver Meeting 2023** | <https://aasld.org/sites/default/files/2023-10/the_liver_meeting_boston_massachusetts_nov1_0.pdf> | The abstract book was searched for the following terms using the advanced search function in Adobe Acrobat:  Primary biliary cholangitis  Primary biliary cirrhosis  PBC  All retrieved abstracts were screened for relevance | 44 | 11 |
|  | **AASLD Liver Meeting 2023 Late-breakers** | <https://aasld.org/sites/default/files/2023-11/SLD-625%20Late%20Breaking%20Abstract.pdf> | The abstract book was searched for the following terms using the advanced search function in Adobe Acrobat:  Primary biliary cholangitis  Primary biliary cirrhosis  PBC  All retrieved abstracts were screened for relevance | 3 | 3 |
|  | **EASL International Liver Congress 2023** | <https://easlcongress.eu/wp-content/uploads/2023/06/EASL_2023_Congress_Abstracts_version2_reduced_web-compressed-1.pdf> | The abstract book was searched for the following terms using the advanced search function in Adobe Acrobat:  Primary biliary cholangitis  Primary biliary cirrhosis  PBC  All retrieved abstracts were screened for relevance | 67 | 1 |
|  | **EASL Digital Liver Cancer Summit 2023** | <https://easl.eu/wp-content/uploads/2023/04/EASL_Liver_Cancer_Summit_2023-Abstract-book-FINAL_15.04.2023-b.pdf> | The abstract book was searched for the following terms using the advanced search function in Adobe Acrobat:  Primary biliary cholangitis  Primary biliary cirrhosis  PBC  All retrieved abstracts were screened for relevance | 2 | 0 |
| **SLR Update II** | **AASLD Liver Meeting** | N/A | N/A – No further meetings have been held since SLR Update I | N/A | N/A |
|  | **EASL International Liver Congress 2024** | <https://www.easlcongress.eu/wp-content/uploads/2024/05/EASL_2024_Abstract_version1-1_27-05.pdf> | The abstract book was searched for the following terms using the advanced search function in Adobe Acrobat:  Primary biliary cholangitis  Primary biliary cirrhosis  PBC  All retrieved abstracts were screened for relevance | 93 | 22^a^ |
|  | **EASL Liver Cancer Summit 2023** | <https://easl.eu/wp-content/uploads/2024/02/EASL_Liver_Cancer_Summit_2024-Abstract-Book.pdf> | The abstract book was searched for the following terms using the advanced search function in Adobe Acrobat:  Primary biliary cholangitis  Primary biliary cirrhosis  PBC  All retrieved abstracts were screened for relevance | 0 | 0 |
| **SLR Update III** | **AASLD Liver Meeting** | <https://www.aasld.org/sites/default/files/2024-10/1_the_liver_meeting_2024_abstracts.pdf> | The abstract book was searched for the following terms using the advanced search function in Adobe Acrobat:  Primary biliary cholangitis  Primary biliary cirrhosis  PBC  All retrieved abstracts were screened for relevance | 73 | 1^a^ |
|  | **EASL International Liver Congress 2025** | [EASL Congress 2025 - EASL-The Home of Hepatology.](https://easl.eu/event/easl-congress-2025/) | The abstract book was searched for the following terms using the advanced search function in Adobe Acrobat:  Primary biliary cholangitis  Primary biliary cirrhosis  PBC  All retrieved abstracts were screened for relevance | 65 | 7^a^ |
|  | **EASL Liver Cancer Summit 2025** | [EASL_LCS_2025_abstract_book.pdf](https://easl.eu/wp-content/uploads/2025/02/EASL_LCS_2025_abstract_book.pdf) | The abstract book was searched for the following terms using the advanced search function in Adobe Acrobat:  Primary biliary cholangitis  Primary biliary cirrhosis  PBC  All retrieved abstracts were screened for relevance | 0 | 0 |

Abstracts were not obtainable for the International Conference on Hepatology and Liver Disease or the World Conference of Gastroenterology and Hepatology. ^a^Prior to evidence prioritization.

AASLD: American Association for the Study of Liver Diseases; EASL: European Association for the Study of the Liver; N/A: not applicable; PBC: primary biliary cholangitis; PSC: primary sclerosing cholangitis; SLR: systematic literature review.

**Supplementary Table 9**. Search terms used for ClinicalTrials.gov

| **SLR** | **Condition** | **Phases** | **Study results** | **Recruitment status** | **Results posted date** | **Retrieved records** | **Included records** |
| --- | --- | --- | --- | --- | --- | --- | --- |
| **Original SLR (12.19.2022)** | "primary biliary cholangitis" OR "primary biliary cirrhosis" OR "primary biliary cholestasis" OR "PBC" | Any | "Studies With Results" | All | First posted: Any time | 25 | 6 |
| **SLR Update I (12.05.2023)** |  |  |  |  | Last update posted: November 2022–onwards | 1 | 1 |
| **SLR Update II (07.12.2024)** |  |  |  |  | Last update posted: December 2023–onwards | 2 | 1^a^ |
| **SLR Update III (08.15.2025)** |  |  |  |  | Last update posted: December 2024–onwards | 9 | 2^a^ |

^a^Prior to evidence prioritization.

PBC: primary biliary cholangitis; SLR: systematic literature re

**Supplementary Table 10.** Studies included and prioritized for extraction in the SLR

| **Study Name** | **#** | **Reference** | **Captured In** |
| --- | --- | --- | --- |
| **Askari 2010** | 1 | Askari F, Innis D, Dick RB, et al. Treatment of primary biliary cirrhosis with tetrathiomolybdate: results of a double-blind trial. Translational Research: The Journal Of Laboratory & Clinical Medicine 2010;155:123-30. | Original SLR |
| **BEZURSO (NCT01654731)** | 2 | **Corpechot C, Chazouilleres O, Rousseau A, et al. A Placebo-Controlled Trial of Bezafibrate in Primary Biliary Cholangitis. New England Journal of Medicine 2018;378:2171-2181.** | Original SLR |
|  | 3 | Corpechot C, Rousseau A, Lemoinne S, et al. Bezafibrate add-on therapy in high-risk primary biliary cholangitis is associated with an improvement of fibrometer and fibrometer-VCTE, two high-accuracy non-invasive fibrosis tests extensively validated in frequent chronic liver diseases. Journal of hepatology 2019;70:387‐. | Original SLR |
|  | 4 | Kremer AE, Le Cleac HA, Lemoinne S, et al. Antipruritic effect of bezafibrate and serum autotaxin measures in patients with primary biliary cholangitis. Gut. 2018. | Original SLR |
|  | 5 | Nct. Phase 3 Study of Bezafibrate in Combination With Ursodeoxycholic Acid in Primary Biliary Cirrhosis. https://clinicaltrials.gov/show/NCT01654731 2012. | Original SLR |
| **Li 2022 (ChiCTR1800020160)** | 6 | **Li C, Zheng K, Chen Y, et al. A randomized, controlled trial on fenofibrate in primary biliary cholangitis patients with incomplete response to ursodeoxycholic acid. Therapeutic Advances in Chronic Disease 2022;13:20406223221114198.** | Original SLR |
|  | 7 | ChiCtr. Clinical research for fenofibrate in the treatment of refractory primary biliary cirrhosis. https://trialsearch.who.int/Trial2.aspx?TrialID=ChiCTR1800020160 2018. | Original SLR |
| **COBALT** (NCT02308111) | 8 | **Kowdley, KV; Brookhart, MA; Hirschfield, G et al. Efficacy of obeticholic acid (oca) versus placebo and external control (EC) on clinical outcomes in primary biliary cholangitis (PBC). Hepatology (Baltimore, Md). 2023;77(5):E144‐E146.** | SLR Update I |
|  | 9 | ClinicalTrialsgov. Phase 4 Study of Obeticholic Acid Evaluating Clinical Outcomes in Patients With Primary Biliary Cholangitis. 2023. Available from: https://ClinicalTrials.gov/show/NCT02308111. | SLR Update I |
|  | 10 | Kowdley, K; Hirschfield, G; Mayne, T *et al.* 4538-C \| Clinical trial and real-world outcomes in patients with primary biliary cholangitis treated with obeticholic acid per current US label. 2023. AASLD 2023; | SLR Update I |
|  | 11 | Kowdley, K; Mayne, T; Wheeler, D et al. 4578-C \| The challenge of confirmatory trials in rare disease: Lessons learned from the cobalt trial in primary biliary cholangitis 2023. AASLD 2023; | SLR Update I |
|  | 12 | Kowdley KV, Hirschfield G, Mayne T, et al. Clinical Trial And Real-World Outcomes In Patients With Primary Biliary Cholangitis Treated With Obeticholic Acid Per Current Us Label. Hepatology 2023;78:S2039-S2041. | SLR Update II |
|  | 13 | Kowdley KV, Hirschfield G, Coombs C, et al. COBALT: A Confirmatory Trial of Obeticholic Acid in Primary Biliary Cholangitis With Placebo and External Controls. American Journal of Gastroenterology 2025;120:390-400 | SLR Update III |
| **Combes 2005** | 14 | Combes B, Emerson SS, Flye NL, et al. Methotrexate (MTX) plus ursodeoxycholic acid (UDCA) in the treatment of primary biliary cirrhosis. Hepatology 2005;42:1184-93. | Original SLR |
| **ELATIVE (NCT04526665)** | 15 | **Kowdley, KV; Bowlus, CL; Levy, C et al. Efficacy and Safety of Elafibranor in Primary Biliary Cholangitis. N Engl J Med. 2023;13:13.** | SLR Update I |
|  | 16 | Bowlus, C; Kowdley, K; Levy C. et al. 5007 \| Ffficacy and safety of elafibranor in primary biliary cholangitis: Results from the ELATIVE™ double-blind, randomized, placebo-controlled phase 3 trial 2023. AASLD 2023; | SLR Update I |
|  | 17 | Euctr BE. A Double-blind Randomized, Placebo-Controlled Study and Open-label Long Term Extension to Evaluate the Efficacy and Safety of Elafibranor 80 mg in Patients with Primary Biliary Cholangitis with Inadequate Response or Intolerance to Ursodeoxycholic Acid. https://trialsearch.who.int/Trial2.aspx?TrialID=EUCTR2019-004941-34-BE 2020. | Original SLR |
|  | 18 | Nct. Phase 3 Study of Elafibranor in Patients With Primary Biliary Cholangitis (PBC). https://clinicaltrials.gov/show/NCT04526665 2020. | Original SLR |
|  | 19 | Bowlus, CL, Kowdley, KV, Levy C, et al. Efficacy and safety of elafibranor in primary biliary cholangitis: results from the ELATIVE^TM^ double-blind, randomized, placebo-controlled phase 3 trial. Hepatology 2024;79:e40-e42. | SLR Update II |
|  | 20 | Bowlus CL, Levy C, Akarca U, et al. LBP-006 - Efficacy of elafibranor in primary biliary cholangitis: results from the variable double-blind period of ELATIVE^®^, a randomised, placebo-controlled phase III trial, In EASL International Liver Congress, 2024. | SLR Update II |
|  | 21 | Kremer AE, Levy C, Mayo MJ, et al. LBP-028 - Effect of elafibranor on pruritus in primary biliary cholangitis: symptom severity and quality of life measurements from the phase III ELATIVE^®^ trial, In EASL International Liver Congress, 2024 | SLR Update II |
|  | 22 | Mayo MJ, Pedersen M, Tonev D, et al. THU-105 - Improvement in lipid profiles with elafibranor treatment in primary biliary cholangitis during the phase III Elative® trial, In EASL International Liver Congress, 2024. | SLR Update II |
|  | 23 | Sonderup M, Calvaruso V, Antunes N, et al. OS-016 - Elafibranor efficacy in primary biliary cholangitis according to biochemical response criteria in the phase III ELATIVE® trial, In EASL International Liver Congress, 2024. | SLR Update II |
|  | 24 | Kowdley K, Lawitz E, Spearman C, et al. One-Year Treatment with Elafibranor in the Phase I I I Elative Trial Improves Globe and Uk-Pbc Prognostic Scores. Hepatology 2024;80(Supplement 1):S1790-S1791. | SLR Update III |
|  | 25 | Kremer AE, Kowdley KV, Levy C, et al. Effect of Elafibranor on Pruritus in Primary Biliary Cholangitis: Symptom Severity and Quality of Life Measurements from the Phase Iii Elative Trial. Gut 2024;73(Supplement 3):A22. | SLR Update III |
|  | 26 | Levy C, Bowlus C, Lawitz E, et al. Beyond the Mean: Exploring the Impact of Baseline Alkaline Phosphatase Levels on Endpoints in Primary Biliary Cholangitis ? Hepatology 2024;80(Supplement 1):S1767-S1769. | SLR Update III |
|  | 27 | Mayo MJ, Antunes N, Pedersen M, et al. Improvement in Lipid Profiles with Elafibranor Treatment in Primary Biliary Cholangitis during the Phase Iii Elative Trial. Gut 2024;73(Supplement 3):A22-A23. | SLR Update III |
|  | 28 | Sonderup M, Spearman CW, Calvaruso V, et al. Elafibranor Efficacy in Primary Biliary Cholangitis According to Biochemical Response Criteria in the Phase Iii Elative Trial. Gut 2024;73(Supplement 3):A25. | SLR Update III |
|  | 29 | Schattenberg JM, Andreone P, Heneghan M, et al. THU-313 Treatment with elafibranor has no impact on bone health in patients with primary biliary cholangitis. Journal of hepatology 2025;82:S323-S323. | SLR Update III |
| **ENHANCE (NCT03602560)** | 30 | **Hirschfield, GM; Shiffman, ML; Gulamhusein, A *et al.* Seladelpar efficacy and safety at 3 months in patients with primary biliary cholangitis: ENHANCE, a phase 3, randomized, placebo-controlled study. Hepatology. 2023;78(2):397-415.** | SLR Update I |
|  | 31 | Nct. Seladelpar in Subjects With Primary Biliary Cholangitis (PBC) and an Inadequate Response to or an Intolerance to Ursodeoxycholic Acid (UDCA). https://clinicaltrials.gov/show/NCT03602560 2018. | Original SLR |
|  | 32 | Anonymous. ENHANCE: Safety and Efficacy of Seladelpar in Patients With Primary Biliary Cholangitis-A Phase 3, International, Randomized, Placebo-Controlled Study. Gastroenterology & Hepatology 2021;17:5-6. | Original SLR |
|  | 33 | Choi Y-J, Johnson JD, Schwab A, et al. Seladelpar, a PPAR-delta agonist, improves inflammatory lipid mediators in the serum metabolome in patients with primary biliary cholangitis (PBC), in Hepatology, Wiley 111 River St, Hoboken 07030-5774, NJ USA, 2022. | Original SLR |
| **FITCH (NCT02701166)** | 34 | **de Vries E, Bolier R, Goet J, et al. Fibrates for Itch (FITCH) in Fibrosing Cholangiopathies: a Double-Blind, Randomized, Placebo-Controlled Trial. Gastroenterology 2021;160:734‐743.e6.** | Original SLR |
|  | 35 | Euctr NL. The effect of bezafibrate on itch in a subset of liver diseases. https://trialsearch.who.int/Trial2.aspx?TrialID=EUCTR2014-001438-27-NL 2015. | Original SLR |
|  | 36 | Nct. The Effect of Bezafibrate on Cholestatic Itch. https://clinicaltrials.gov/show/NCT02701166 2016. | Original SLR |
| **GLIMMER (NCT02966834)** | 37 | **Levy C, Kendrick S, Bowlus CL, et al. GLIMMER: A randomized Phase 2b dose-ranging trial of linerixibat in primary biliary cholangitis patients with pruritus. Clinical gastroenterology and hepatology : the official clinical practice journal of the American Gastroenterological Association. 2022;04.** | Original SLR |
|  | 38 | Carreño F, Mehta R, Ribeiro A, et al. Linerixibat dose-response analysis of C4 concentrations as a quantitative approach to predict gastrointestinal tolerability. Journal of Hepatology 2022:S328-S329. | Original SLR |
|  | 39 | Euctr ES. Dose response study of GSK2330672 for the treatment of pruritus in patients with primary biliary cholangitis – The GLIMMER Study. https://trialsearch.who.int/Trial2.aspx?TrialID=EUCTR2016-002416-41-ES 2016. | Original SLR |
|  | 40 | Fettiplace, J; Swift, B; Zhang, S *et al.* Investigation of Linerixibat 40 Mg BID for Cholestatic Pruritus of Primary Biliary Cholangitis; Further Data From the Phase 2b GLIMMER Study to Support the Phase 3 GLISTEN Study. American Journal of Gastroenterology. 2022;117(10 Supplement 2):S16. | SLR Update I |
|  | 41 | Fettiplace J, Swift B, Zhang S, et al. Investigation of linerixibat 40mg BID for cholestatic pruritus of primary biliary cholangitis (PBC); further data from the phase 2b GLIMMER study to support the phase 3 GLISTEN study. Journal of Hepatology 2022;77(Supplement 1):S335-S336. | Original SLR |
|  | 42 | Jia J, Jones D, Maltzahn RV, et al. Improvement in itch correlates with improved sleep in GLIMMER, a Phase 2b trial of linerixibat for the treatment of cholestatic pruritus in primary biliary cholangitis (PBC). Hepatology international 2022;16:S297‐S298. | Original SLR |
|  | 43 | Jia J, Levy C, Kendrick S, et al. GLIMMER: a randomized double-blind placebo-controlled study of linerixibat, an ileal bile acid transporter inhibitor, in the treatment of cholestatic pruritus in primary biliary cholangitis (PBC). Hepatology international 2022;16:S296‐S297. | Original SLR |
|  | 44 | Jones DE, Maltzahn RV, Smith H, et al. 5-D Itch and Quality of Life in Pbc Patients with Cholestatic Pruritus from the Phase 2b Study Glimmer. Gastroenterology 2022;162(7 Supplement):S-1285-S-1286. | Original SLR |
|  | 45 | Nct. Dose Response Study of GSK2330672 for the Treatment of Pruritus in Patients With Primary Biliary Cholangitis. https://clinicaltrials.gov/show/NCT02966834 2016. | Original SLR |
|  | 46 | Smith H, Fettiplace J, von Maltzahn R, et al. More than just an itch: impact of cholestatic pruritus in primary biliary cholangitis (PBC) on health-related quality of life (HRQoL). Journal of Hepatology 2022;77(Supplement 1):S327-S328. | Original SLR |
|  | 47 | Tanaka, A; Atsukawa, M; Tsuji, K *et al.* Japanese subgroup analysis of GLIMMER: A global Phase IIb study of linerixibat for the treatment of cholestatic pruritus in patients with primary biliary cholangitis. Hepatology Research. 2023;53(7):629-640. | SLR Update I |
|  | 48 | Tanaka, A; Suyama, A; Ito, H *et al.* Investigation of linerixibat 40 mg BID (twice daily) for cholestatic pruritus of primary biliary cholangitis (PBC) in the glimmer phase 2b study; further analysis of the Japanese subgroup. Hepatology International. 2023;17(Supplement 1):S185-S186. | SLR Update I |
|  | 49 | Fettiplace J, Swift B, Zhang S, et al. Investigation of linerixibat 40 mg BID for cholestatic pruritus of primary biliary cholangitis (PBC); further data from the Phase 2b GLIMMER study to support the Phase 3 GLISTEN study. Digestive and Liver Disease 2025;57(Supplement 1):S27-S28. | SLR Update III |
| **Gonzalez-Koch 1997** | 50 | Gonzalez-Koch A, Brahm J, Antezana C, et al. The combination of ursodeoxycholic acid and methotrexate for primary biliary cirrhosis is not better than ursodeoxycholic acid alone. Journal of Hepatology 1997;27:143-9. | Original SLR |
| **Study 213** | 51 | **Hejda, V; Louvet, A; Civitarese, A *et al.* Results from a planned interim analysis of a randomized, double-blind, active-controlled trial evaluating the effects of obeticholic acid and bezafibrate on serum biomarkers in primary biliary cholangitis. Journal of Hepatology. 2023;78(Supplement 1):S45.** | **SLR Update I** |
|  | 52 | Jones D, Hejda V, Louvet A, et al. Results From A Planned Interim Analysis Of A Randomized, Double-Blind, Active-Controlled Trial Evaluating The Effects Of Obeticholic Acid And Bezafibrate On Serum Biomarkers In Primary Biliary Cholangitis. Gut 2023;72:A7. | SLR Update II |
|  | 53 | Bonder A, Jones DE, Levy C, et al. 155 Metabolic Outcomes And Key Efficacy Data From A Phase 2 Trial Evaluating Combination Obeticholic Acid And Bezafibrate In Patients With Primary Biliary Cholangitis. Gastroenterology 2024;166:S-1542. | SLR Update II |
|  | 54 | Jones DE, Corpechot C, Hejda V, et al. LBP-027 - Combined effect of obeticholic acid and bezafibrate in patients with primary biliary cholangitis and inadequate response or intolerance to ursodeoxycholic acid: 6-month results from a phase 2 trial, In EASL International Liver Congress, 2024. | SLR Update II |
|  | 55 | Ballard M, Jones DE, Louvet A, et al. Combined Effect of Obeticholic Acid and Bezafibrate in Patients With Primary Biliary Cholangitis and Inadequate Response or Intolerance to Ursodeoxycholic Acid: 6-Month Results From a Phase 2 Trial. American Journal of Gastroenterology 2024;119(10 Supplement):S82-S83. | SLR Update III |
| **Hosonuma 2015** | 56 | Hosonuma K, Sato K, Yamazaki Y, et al. A prospective randomized controlled study of long-term combination therapy using ursodeoxycholic acid and bezafibrate in patients with primary biliary cirrhosis and dyslipidemia. American Journal of Gastroenterology 2015;110:423-31. | Original SLR |
| **Ikeda 1996** | 57 | Ikeda T, Tozuka S, Noguchi O, et al. Effects of additional administration of colchicine in ursodeoxycholic acid-treated patients with primary biliary cirrhosis: a prospective randomized study. Journal of Hepatology 1996;24:88-94. | Original SLR |
| **Iwasaki 2008** | 58 | Iwasaki S, Ohira H, Nishiguchi S, et al. The efficacy of ursodeoxycholic acid and bezafibrate combination therapy for primary biliary cirrhosis: a prospective, multicenter study. Hepatology research 2008;38:557‐564. | Original SLR |
| **Kanda 2003** | 59 | Kanda T, Yokosuka O, Imazeki F, et al. Bezafibrate treatment: a new medical approach for PBC patients? Journal of Gastroenterology 2003;38:573-8. | Original SLR |
| **Kurihara 2000** | 60 | Kurihara T, Niimi A, Maeda A, et al. Bezafibrate in the treatment of primary biliary cirrhosis: comparison with ursodeoxycholic acid. American Journal of Gastroenterology 2000;95:2990-2. | Original SLR |
| **Levy 2023 (Study 213, Study 214)** | 61 | **Levy C.; Hejda V.; Louvet A. et al. 5019-C \| Combined effect of obeticholic acid and bezafibrate in patients with primary biliary cholangitis and inadequate response to or intolerance of ursodeoxycholic acid: results from two phase 2 clinical trials 2023. AASLD 2023;** | **SLR Update I** |
|  | 62 | Levy C, Hejda V, Louvet A, et al. Combined Effect Of Obeticholic Acid And Bezafibrate In Patients With Primary Biliary Cholangitis And Inadequate Response To Or Intolerance Of Ursodeoxycholic Acid: Results From Two Phase 2 Clinical Trials. Hepatology 2024;79:E52. | SLR Update II |
| **Liberopoulos 2010** | 63 | Liberopoulos EN, Florentin M, Elisaf MS, et al. Fenofibrate in primary biliary cirrhosis: a pilot study. Open cardiovascular medicine journal 2010;4:120‐126. | Original SLR |
| **Lim 1997** | 64 | Lim AG, Wolfhagen FH, Verma A, et al. Soluble intercellular adhesion molecule-1 in primary biliary cirrhosis: effect of ursodeoxycholic acid and immunosuppressive therapy. European Journal of Gastroenterology & Hepatology 1997;9:155-61. | Original SLR |
| **Nakai 2000** | 65 | Nakai S, Masaki T, Kurokohchi K, et al. Combination therapy of bezafibrate and ursodeoxycholic acid in primary biliary cirrhosis: a preliminary study. Official journal of the American College of Gastroenterology\| ACG 2000;95:326-327. | Original SLR |
| **Hirschfield 2015 (NCT00550862)** | 66 | **Hirschfield GM, Mason A, Luketic V, et al. Efficacy of obeticholic acid in patients with primary biliary cirrhosis and inadequate response to ursodeoxycholic acid. Gastroenterology 2015;148:751‐61.e8.** | Original SLR |
|  | 67 | Nct. Study of INT 747 in Combination With URSO in Patients With Primay Biliary Cirrhosis (PBC). https://clinicaltrials.gov/show/NCT00550862 2007. | Original SLR |
| **Hirschfield 2021 (NCT00746486)** | 68 | **Hirschfield GM, Beuers U, Kupcinskas L, et al. A placebo-controlled randomised trial of budesonide for PBC following an insufficient response to UDCA. Journal of Hepatology 2021;74:321-329.** | Original SLR |
|  | 69 | Euctr ES. Double-blind, randomised, placebo-controlled, multi-centre phase III clinical study comparing the combination of ursodeoxycholic acid capsules plus budesonide capsules to ursodeoxycholic acid capsules plus placebo in the treatment of primary biliary cirrhosis - Ursodeoxycholic acid plus budesonide versus. ursodeoxycholic acid alone in PBC. https://trialsearch.who.int/Trial2.aspx?TrialID=EUCTR2007-004040-70-ES 2008. | Original SLR |
|  | 70 | Nct. Ursodeoxycholic Acid Plus Budesonide Versus Ursodeoxycholic Acid Alone in Primary Biliary Cirrhosis (PBC). https://clinicaltrials.gov/show/NCT00746486 2008. | Original SLR |
| **HAART (NCT01614405)** | 71 | **Lytvyak E, Hosamani I, Montano-Loza AJ, et al. Randomized clinical trial: Combination antiretroviral therapy with tenofovir-emtricitabine and lopinavir-ritonavir in patients with primary biliary cholangitis. Canadian Liver Journal 2019;2:31-44** | Original SLR |
|  | 72 | Nct. Highly Active Antiretroviral Therapy for Patients With Primary Biliary Cirrhosis. https://clinicaltrials.gov/show/NCT01614405 2011. | Original SLR |
| **Hegade 2017 (NCT01899703)** | 73 | **Hegade VERSUS, Kendrick SF, Dobbins RL, et al. Effect of ileal bile acid transporter inhibitor GSK2330672 on pruritus in primary biliary cholangitis: a double-blind, randomised, placebo-controlled, crossover, phase 2a study. Lancet 2017;389:1114-1123.** | Original SLR |
|  | 74 | Hegade VERSUS, Kendrick SF, Dobbins RL, et al. BAT117213: Ileal bile acid transporter (IBAT) inhibition as a treatment for pruritus in primary biliary cirrhosis: study protocol for a randomised controlled trial. BMC Gastroenterology 2016;16:71. | Original SLR |
|  | 75 | Nct. A Study to Evaluate the Safety, Tolerability, Pharmacokinetics (PK) and Pharmacodynamics (PD) of Repeat Doses of GSK2330672 Administration in Subjects With Primary Biliary Cirrhosis (PBC) and Symptoms of Pruritus. https://clinicaltrials.gov/show/NCT01899703 2013. | Original SLR |
| **CLARITY (NCT01904058)** | 76 | Nct. Phase 2 Study to Evaluate LUM001 in Combination With Ursodeoxycholic Acid in Patients With Primary Biliary Cirrhosis. https://clinicaltrials.gov/show/NCT01904058 2013. | Original SLR |
| **Mayo 2018 (NCT02026401)** | 77 | **Mayo MJ, Wigg AJ, Leggett BA, et al. NGM282 for Treatment of Patients With Primary Biliary Cholangitis: A Multicenter, Randomized, Double-Blind, Placebo-Controlled Trial. Hepatology Communications 2018;2:1037-1050** | Original SLR |
|  | 78 | Nct. Phase 2 Study of NGM282 in Patients With Primary Biliary Cirrhosis. https://clinicaltrials.gov/show/NCT02026401 2013. | Original SLR |
| **Schramm 2022 (NCT02516605)** | 79 | **Schramm C, Wedemeyer H, Mason A, et al. Farnesoid X receptor agonist tropifexor attenuates cholestasis in a randomised trial in patients with primary biliary cholangitis. JHEP Reports : Innovation in Hepatology / EASL 2022;4:100544.** | Original SLR |
|  | 80 | Nct. A Multi-part, Double Blind Study to Assess Safety, Tolerability and Efficacy of Tropifexor (LJN452) in PBC Patients. https://clinicaltrials.gov/show/NCT02516605 2015. | Original SLR |
| **Jones 2017 (NCT02609048)** | 81 | Jones D, Boudes PF, Swain MG, et al. Seladelpar (MBX-8025), a selective PPAR-delta agonist, in patients with primary biliary cholangitis with an inadequate response to ursodeoxycholic acid: a double-blind, randomised, placebo-controlled, phase 2, proof-of-concept study. The lancet gastroenterology and hepatology 2017;2:716‐726. | Original SLR |
| **NCT02943447** | 82 | Nct. Safety, Tolerability, and Efficacy of GS 9674 in Adults With Primary Biliary Cholangitis Without Cirrhosis. https://clinicaltrials.gov/show/NCT02943447 2016. | Original SLR |
| **Bowlus 2022 (NCT02955602)** | 83 | **Bowlus CL, Galambos MR, Aspinall RJ, et al. A phase II, randomized, open-label, 52-week study of seladelpar in patients with primary biliary cholangitis. Journal of hepatology 2022;77:353‐364.** | Original SLR |
|  | 84 | Bowlus CL, Neff GW, Aspinall R, et al. Efficacy and safety of seladelpar, a selective peroxisome proliferator-activated receptor delta agonist, in primary biliary cholangitis: 52-week analysis of an ongoing international, randomized, dose ranging phase 2 study. Hepatology (Baltimore, Md.) 2018;68:1446A‐1447A. | Original SLR |
|  | 85 | Hansen B, Watkins E, Yang K, et al. Seladelpar treatment of patients with primary biliary cholangitis (PBC) for 2 years improves the GLOBE PBC score and predicts improved transplant-free survival. Journal of Hepatology 2022;77(Supplement 1):S322-S323. | Original SLR |
|  | 86 | Kremer AE, Mayo MJ, Hirschfield G, et al. Seladelpar improved measures of pruritus, sleep, and fatigue and decreased serum bile acids in patients with primary biliary cholangitis. Liver International 2022;42(1):112-123. | Original SLR |
|  | 87 | Mayo MJ, Vierling JM, Bowlus CL, et al. Long-term safety and efficacy of seladelpar in patients with primary biliary cholangitis (pbc): 2-year results from a long-term study. Hepatology (Baltimore, Md.) 2021;74:71A‐73A. | Original SLR |
|  | 88 | Nct. Seladelpar (MBX-8025) in Subjects With Primary Biliary Cholangitis (PBC). https://clinicaltrials.gov/show/NCT02955602 2016. | Original SLR |
| **Bowlus 2022 (NCT02955602); ENHANCE (NCT03602560)** | 89 | **Bowlus CL, Choi Y-J, Yang K, et al. Seladelpar improved the lipid profile of patients with primary biliary cholangitis (pbc): results from phase 2 and 3 clinical studies, In Hepatology, WILEY 111 River St, Hoboken 07030-5774, NJ USA, 2022.** | Original SLR |
|  | 90 | Gulamhusein A, Neff G, Goel A, et al. Treatment with seladelpar in patients with primary biliary cholangitis (PBC) and prior experience with obeticholic acid (OCA) or fibrates. J Hepatol 2021;75:S690-S691. | Original SLR |
| **EPICS (NCT03112681)** | 91 | **Vuppalanchi R, Caldwell SH, Pyrsopoulos N, et al. Proof-of-concept study to evaluate the safety and efficacy of saroglitazar in patients with primary biliary cholangitis. Journal of hepatology 2022;76:75‐85..** | Original SLR |
|  | 92 | Nct. A Study to Evaluate Safety, Tolerability and Efficacy of Saroglitazar Magnesium in Patients With Primary Biliary Cholangitis (EPICS). https://clinicaltrials.gov/show/NCT03112681 2017. | Original SLR |
| **Schattenberg 2021 (NCT03124108)** | 93 | **Schattenberg JM, Pares A, Kowdley KV, et al. A randomized placebo-controlled trial of elafibranor in patients with primary biliary cholangitis and incomplete response to UDCA. Journal of hepatology 2021;74:1344‐1354.** | Original SLR |
|  | 94 | Euctr ES. Phase 2 Efficacy & Safety Study of Elafibranor in patients with PBC. https://trialsearch.who.int/Trial2.aspx?TrialID=EUCTR2016-003817-80-ES 2017. | Original SLR |
|  | 95 | Nct. Study to Evaluate the Efficacy and Safety of Elafibranor in Patients With Primary Biliary Cholangitis (PBC) and Inadequate Response to Ursodeoxycholic Acid. https://clinicaltrials.gov/show/NCT03124108 2017. | Original SLR |
| **NCT03226067** | 96 | **Invernizzi, P; Carbone, M; Jones, D et al. Setanaxib, a first-in-class selective NADPH oxidase 1/4 inhibitor for primary biliary cholangitis: A randomized, placebo-controlled, phase 2 trial. Liver International. 2023;43(7):1507-1522.** | SLR Update I |
|  | 97 | NCT. Study to Assess Safety & Efficacy of GKT137831 in Patients With Primary Biliary Cholangitis Receiving Ursodiol. 2017. | Original SLR |
|  | 98 | Euctr BE. A study assessing the efficacy and safety of GKT137831 in Patients with Primary Biliary Cholangitis Receiving Ursodeoxycholic Acid and with Persistently Elevated Alkaline Phosphatase. https://trialsearch.who.int/Trial2.aspx?TrialID=EUCTR2016-004599-23-BE 2017. | Original SLR |
|  | 99 | Jones, D; Carbone, M; Invernizzi, P et al. Impact of setanaxib on quality of life outcomes in primary biliary cholangitis in a phase 2 randomized controlled trial. Hepatology communications. 2023;7(3):e0057. | SLR Update I |
|  | 100 | Jones D, Carbone M, Invernizzi P, et al. Quality of life outcomes in patients with primary biliary cholangitis treated with setanaxib: post-hoc results from a phase 2 randomised, placebo-controlled trial. Journal of Hepatology 2022;77(Supplement 1):S94-S95. | Original SLR |
|  | 101 | Levy C, Carbone M, Wiesel P, et al. Setanaxib reduces cholestasis and fatigue in patients with primary biliary cholangitis and liver stiffness >=9.6 kpa: Post-hoc analyses from a randomized, controlled, phase 2 trial. Hepatology 2021;74(SUPPL 1):782A-783A. | Original SLR |
| **Xiang 2021 (NCT03345589)** | 102 | **Xiang X, Yang X, Shen M, et al. Ursodeoxycholic Acid at 18-22 mg/kg/d Showed a Promising Capacity for Treating Refractory Primary Biliary Cholangitis. Canadian Journal of Gastroenterology & Hepatology 2021;2021:6691425.** | Original SLR |
|  | 103 | Nct. A Trial of 18-22mg/kg/d Ursodeoxycholic in Refractory Primary Biliary Cholangitis. https://clinicaltrials.gov/show/NCT03345589 2017. | Original SLR |
| **NCT03394924** | 104 | Nct. A Study to Assess the Safety, Tolerability, Pharmacokinetics and Efficacy of EDP-305 in Subjects With Primary Biliary Cholangitis. https://clinicaltrials.gov/show/NCT03394924 2018. | Original SLR |
| **NCT03633227** | 105 | Nct. Study of OCA Evaluating Pharmacokinetics and Safety in Patients With PBC and Hepatic Impairment. https://clinicaltrials.gov/show/NCT03633227 2018. | Original SLR |
| **POISE (NCT01473524)** | 106 | **Nevens F, Andreone P, Mazzella G, et al. A Placebo-Controlled Trial of Obeticholic Acid in Primary Biliary Cholangitis. New England journal of medicine 2016;375:631‐643.** | Original SLR |
|  | 107 | Andreone P, Floreani A, Invernizzi P, et al. Durable response in the markers of cholestasis through 24 months of open-label extension with obeticholic acid in Italian patients with primary biliary cholangitis. Digestive and liver disease 2017;49:e21‐. | Original SLR |
|  | 108 | Andreone P, Floreani A, Invernizzi P, et al. Durable response in the markers of cholestasis through 36 months of open-label extension with obeticholic acid in Italian patients with primary biliary cholangitis. Digestive and liver disease 2018;50:26‐. | Original SLR |
|  | 109 | Bonder A.; Wheeler, D; Nair, R et al. 4545-C \| Effect of obeticholic acid on prognostic thresholds of gamma-glutamyl transferase and alkaline phosphatase levels: sub-analysis of the phase 3 poise trial in primary biliary cholangitis 2023. AASLD 2023; | SLR Update I |
|  | 110 | Bowlus C, Trauner M, Liberman A, et al. Long-Term Efficacy and Safety of Obeticholic Acid in Patients with PBC from POISE Grouped by Risk of Disease Progression. Zeitschrift fur Gastroenterologie 2021;59(1):e14-e15. | Original SLR |
|  | 111 | Bowlus CL, Pockros PJ, Kremer AE, et al. Three years of Obeticholic Acid (OCA) Therapy Results in Histological Improvements in Patients with Primary Biliary Cholangitis: further Analysis of the POISE Biopsy Substudy. Zeitschrift fur Gastroenterologie 2019;57:e9‐. | Original SLR |
|  | 112 | Bowlus CL, Pockros PJ, Kremer AE, et al. Long-Term Obeticholic Acid Therapy Improves Histological Endpoints in Patients With Primary Biliary Cholangitis. Clinical gastroenterology and hepatology 2020;18:1170‐1178.e6. | Original SLR |
|  | 113 | Bowlus CL, Trauner M, Liberman A, et al. Long-term efficacy and safety of obeticholic acid in patients with PBC from the POISE trial grouped biochemically by risk of disease progression. Digestive and Liver Disease 2021;53(Supplement 1):S18. | Original SLR |
|  | 114 | Carbone M, Harms MH, Lammers WJ, et al. Clinical application of the GLOBE and United Kingdom-primary biliary cholangitis risk scores in a trial cohort of patients with primary biliary cholangitis. Hepatology Communications 2018;2:683-692. | Original SLR |
|  | 115 | Floreani A, Bowlus CL, Trauner M, et al. Long-Term Efficacy And Safety Of Obeticholic Acid In Patients With Pbc From The Poise Trial Grouped Biochemically By Risk Of Disease Progression. Digestive and Liver Disease 2021;53(Supplement 3):S109. | Original SLR |
|  | 116 | Halilbasic E, Hofer H, Munda P, et al. Durable response in the markers of cholestasis through 18 months of open-label extension with obeticholic acid in Austrian and German patients with primary biliary cholangitis. Zeitschrift fur Gastroenterologie 2017;55. | Original SLR |
|  | 117 | Halilbasic E, Zoller H, Munda P, et al. Durable response in the markers of cholestasis through 36 months of open-label extension with obeticholic acid in Austrian and german patients with primary biliary cholangitis. Zeitschrift fur Gastroenterologie 2018;56:e47‐. | Original SLR |
|  | 118 | Harms MH, Hirschfield GM, Floreani A, et al. Obeticholic acid is associated with improvements in AST-to-platelet ratio index and GLOBE score in patients with primary biliary cholangitis. JHEP Reports : Innovation in Hepatology / EASL 2021;3:100191. | Original SLR |
|  | 119 | Hirschfield G, Carbone M, Jones D, et al. Durability of obeticholic acid response in PBC patients who did not achieve poise trial criteria. Gut 2021;70(SUPPL 1):A13. | Original SLR |
|  | 120 | Hirschfield G, Jones D, Carbone M, et al. Long-Term Efficacy and Safety of Obetichoclic Acid in Primary Biliary Cholangitis: Responder Analysis of over 5 Years of Treatment in the Poise Trial. Journal of the Canadian Association of Gastroenterology. Conference: Canadian Digestive Diseases Week, CDDW 2021;4. | Original SLR |
|  | 121 | Jones D, Carbone M, Mells G, et al. Predicted risk of end stage liver disease utilizing the UK-PBC risk score in PBC patients. Gut 2021;70(SUPPL 1):A153-A154. | Original SLR |
|  | 122 | Kremer A, Hirschfield G, Jones D, et al. Pruritus Experience in Patients with PBC Treated with Obeticholic Acid Through 6 Years: Patient-Reported Quality of Life. Zeitschrift fur Gastroenterologie 2021;59(1):e22. | Original SLR |
|  | 123 | Nct. Phase 3 Study of Obeticholic Acid in Patients With Primary Biliary Cirrhosis. https://clinicaltrials.gov/show/NCT01473524 2011. | Original SLR |
|  | 124 | Pares A, Shiffman M, Vargas V, et al. Reduction and stabilization of bilirubin with obeticholic acid treatment in patients with primary biliary cholangitis. Liver International 2020;40:1121-1129. | Original SLR |
|  | 125 | Trauner M, Nevens F, Shiffman ML, et al. Long-term efficacy and safety of obeticholic acid for patients with primary biliary cholangitis: 3-year results of an international open-label extension study. The lancet. Gastroenterology & hepatology 2019;4:445‐453. | Original SLR |
|  | 126 | Bonder A, Wheeler D, Nair R, et al. Effect Of Obeticholic Acid On Prognostic Thresholds Of Gamma-Glutamyl Transferase And Alkaline Phosphatase Levels: Sub-Analysis Of The Phase 3 Poise Trial In Primary Biliary Cholangitis. Hepatology 2023;78:S2047-S2048. | SLR Update II |
|  | 127 | Gish RG, Wheeler D, Li J, et al. 632 Effect Of Obeticholic Acid On Normalization Of Alanine Aminotransferase And Aspartate Aminotransferase: Subanalysis Of The Phase 3 Poise Trial In Primary Biliary Cholangitis. Gastroenterology 2024;166:S-1554. | SLR Update II |
|  | 128 | Gish RG, Li J, Gasink C, et al. FRI-023 - Effect of obeticholic acid on reduction and normalization of alanine aminotransferase and aspartate aminotransferase in the phase 3 POISE trial in primary biliary cholangitis, In EASL International Liver Congress, 2024. | SLR Update II |
|  | 129 | Bonder A, Wheeler D, Li J, et al. Evaluation of Aspartate Aminotransferase to Platelet Ratio Index and Fibrosis-4 Index Stabilization in the Phase 3 POISE Trial of Obeticholic Acid for the Treatment of Primary Biliary Cholangitis. American Journal of Gastroenterology 2024;119(10 Supplement):S160-S161. | SLR Update III |
|  | 130 | Foston T, Jones DE, Bowlus CL, et al. Obeticholic Acid Normalizes Inflammatory Biomarkers in Patients With Primary Biliary Cholangitis. American Journal of Gastroenterology 2024;119(10 Supplement):S129. | SLR Update III |
|  | 131 | Gish RG, Wheeler D, Li J, et al. Stabilization of Enhanced Liver Fibrosis and Liver Stiffness Measures in the Open-Label Extension of the Phase 3 POISE Trial of Obeticholic Acid for the Treatment of Primary Biliary Cholangitis. American Journal of Gastroenterology 2024;119(10 Supplement):S161-S162. | SLR Update III |
|  | 132 | Gish RG, Wheeler D, Li J, et al. Effect of Obeticholic Acid on Reduction and Normalization of Alanine Aminotransferase and Aspartate Aminotransferase in the Phase 3 POISE Trial in Primary Biliary Cholangitis. American Journal of Gastroenterology 2024;119(10 Supplement):S88-S89. | SLR Update III |
|  | 133 | Jones D, Goel A, Moore A, et al. Patients without Baseline Pruritus Who Initiate Treatment with Oca Have Similar Risk of Developing Treatment-Emergent Pruritus as Those Who Are Not on Treatment with Oca. Hepatology 2024;80(Supplement 1):S1840-S1841. | SLR Update III |
|  | 134 | Victor D, Li J, Nair R, et al. The Effect of Obeticholic Acid on Inflammatory Markers and Fibrosis Scores in Poise Incomplete Responders: A Retrospective Review of Poise, a Phase 3 Trial of Obeticholic Acid for the Treatment of Primary Biliary Cholangitis. Hepatology 2024;80(Supplement 1):S720-S721. | SLR Update III |
|  | 135 | Gish RG, Li J, Gasink C, et al. FRI-023 - Effect of obeticholic acid on reduction and normalization of alanine aminotransferase and aspartate aminotransferase in the phase 3 POISE trial in primary biliary cholangitis, In EASL International Liver Congress, 2024. | SLR Update II |
| **Poupon 1996** | 136 | Poupon RE, Huet PM, Poupon R, et al. A randomized trial comparing colchicine and ursodeoxycholic acid combination to ursodeoxycholic acid in primary biliary cirrhosis. Hepatology 1996;24(5):1098-1103. | Original SLR |
| **RDG-1/PBC** | 137 | Hirschfield, G, Hejda, V, Makara, M, et al. THU-103 - Results from a phase2a trial with the CD80 antagonist RhuDex as a second line treatment for primary biliary cholangitis. In EASL International Liver Congress, 2024. | SLR Update II |
| **RESPONSE** | 138 | **Hirschfield, GM; Bowlus, CL; Mayo, MJ et al. A Phase 3 Trial of Seladelpar in Primary Biliary Cholangitis. N Engl J Med. 2024.*** | SLR Update I |
|  | 139 | Hirschfield, G; Bowlus, C; Mayo, M et al. 5002 \| Efficacy and safety of seladelpar in patients with primary biliary cholangitis in the response trial: A phase 3 international, randomized, placebo-controlled study 2023. AASLD 2023. | SLR Update I |
|  | 140 | Anonymous. Efficacy and Safety of Seladelpar in Patients With Primary Biliary Cholangitis and Compensated Cirrhosis in the Phase 3 Placebo-Controlled RESPONSE Trial. Gastroenterology & Hepatology 2024;20:2-3. | SLR Update III |
|  | 141 | Bowlus C, Yilmaz Y, Zigmond E, et al. Seladelpar and Reductions in Lipids in Patients with Primary Biliary Cholangitis with and without Statin Use in the Phase 3 Placebo-Controlled Response Study. Hepatology 2024;80(Supplement 1):S1824-S1826. | SLR Update III |
|  | 142 | Kowdley K, Yimam K, Kumar S, et al. Alkaline Phosphatase Changes With Seladelpar Across Subgroups Of Primary Biliary Cholangitis Patients In The Response Trial. Digestive and Liver Disease 2025;57(Supplement 1):S88. | SLR Update III |
|  | 143 | Kowdley K, Yimam K, Kumar S, et al. Alkaline Phosphatase Changes with Seladelpar across Subgroups of Primary Biliary Cholangitis Patients in the Response Trial. Hepatology 2024;80(Supplement 1):S1822-S1823. | SLR Update III |
|  | 144 | Kremer A, Levy C, Mayo M, et al. Attenuation, near Resolution, and Prevention of Pruritus in Patients with Primary Biliary Cholangitis Treated with Seladelpar: A Secondary Analysis of Patterns of Pruritus Change in the Response Trial. Hepatology 2024;80(Supplement 1):S520-S521. | SLR Update III |
|  | 145 | Villamil A, Younes Z, Bowlus C, et al. Efficacy and Safety of Seladelpar in Patients with Primary Biliary Cholangitis and Compensated Cirrhosis in the Phase 3 Placebocontrolled Response Trial. Hepatology 2024;80(Supplement 1):S517-S518. | SLR Update III |
|  | 146 | Hansen B, Carroll S, Zhou Y, et al. THU-286 Seladelpar treatment of patients with primary biliary cholangitis improves the GLOBE score and predicts improved transplant-free survival. Journal of Hepatology 2025;82:S312-S313. | SLR Update III |
| **Silveira 2017** | 147 | Silveira MG, Gossard AA, Stahler AC, et al. A Randomized, Placebo-Controlled Clinical Trial of Efficacy and Safety: Modafinil in the Treatment of Fatigue in Patients With Primary Biliary Cirrhosis. American Journal of Therapeutics 2017;24:e167-e176. | Original SLR |
| **Tanaka 2025 (NCT03092765)** | 148 | Tanaka A, Abe M, Namisaki T, et al. A placebo-controlled Phase 2 trial of E6011, anti-human fractalkine monoclonal antibody, in primary biliary cholangitis. Journal of Translational Autoimmunity 2025;10:100283. | SLR Update III |
| **TRANSFORM (NCT05014672)** | 149 | ClinicalTrials.gov. A Trial of Setanaxib in Patients With Primary Biliary Cholangitis (PBC) and Liver Stiffness (TRANSFORM; NCT05014672). Available at: https://clinicaltrials.gov/study/NCT05014672. [Accessed: 9th October 2025]. | SLR Update III |
| **Wolfhagen 1998** | 150 | Wolfhagen FH, van Hoogstraten HJ, van Buuren HR, et al. Triple therapy with ursodeoxycholic acid, prednisone and azathioprine in primary biliary cirrhosis: a 1-year randomized, placebo-controlled study. Journal of Hepatology 1998;29:736-42. | Original SLR |

Publications in bold indicate the primary publication for each study where multiple linked articles were included. *Hirschfield 2024 was found through targeted searches conducted in February 2024

**Supplementary Table 11.** Baseline patient characteristics

| **Study name** | **Treatment arm** | **Age, mean (SD), unless otherwise mentioned** | **Sex, female, %, unless otherwise stated** | **Race, White, %, unless otherwise stated** | **Disease duration, mean (SD), years, unless otherwise specified** | **Disease stage** | **Pruritus, %, unless otherwise stated** | **Fatigue, %, unless otherwise stated** | **ALP (Y/N)** | **Bilirubin (Y/N)** | **GGT (Y/N)** | **Albumin, mean, (Y/N)** | **AST (Y/N)** | **ALT (Y/N)** |
| --- | --- | --- | --- | --- | --- | --- | --- | --- | --- | --- | --- | --- | --- | --- |
| Askari 2010 | All patients | NR | n=26/28 | NR | NR | NR | NR | NR | Y | Y | NR | NR | Y | Y |
| BEZURSO (NCT01654731) | Bezafibrate + UDCA | 53 (9) | 98 | 94 | NR | Stage 1: 28%; Stage 2: 30%; Stage 3: 23%; Stage 4: 19% (Ludwig) | 32 | 58 | Y | Y | Y | Y | Y | Y |
|  | Placebo + UDCA | 53 (11) | 92 | 96 | NR | Stage 1: 37%; Stage 2: 29%; Stage 3: 12%; Stage 4: 22% (Ludwig) | 48 | 58 | Y | Y | Y | Y | Y | Y |
| Bowlus 2022 (NCT02955602) | Seladelpar 2 mg | 55.2 (9.6) | 100 | 90.9 | 9.3 (6.9) | NR | 63.6 | 76 | Y | Y | Y | Y | Y | Y |
|  | Seladelpar 5 mg | 57.5 (8.1) | 96.2 | 94.3 | 10 (7.0) | NR | 71.7 | 63 | Y | Y | Y | Y | Y | Y |
|  | Seladelpar 10 mg | 57.4 (9.7) | 90.9 | 89.1 | 9.4 (6.2) | NR | 70.9 | NR | Y | Y | Y | Y | Y | Y |
| COBALT (NCT02308111) | OCA 5 mg titrated to 10 mg | 53.4 (10.28) | 89.9 | 86.9 | NR | NR | NR | NR | Y | Y | Y | Y | Y | Y |
|  | Placebo | 53.9 (10.41) | 89.8 | 86.1 | NR | NR | NR | NR | Y | Y | Y | Y | Y | Y |
|  | All patients | 53.7 (10.33) | 89.8 | 86.5 | NR | NR | NR | NR | NR | NR | NR | NR | NR | NR |
| Combes 2005 | Methotrexate + UDCA (Stage I-II) | 50.3 (7.9) | 91.9 | NR | 3.1 (3.1) | NR | NR | NR | Y | Y | NR | Y | Y | Y |
|  | Placebo + UDCA (Stage I-II) | 50.3 (9) | 96.9 | NR | 3.1 (3) | NR | NR | NR | Y | Y | NR | Y | Y | Y |
|  | Methotrexate + UDCA (Stage III-IV) | 50.4 (9.1) | 92.9 | NR | 3.8 (3.7) | NR | NR | NR | Y | Y | NR | Y | Y | Y |
|  | Placebo + UDCA (Stage III-IV) | 54 (7.7) | 88.4 | NR | 4.2 (3.4) | NR | NR | NR | Y | Y | NR | Y | Y | Y |
| ELATIVE (NCT04526665) | Elafibranor 80 mg | 57.5 (8.4) | 94.4 | 94 | 7.9 (5.9) | NR | NR | NR | Y | Y | Y | NR | Y | Y |
|  | Placebo | 56.4 (9.3) | 98.1 | 87 | 8.3 (6.8) | NR | NR | NR | Y | Y | Y | NR | Y | Y |
|  | All patients | 57 (9) | 96 | 91 | 8 (6.2) | NR | 41 | NR | NR | NR | NR | NR | NR | NR |
|  | Elafibranor 80 mg, patients with moderate-to-severe pruritus at Baseline | 54.8 (9.2) | 90.9 | NR | NR | NR | 100 | NR | Y | NR | NR | NR | NR | NR |
|  | Placebo, patients with moderate-to-severe pruritus at Baseline | 52.9 (9.5) | 95.5 | NR | NR | NR | 100 | NR | Y | NR | NR | NR | NR | NR |
| ENHANCE (NCT03602560) | Seldapar 5-10 mg | 54.7 (9.7) | 92 | 93.3 | 8.3 (6.4) | NR | 74 | NR | Y | Y | Y | NR | Y | Y |
|  | Seldapar 10 mg | 55.6 (9.1) | 93 | 86.5 | 8.4 (6.4) | NR | 73 | NR | Y | Y | Y | NR | Y | Y |
|  | Placebo | 55.9 (8.2) | 98 | 92 | 8.4 (6.2) | NR | 66 | NR | Y | Y | Y | NR | Y | Y |
| EPICS (NCT03112681) | Saroglitazar 4 mg | 55.1 (8) | 100 | 84.6 | NR |  | 46.2 | NR | Y | Y | Y | NR | Y | Y |
|  | Saroglitazar 2 mg | 57.2 (9.7) | 92.9 | 92.9 | NR |  | 14.3 | NR | Y | Y | Y | NR | Y | Y |
|  | Placebo | 59.2 (7.3) | 100 | 100 | NR |  | 50 | NR | Y | Y | Y | NR | Y | Y |
| GLIMMER (NCT02966834) | Placebo | 54.4 (11.06) | 94 | 72 | 7.5 (5.5) | NR | 100 | NR | Y | Y | NR | NR | NR | NR |
|  | Linerixibat 20 mg QD | 58.5 (7.35) | 100 | 63 | 9.4 (6) | NR | 100 | NR | Y | Y | NR | NR | NR | NR |
|  | Linerixibat 90 mg QD | 52.5 (12.32) | 91 | 74 | 7.2 (6) | NR | 100 | NR | Y | Y | NR | NR | NR | NR |
|  | Linerixibat 180 mg QD | 58.9 (11.1) | 93 | 70 | 10.7 (9.3) | NR | 100 | NR | Y | Y | NR | NR | NR | NR |
|  | Linerixibat 40 mg BID | 55.6 (11.23) | 96 | 70 | 6.8 (6.4) | NR | 100 | NR | Y | Y | NR | NR | NR | NR |
|  | Linerixibat 90 mg BID | 56.2 (11.32) | 91 | 68 | 7.4 (5.4) | NR | 100 | NR | Y | Y | NR | NR | NR | NR |
| Gonzalez-Koch 1997 | Methotrexate + UDCA | 49 (8) | 100 | NR | NR | Stage 1: 23%; Stage 2: 38.5%; Stage 3: 38.5% | n=8; Denominator=13 | n=4; Denominator=13 | Y | Y | Y | Y | Y | Y |
|  | Placebo + UDCA | 52 (13) | 100 | NR | NR | Stage 1: 17%; Stage 2: 25%; Stage 3: 58% | n=7; Denominator=12 | n=4; Denominator=12 | Y | Y | Y | Y | Y | Y |
| HAART (NCT01614405) | TDC/FTC/LPRr | Median 53 | 80 | NR | 7^a^ | NR | NR | NR | Y | Y | NR | Y | Y | Y |
|  | Placebo | Median 50 | 87.5 | NR | 3 ^a^ | NR | NR | NR | Y | Y | NR | Y | Y | Y |
| Hegade 2017 | All patients | 52.9 (10.6) | 86 | NR | 5 (4.8) | NR | NR | NR | Y | Y | Y | Y | Y | Y |
| Hirschfield 2015 (NCT00550862) | Placebo + UDCA | 54.8 (8.5) | 95 | NR | NR | NR | NR | NR | Y | Y | Y | Y | Y | Y |
|  | OCA 10 mg + UDCA | 55.6 (9.3) | 100 | NR | NR | NR | NR | NR | Y | Y | Y | Y | Y | Y |
|  | OCA 25 mg + UDCA | 55.9 (8) | 94 | NR | NR | NR | NR | NR | Y | Y | Y | Y | Y | Y |
|  | OCA 50 mg + UDCA | 54 (9.7) | 93 | NR | NR | NR | NR | NR | Y | Y | Y | Y | Y | Y |
| Hirschfield 2021 | Budesonide + UDCA | 54 | 95 | NR | 7.4 ^a^ | NR | NR | NR | Y | Y | NR | NR | Y | Y |
|  | Placebo + UDCA | Median 51 | 100 | NR | 5 ^a^ | NR | NR | NR | Y | Y | NR | NR | Y | Y |
| Hosonuma 2015 | UDCA | Median 64 | NR | NR | NR | Stage 1: n=7; Stage 2: n=5; Stage 3: n=3 (Scheuer's) | NR | NR | Y | Y | Y | Y | Y | Y |
|  | Bezafibrate + UDCA | Median 64 | NR | NR | NR | Stage 1: n=6; Stage 2: n=3; Stage 4: n=3; Stage 4: n=1 (Scheuer's) | NR | NR | Y | Y | Y | Y | Y | Y |
| Ikeda 1996 | UDCA + colchicine | 57 (SE: 3) | 90 | NR | NR | Stage 4: 40% | NR | NR | Y | Y | NR | Y | Y | Y |
|  | UDCA | 64 (SE: 3) | 83 | NR | NR | Stage 4: 33% | NR | NR | Y | Y | NR | Y | Y | Y |
| Iwasaki 2008 | Bezafibrate + UDCA | 49.8 | n=10; Denominator=12 | NR | 7.5 ^a^ | Stage 1: n=6; Stage 2: n=4 (Scheuer's) | NR | NR | Y | Y | NR | NR | Y | Y |
|  | UDCA | 58.3 | n=9; Denominator=10 | NR | 8.3 ^a^ | Stage 1: n=5; Stage 2: n=4; Stage 3: n=1 (Scheuer's) | NR | NR | Y | Y | NR | NR | Y | Y |
| Jones 2017 (NCT02609048) | Placebo + UDCA | Median 56 | 92 | NR | 4^a^ | NR | n=4; Denominator=13 | NR | Y | Y | Y | Y | Y | Y |
|  | Seladelpar 50 mg + UDCA | Median 55 | 92 | NR | 7^a^ | NR | n=4; Denominator=13 | NR | Y | Y | Y | Y | Y | Y |
|  | Seladelpar 200 mg + UDCA | Median 57 | 100 | NR | 9^a^ | NR | n=5; Denominator=12 | NR | Y | Y | Y | Y | Y | Y |
| Kanda 2003 | Bezafibrate + UDCA | 56 (9) | NR | NR | NR | Stage 1: n=3; Stage 2: n=3; Stage 3: n=4; Stage 4: n=1 (Ludwig) | n=7; Denominator=11 | NR | Y | NR | Y | NR | Y | Y |
|  | UDCA alone | 56 (12) | NR | NR | NR | Stage 1: n=2; Stage 2: n=5; Stage 3: n=3; Stage 4: n=1 (Ludwig) | n=5; Denominator=11 | NR | Y | NR | Y | NR | Y | Y |
| Kurihara 2000 | UDCA 600 mg | 59.8 (11.2) | NR | NR | NR | Stage 1: n=5; Stage 2: n=6; Stage 3: n=1 (Scheuer's) | NR | NR | Y | NR | NR | NR | NR | Y |
|  | Bezafibrate + UDCA | 60.3 (6.5) | NR | NR | NR | Stage 1: n=4; Stage 2: n=6; Stage 3: n=1; Stage 4: n=1 (Scheuer's) | NR | NR | Y | NR | NR | NR | NR | Y |
| Li 2022 (ChiCTR1800020160) | Fenofibrate + UDCA | 51.5 (11.7) | 79.2 | NR | NR |  | NR | NR | Y | Y | Y | Y | Y | Y |
|  | UDCA | 50.4 (11.2) | 100 | NR | NR |  | NR | NR | Y | Y | Y | Y | Y | Y |
| Liberopoulos 2010 | Fenofibrate + UDCA | 55.3 (4.5) | n=5; Denominator=6 | NR | NR |  | NR | NR | Y | Y | Y | NR | Y | Y |
|  | UDCA alone | 54.8 (7.3) | n=3; Denominator=4 | NR | NR |  | NR | NR | Y | Y | Y | NR | Y | Y |
| Lim 1997 | Azathioprine, prednisone + UDCA | 54 | n=9; Denominator=12 | NR | NR | Stage 1: n=4; Stage 2: n=6; Stage 3: n=3 | NR | NR | Y | Y | NR | Y | Y | Y |
|  | Placebo + UDCA | 55 | n=11; Denominator=12 | NR | NR | Stage 1: n=7; Stage 2: n=2; Stage 3: n=1 | NR | NR | Y | Y | NR | Y | Y | Y |
| Mayo 2018 (NCT02026401) | Aldafermin (NGM282) 0.3 mg + UDCA | 56.6 (8.5) | 92.9 | 100 | NR | NR | NR | NR | Y | NR | NR | NR | NR | NR |
|  | Aldafermin (NGM282) 3 mg + UDCA | 56.5 (9.8) | 93.8 | 100 | NR | NR | NR | NR | Y | NR | NR | NR | NR | NR |
|  | Placebo + UDCA | 55.7 (12.7) | 86.7 | 100 | NR | NR | NR | NR | Y | NR | NR | NR | NR | NR |
| Nakai 2000 | UDCA 600 mg | 57 (11) | NR | NR | NR | Stage 1: n=4; Stage 2: n=6; Stage 3: n=1 (Scheuer's) | NR | NR | Y | Y | NR | NR | NR | NR |
|  | Bezafibrate + UDCA | 7 (9) | NR | NR | NR | Stage 1: n=7; Stage 2: n=2; Stage 3: n=1 (Scheuer's) | NR | NR | Y | Y | NR | NR | NR | NR |
| NCT01904058 | LUM001 10 mg + UDCA (Cohort A) | 54.7 (12.74) | 95.2 | NR | NR | NR | NR | NR | NR | NR | NR | NR | NR | NR |
|  | LUM001 20 mg + UDCA (Cohort B) | 53.5 (10.53) | 81 | NR | NR | NR | NR | NR | NR | NR | NR | NR | NR | NR |
|  | Placebo + UDCA (Cohort A) | 47.5 (8.14) | 100 | NR | NR | NR | NR | NR | NR | NR | NR | NR | NR | NR |
|  | Placebo + UDCA (Cohort B) | 55.8 (8.73) | 92.3 | NR | NR | NR | NR | NR | NR | NR | NR | NR | NR | NR |
| NCT02943447 | Cilofexor 100 mg | 54 (9.8) | n=28; Denominator=28 | n=26; Denominator=28 | NR | NR | NR | NR | NR | NR | NR | NR | NR | NR |
|  | Cilofexor 30 mg | 57 (6.3) | n=26; Denominator=30 | n=27; Denominator=30 | NR | NR | NR | NR | NR | NR | NR | NR | NR | NR |
|  | Placebo | 58 (5.9) | n=12; Denominator=13 | n=13; Denominator=13 | NR | NR | NR | NR | NR | NR | NR | NR | NR | NR |
| NCT03226067 | GKT137831 400 mg once daily | 56.8 (9.1) | 78.9 | 100.0 | 62.4 months ^a^ |  | NR | NR | NR | NR | NR | NR | NR | NR |
|  | GKT137831 400 mg twice daily | 55.6 (9.04) | 94.4 | 100.0 | 70.1 months^a^ |  | NR | NR | NR | NR | NR | NR | NR | NR |
|  | Placebo | 56.3 (9.19) | 94.6 | 97.3 | 129.1 months^a^ |  | NR | NR | NR | NR | NR | NR | NR | NR |
| NCT03394924 | EDP-305 1 mg | 57.4 (8.61) | 100 | 90.3 | NR |  | NR | NR | NR | NR | NR | NR | NR | NR |
|  | EDP-305 2.5 mg | 54.9 (10.92) | 96.4 | 96.4 | NR |  | NR | NR | NR | NR | NR | NR | NR | NR |
|  | Placebo | 56.9 (8.49) | 100 | 100 | NR |  | NR | NR | NR | NR | NR | NR | NR | NR |
| NCT03633227 | Placebo | 62.5 (9.1) | 83.3 | 91.7 | NR |  | NR | NR | NR | NR | NR | NR | NR | NR |
|  | OCA | 60.5 (10.19) | 60 | 100 | NR |  | NR | NR | NR | NR | NR | NR | NR | NR |
| POISE (NCT01473524) | OCA 10 mg | 56 (11) | 86 | 96 | 9 (7) |  | 60 | NR | Y | Y | Y | Y | Y | Y |
|  | OCA 5-10 mg | 56 (11) | 93 | 96 | 8 (6) |  | 53 | NR | Y | Y | Y | Y | Y | Y |
|  | Placebo | 56 (10) | 93 | 90 | 8 (5) |  | 64 | NR | Y | Y | Y | Y | Y | Y |
| Poupon 1996 | Colchicine + UDCA | 55 (SE: 2) | 89 | NR | 32 (4) months | Scheuer; reported as other categories | 49 | NR | Y | Y | Y | Y | NR | Y |
|  | Placebo plus UDCA 13-15 mg/kg-1 | 52 (SE: 2) | 81 | NR | 41 (6) months | Scheuer; reported as other categories | 38 | NR | Y | Y | Y | Y | NR | Y |
| RDG-1/PBC | Placebo | NR | NR | NR | NR | NR | NR | NR | NR | NR | NR | NR | NR | NR |
|  | RhuDex 50 mg | NR | NR | NR | NR | NR | NR | NR | NR | NR | NR | NR | NR | NR |
|  | RhuDex 100 mg | NR | NR | NR | NR | NR | NR | NR | NR | NR | NR | NR | NR | NR |
|  | RhuDex 200 mg | NR | NR | NR | NR | NR | NR | NR | NR | NR | NR | NR | NR | NR |
|  | All patients | 55.5 | 94 | NR | 5.4 | NR | NR | NR | Y | NR | NR | NR | NR | Y |
| RESPONSE | Placebo | 57 (9.2) | 92.3 | 86.2 | 8.6 (6.5) | NR | 73.8 | NR | Y | Y | Y | NR | Y | Y |
|  | Seladelpar 10 mg | 56.6 (10.0) | 96.1 | 89.1 | 8.2 (6.7) | NR | 71.1 | NR | Y | Y | Y | NR | Y | Y |
|  | All patients | 56.7 | 94.8 | NR | NR | NR | NR | NR | NR | NR | NR | NR | NR | NR |
| Schattenberg 2021 | Elafibranor 80 mg + UDCA | 56.5 (8.7) | n=14; Denominator=15 | NR | NR | NR | NR | NR | Y | Y | Y | Y | Y | Y |
|  | Elafibranor 120 mg + UDCA | 60.4 (6.9) | n=15; Denominator=15 | NR | NR |  | NR | NR | Y | Y | Y | Y | Y | Y |
|  | Placebo + UDCA | 60.5 (8.6) | n=14; Denominator=15 | NR | NR |  | NR | NR | Y | Y | Y | Y | Y | Y |
| Schramm 2022 | Tropifexor 30 μg + UDCA | 58.6 (12.42) | 100 | 90.9 | NR |  | 44.4 | NR | Y | Y | Y | NR | Y | Y |
|  | Tropifexor 60 μg + UDCA | 57.9 (11.21) | 77.8 | 88.9 | NR |  | 44.4 | NR | Y | Y | Y | NR | Y | Y |
|  | Tropifexor 90 μg + UDCA | 53.6 (7.42) | 100 | 100 | NR |  | 16.7 | NR | Y | Y | Y | NR | Y | Y |
|  | Tropifexor 150 μg + UDCA | 57.4 (13.81) | 100 | 100 | NR |  | 37.5 | NR | Y | Y | Y | NR | Y | Y |
|  | Placebo + UDCA | 53.7 (10.19) | 100 | 90.5 | NR |  | 50 | NR | Y | Y | Y | NR | Y | Y |
| Silveira 2017 | Modafinil 100 mg | Median 60 | 100 | NR | NR | Stage 1: 18.8%, Stage 2: 50%; Stage 3: 18.8%; Stage 4: 12.5% (Ludwig) | NR | NR | Y | Y | NR | Y | Y | NR |
|  | Placebo | Median 59 | 1 | NR | NR | Stage 1: 28.6%, Stage 2: 50%; Stage 3: 21.4% (Ludwig) | NR | NR | Y | Y | NR | Y | Y | NR |
| Study 213 | All patients | 55.4 (8.1) | 93.3 | NR | NR | NR | NR | NR | NR | NR | NR | NR | NR | NR |
|  | Bezafibrate 200 mg + placebo | 56.9 (9.1) | 89.5 | NR | NR | NR | NR | NR | Y | Y | NR | NR | NR | NR |
|  | Bezafibrate 200 mg + OCA | 55.9 (6.0) | 89.5 | NR | NR | NR | NR | NR | Y | Y | NR | NR | NR | NR |
|  | Bezafibrate 400 mg + placebo | 53.7 (8.6) | 94.7 | NR | NR | NR | NR | NR | Y | Y | NR | NR | NR | NR |
|  | Bezafibrate 400 mg + OCA | 55.1 (8.7) | 100 | NR | NR | NR | NR | NR | Y | Y | NR | NR | NR | NR |
| Study 213/Study 214 | All patients in Study 213 | NR | NR | NR | NR | NR | NR | NR | Y | Y | NR | NR | NR | NR |
|  | All patients in Study 214 | NR | NR | NR | NR | NR | NR | NR | Y | Y | NR | NR | NR | NR |
| Tanaka 2025 (NCT03092765) | All patients | 55 (12) | 90 | NR | 6.5 (7.9) | Stage 2: 3%; Stage 3: 14%; Stage 4: 83% | NR | NR | Y | Y | Y | NR | Y | Y |
|  | Placebo | 56 (13) | 86 | NR | 8.2 (9.4) | Stage 3: 14%; Stage 4: 86% | NR | NR | Y | Y | Y | NR | Y | Y |
|  | E6011 10 mg/kg/month | 58 (15) | 100 | NR | 12.8 (15.1) | Stage 3: 25%; Stage 4: 75% | NR | NR | Y | Y | Y | NR | Y | Y |
|  | E6011 15mg/kg/month | 45 (6) | 100 | NR | 3.1 (2.2) | Stage 4: 100% | NR | NR | Y | Y | Y | NR | Y | Y |
|  | E6011 10 mg/kg/eow | 56 (11) | 86 | NR | 4.9 (4.3) | Stage 2: 7%; Stage 3: 14%; Stage 4: 79% | NR | NR | Y | Y | Y | NR | Y | Y |
| TRANSFORM (NCT05014672) | Setanaxib 1200 mg | NR | 91.7 | 91.7 | NR | NR | NR | NR | Y | NR | NR | NR | NR | NR |
|  | Setanaxib 1600 mg | NR | 96.0 | 80.0 | NR | NR | NR | NR | Y | NR | NR | NR | NR | NR |
|  | Placebo | NR | 92.6 | 85.2 | NR | NR | NR | NR | Y | NR | NR | NR | NR | NR |
| Wolfhagen 1998 | Prednisone 10 mg, azathioprine 50 mg and UDCA (10 mg/kg per day) | 52.7 (8) | n=23; Denominator=26 | NR | NR | Stage 1: n=3; Stage 2: n=7; Stage 3: n=11; Stage 4: n=5(Ludwig) | NR | NR | Y | Y | Y | Y | Y | Y |
|  | Placebo + UDCA | 52 (10.8) | n=22; Denominator=24 | NR | NR | Stage 1: n=1; Stage 2: n=7; Stage 3: n=9; Stage 4: n=6 (Ludwig) | NR | NR | Y | Y | Y | Y | Y | Y |
| Xiang 2021 (NCT03345589) | UDCA 13-15 mg | 52^a^ | 83.3 | NR | NR | NR | 50 | 30.6 | Y | Y | Y | Y | Y | Y |
|  | UDCA 18-22 mg | 52^a^ | 89.2 | NR | NR | NR | 51.4 | 35.1 | Y | Y | Y | Y | Y | Y |

^a^Median values reported. ALP: alkaline phosphatase; ALT: alanine transaminase; AST: aspartate aminotransferase; GGT: Gamma-glutamyl transferase; NR: not reported; SD: standard deviation; UDCA: ursodeoxycholic acid; Y: yes, reported but not extracted.

**Supplementary Table 12.** Baseline treatment characteristics

| **Study** | **Treatment arm** | **UDCA, previous therapy (%), unless otherwise stated** | **OCA, previous therapy (%), unless otherwise stated** | **Fibrates, previous therapy (%), unless otherwise stated** | **Corticosteroids, previous therapy, (%), unless otherwise stated** | **Other** | **UDCA, lack of response in prior therapy , (%), unless otherwise stated** | **Intolerance to UDCA, (%), unless otherwise stated** | **Concomitant UDCA, (%), unless otherwise stated** | **Baseline UDCA dose, mean (SD), (mg/kg/day), unless otherwise stated** |
| --- | --- | --- | --- | --- | --- | --- | --- | --- | --- | --- |
| Askari 2010 | All patients | NR | NR | NR | NR | NR | NR | NR | 100 | NR |
| BEZURSO (NCT01654731) | Bezafibrate + UDCA | 100 | NR | NR | NR | NR | 100 | 0 | 100 | Median: 15 |
|  | Placebo + UDCA | 100 | NR | NR | NR | NR | 100 | 0 | 100 | Median: 15 |
| Bowlus 2022 (NCT02955602) | Seladelpar 2 mg | 100 | 0 | NR | NR | NR | NR | 0 | 100 | 13.6 (4) |
|  | Seladelpar 5 mg | 100 | 15.1 | NR | NR | NR | NR | 9.4 | 90.6 | 15.1 (3.2) |
|  | Seladelpar 10 mg | 100 | 12.7 | NR | NR | NR | NR | 5.5 | 94.5 | 15.1 (4.9) |
|  | Seladelpar 5 mg | 100 | NR | NR | NR | NR | NR | 8 | NR | 13 (3) |
|  | Seladelpar 10 mg | 100 | NR | NR | NR | NR | NR | 6 | NR | 15 (4) |
| COBALT (NCT02308111) | OCA 5 mg titrated to 10 mg | 95.8 | NR | NR | NR | NR | NR | NR | NR | NR |
|  | Placebo | 98.2 | NR | NR | NR | NR | NR | NR | NR | NR |
|  | All patients | NR | NR | NR | NR | NR | NR | NR | NR | NR |
| Combes 2005 | Methotrexate + UDCA (Stage I-II) | 100 | NR | NR | NR | NR | NR | NR | 100 | NR |
|  | Placebo + UDCA (Stage I-II) | 100 | NR | NR | NR | NR | NR | NR | 100 | NR |
|  | Methotrexate + UDCA (Stage III-IV) | 100 | NR | NR | NR | NR | NR | NR | 100 | NR |
|  | Placebo + UDCA (Stage III-IV) | 100 | NR | NR | NR | NR | NR | NR | 100 | NR |
| ELATIVE (NCT04526665) | Elafibranor 80 mg | NR | NR | NR | NR | NR | NR | NR | 94.4 | NR |
|  | Placebo | NR | NR | NR | NR | NR | NR | NR | 96.2 | NR |
|  | All patients | NR | NR | NR | NR | NR | NR | NR | NR | NR |
|  | Elafibranor 80 mg, patients with moderate-to-severe pruritus at Baseline | NR | NR | NR | NR | NR | NR | NR | NR | NR |
|  | Placebo, patients with moderate-to-severe pruritus at Baseline | NR | NR | NR | NR | NR | NR | NR | NR | NR |
| EPICS (NCT03112681) | Saroglitazar 4 mg | 100 | NR | NR | NR | NR | NR | NR | 100 | 11.6 (4) |
|  | Saroglitazar 2 mg | 100 | NR | NR | NR | NR | NR | NR | 85.7 | 15 (6.5) |
|  | Placebo | 100 | NR | NR | NR | NR | NR | NR | 100 | 14 (4.3) |
| ENHANCE (NCT03602560) | Seldapar 5-10 mg | 100 | 15 | 10 | NR | Steroids, immunosuppressants, methotrexate, systemic steroids, and cochicine | NR | NR | 93 | 15.6 (4.4) |
|  | Seldapar 10 mg | 100 | 18 | 7 | NR | Steroids, immunosuppressants, methotrexate, systemic steroids, and cochicine | NR | NR | 91 | 15.3 (3.7) |
|  | Placebo | 100 | 13 | 9 | NR | Steroids, immunosuppressants, methotrexate, systemic steroids, and cochicine | NR | NR | 98 | 15 (2.6) |
| GLIMMER (NCT02966834) | Placebo | NR | NR | NR | NR | NR | NR | NR | 89 | NR |
|  | Linerixibat 20 mg QD | NR | NR | NR | NR | NR | NR | NR | 88 | NR |
|  | Linerixibat 90 mg QD | NR | NR | NR | NR | NR | NR | NR | 96 | NR |
|  | Linerixibat 180 mg QD | NR | NR | NR | NR | NR | NR | NR | 100 | NR |
|  | Linerixibat 40 mg BID | NR | NR | NR | NR | NR | NR | NR | 81 | NR |
|  | Linerixibat 90 mg BID | NR | NR | NR | NR | NR | NR | NR | 95 | NR |
| Gonzalez-Koch 1997 | Methotrexate + UDCA | NR | NR | NR | NR | NR | NR | NR | 100 | NR |
|  | Placebo + UDCA | NR | NR | NR | NR | NR | NR | NR | 100 | NR |
| HAART (NCT01614405) | TDC/FTC/LPRr | 100 | NR | NR | NR | NR | 100 | NR | 100 | 13-15 |
|  | Placebo | 100 | NR | NR | NR | NR | 100 | NR | 100 | 13-15 |
| Hegade 2017 (NCT01899703) | All patients | 100 | NR | NR | NR | NR | NR | NR | 90.4 | 967 (185.8; units = mg/day) |
| Hirschfield 2015 (NCT00550862) | Placebo + UDCA | 100 | NR | NR | NR | NR | NR | NR | 100 | 15.9 (4.4) |
|  | OCA 10 mg + UDCA | 100 | NR | NR | NR | NR | NR | NR | 100 | 15.9 (4.1) |
|  | OCA 25 mg + UDCA | 100 | NR | NR | NR | NR | NR | NR | 100 | 15.6 (3.7) |
|  | OCA 50 mg + UDCA | 100 | NR | NR | NR | NR | NR | NR | 100 | 16.3 (5.2) |
| Hirschfield 2021 | Budesonide + UDCA | 100 | NR | NR | NR | NR | 100 | NR | 100 | 12-15 |
|  | Placebo + UDCA | 100 | NR | NR | NR | NR | 100 | NR | 100 | 12-15 |
| Hosonuma 2015 | UDCA | 100 | NR | NR | NR | NR | NR | NR | 100 | NR |
|  | Bezafibrate + UDCA | 100 | NR | NR | NR | NR | NR | NR | 100 | NR |
| Ikeda 1996 | UDCA + colchicine | 100 | NR | NR | NR | NR | NR | NR | 100 | NR |
|  | UDCA | 100 | NR | NR | NR | NR | NR | NR | 100 | NR |
| Iwasaki 2008 | Bezafibrate + UDCA | 100 | NR | NR | NR | NR | NR | NR | 100 | NR |
|  | UDCA | 100 | NR | NR | NR | NR | NR | NR | 100 | NR |
| Jones 2017 (NCT02609048) | Placebo + UDCA | 100 | NR | NR | NR | NR | NR | NR | 100 | 16 (2) |
|  | Seladelpar 50 mg + UDCA | 100 | NR | NR | NR | NR | NR | NR | 100 | 15 (3) |
|  | Seladelpar 200 mg + UDCA | 100 | NR | NR | NR | NR | NR | NR | 100 | 14 (2) |
| Kanda 2003 | Bezafibrate + UDCA | 100 | NR | NR | NR | NR | NR | NR | 100 | NR |
|  | UDCA alone | 100 | NR | NR | NR | NR | NR | NR | 100 | NR |
| Kurihara 2000 | UDCA 600 mg | NR | NR | NR | NR | NR | NR | NR | 100 | NR |
|  | Bezafibrate + UDCA | NR | NR | NR | NR | NR | NR | NR | 100 | NR |
| Li 2022 (ChiCTR1800020160) | Fenofibrate + UDCA | 100 | NR | NR | NR | NR | 100 | NR | 100 | NR |
|  | UDCA | 100 | NR | NR | NR | NR | 100 | NR | 100 | NR |
| Liberopoulos 2010 | Fenofibrate + UDCA | 100 | NR | NR | NR | NR | 100 | NR | 100 | NR |
|  | UDCA alone | 100 | NR | NR | NR | NR | 100 | NR | 100 | NR |
| Lim 1997 | Azathioprine, prednisone + UDCA | 100 | NR | NR | NR | NR | 100 | NR | 100 | NR |
|  | Placebo + UDCA | 100 | NR | NR | NR | NR | 100 | NR | 100 | NR |
| Mayo 2018 (NCT02026401) | Aldafermin (NGM282) 0.3 mg + UDCA | 100 | NR | NR | NR | NR | 100 | NR | 100 | 15.4 |
|  | Aldafermin (NGM282) 3 mg + UDCA | 100 | NR | NR | NR | NR | 100 | NR | 100 | 15.4 |
|  | Placebo + UDCA | 100 | NR | NR | NR | NR | 100 | NR | 100 | 15.9 |
| Nakai 2000 | UDCA 600 mg | NR | NR | NR | NR | NR | NR | NR | 100 | NR |
|  | Bezafibrate + UDCA | NR | NR | NR | NR | NR | NR | NR | 100 | NR |
| NCT01904058 | LUM001 10 mg + UDCA (Cohort A) | 100 | NR | NR | NR | NR | NR | NR | 100 | NR |
|  | LUM001 20 mg + UDCA (Cohort B) | 100 | NR | NR | NR | NR | NR | NR | 100 | NR |
|  | Placebo + UDCA (Cohort A) | 100 | NR | NR | NR | NR | NR | NR | 100 | NR |
|  | Placebo + UDCA (Cohort B) | 100 | NR | NR | NR | NR | NR | NR | 100 | NR |
| NCT02943447 | Cilofexor 100 mg | NR | NR | NR | NR | NR | NR | NR | NR | NR |
|  | Cilofexor 30 mg | NR | NR | NR | NR | NR | NR | NR | NR | NR |
|  | Placebo | NR | NR | NR | NR | NR | NR | NR | NR | NR |
| NCT03226067 | GKT137831 400 mg once daily | 100 | NR | NR | NR | NR | NR | NR | 100 | Median: 14.6 |
|  | GKT137831 400 mg twice daily | 100 | NR | NR | NR | NR | NR | NR | 100 | Median: 14.9 |
|  | Placebo | 100 | NR | NR | NR | NR | NR | NR | 100 | Median: 13.4 |
| NCT03394924 | EDP-305 1 mg | 100 | NR | NR | NR | NR | NR | NR | NR | NR |
|  | EDP-305 2.5 mg | 100 | NR | NR | NR | NR | NR | NR | NR | NR |
|  | Placebo | 100 | NR | NR | NR | NR | NR | NR | NR | NR |
|  | Placebo | NR | NR | NR | NR | NR | NR | NR | NR | NR |
|  | OCA | NR | NR | NR | NR | NR | NR | NR | NR | NR |
| POISE (NCT01473524) | OCA 10 mg | 100 | NR | NR | NR | NR | NR | NR | 92 | 16 (5) |
|  | OCA 5-10 mg | 100 | NR | NR | NR | NR | NR | NR | 93 | 17 (5) |
|  | Placebo | 100 | NR | NR | NR | NR | NR | NR | 93 | 15 (4) |
| Poupon 1996 | Colchicine + UDCA | 100 | NR | NR | NR | NR | NR | NR | 100 | 13-15 |
|  | Placebo plus UDCA 13-15 mg/kg-1 | 100 | NR | NR | NR | NR | NR | NR | 100 | 13-15 |
| RDG-1/PBC | Placebo | 100 | NR | NR | NR | NR | 100 | NR | NR | NR |
|  | RhuDex 50 mg | 100 | NR | NR | NR | NR | 100 | NR | NR | NR |
|  | RhuDex 100 mg | 100 | NR | NR | NR | NR | 100 | NR | NR | NR |
|  | RhuDex 200 mg | 100 | NR | NR | NR | NR | 100 | NR | NR | NR |
|  | All patients | 100 | NR | NR | NR | NR | 100 | NR | NR | NR |
| RESPONSE | Placebo | 100 | 15.4 | 7.7 | NR | OCA and/or fibrates, steroids, systemic steroids, methotrexate, other | NR | 6.2 | NR | 14.9 (3.3) |
|  | Seladelpar 10 mg | 100 | 11.7 | 5.5 | NR | OCA and/or fibrates, steroids, systemic steroids, methotrexate, other | NR | 6.2 | NR | 15 (3.1) |
|  | All patients | 100 | NR | NR | NR | NR | NR | NR | NR | NR |
| Schattenberg 2021 | Elafibranor 80 mg + UDCA | 100 | NR | NR | NR | NR | NR | NR | 100 | 14.3 (4.01) |
|  | Elafibranor 120 mg + UDCA | 100 | NR | NR | NR | NR | NR | NR | 100 | 13.5 (3.05) |
|  | Placebo + UDCA | 100 | NR | NR | NR | NR | NR | NR | 100 | 14.8 (2.14) |
| Schramm 2022 | Tropifexor 30 μg + UDCA | 100 | NR | NR | NR | NR | 100 | NR | 100 | 15.2 (3.21) |
|  | Tropifexor 60 μg + UDCA | 100 | NR | NR | NR | NR | 100 | NR | 100 | 13.7 (3.35) |
|  | Tropifexor 90 μg + UDCA | 100 | NR | NR | NR | NR | 100 | NR | 100 | 14.2 (4.55) |
|  | Tropifexor 150 μg + UDCA | 100 | NR | NR | NR | NR | 100 | NR | 100 | 16.8 (5.35) |
|  | Placebo + UDCA | 100 | NR | NR | NR | NR | 100 | NR | 100 | 14.9 (2.64) |
| Silveira 2017 | Modafinil 100 mg | 100 | NR | NR | NR | NR | NR | NR | NR | NR |
|  | Placebo | 100 | NR | NR | NR | NR | NR | NR | NR | NR |
| Study 213 | All patients | NR | NR | NR | NR | NR | NR | NR | NR | NR |
|  | Bezafibrate 200 mg + placebo | 100 | NR | NR | NR | Statin | NR | NR | NR | 17 (8) |
|  | Bezafibrate 200 mg + OCA | 100 | NR | NR | NR | Statin | NR | NR | NR | 15.1(4.3) |
|  | Bezafibrate 400 mg + placebo | 100 | NR | NR | NR | Statin | NR | NR | NR | 13.8 (2.9) |
|  | Bezafibrate 400 mg + OCA | 94.4 | NR | NR | NR | Statin | NR | NR | NR | 17.3 (10.4) |
|  | All patients | 98.7 | NR | NR | NR | Statin | NR | NR | NR | 15.8 (7.0) |
| Study 213/Study 214 | All patients in Study 213 | NR | NR | NR | NR | NR | NR | NR | NR | Median: 14.068 |
|  | All patients in Study 214 | NR | NR | NR | NR | NR | NR | NR | NR | Median: 11.364 |
| Tanaka 2025 (NCT03092765) | All patients | NR | NR | NR | NR | NR | NR | NR | NR | Reported as categorical; not extracted |
|  | Placebo | NR | NR | NR | NR | NR | NR | NR | NR | Reported as categorical; not extracted |
|  | E6011 10 mg/kg/month | NR | NR | NR | NR | NR | NR | NR | NR | Reported as categorical; not extracted |
|  | E6011 15mg/kg/month | NR | NR | NR | NR | NR | NR | NR | NR | Reported as categorical; not extracted |
|  | E6011 10 mg/kg/eow | NR | NR | NR | NR | NR | NR | NR | NR | Reported as categorical; not extracted |
| TRANSFORM (NCT05014672) | Setanaxib 1200 mg | NR | NR | NR | NR | NR | NR | NR | NR | NR |
|  | Setanaxib 1600 mg | NR | NR | NR | NR | NR | NR | NR | NR | NR |
|  | Placebo | NR | NR | NR | NR | NR | NR | NR | NR | NR |
| Wolfhagen 1998 | Prednisone 10 mg, azathioprine 50 mg and UDCA (10 mg/kg per day) | 100 | NR | NR | NR | NR | 100 | NR | 100 | 10 |
|  | Placebo + UDCA | 100 | NR | NR | NR | NR | 100 | NR | 100 | 10 |
| Xiang 2021 (NCT03345589) | UDCA 13-15 mg | 100 | NR | NR | NR | NR | 100 | NR | 100 | NR |
|  | UDCA 18-22 mg | 100 | NR | NR | NR | NR | 100 | NR | 100 | NR |

ALP: alkaline phosphatase; ALT: alanine transaminase; AST: aspartate aminotransferase; GGT: Gamma-glutamyl transferase; kg: kilogram; mg: milligram; NR: not reported; SD: standard deviation; UDCA: ursodeoxycholic acid.

**Supplementary Table 13**. ULN Values Across Studies

| **Study name** | **ALP** | **ALT** | **AST** | **TB** | **GGT** |
| --- | --- | --- | --- | --- | --- |
| ELATIVE | Women: 104 U/L  Men: 129 U/L | NR | NR | NR | NR |
| Schattenberg 2021 | Women: 104 U/L  Men: 129 U/L | NR | NR | NR | NR |
| ENHANCE | 150 U/L | 42 U/L | NR | 1.1 mg/dL | NR |
| Hirschfield 2021 | Women: 104 U/L  Men: 129 U/L | Normalisation | Women: 35 U/L  Men: 50 U/L | 14 | NR |
| RESPONSE | 116 U/L | 41 U/L | 34 U/L | NR | Women: 38 U/L  Men: 52 U/L |

ALP: alkaline phosphatase; ALT: alanine transferase; AST: aspartate aminotransferase; GGT: gamma glutamyltransferase; NR: not reported; TB: total bilirubin; U: units; UDCA: ursodeoxycholic acid; ULN: upper limit of normal.

**Supplementary Table 14.** Quality assessment

| **Study ID and name** | **Appropriate randomiza-tion** | **Adequate treatment allocation concealment** | **Blinding of participants and care providers** | **Blinding of outcome assessors** | **Similar prognostic factors at baseline** | **Imbalances in dropouts** | **Unreported outcomes** | **Use of ITT analysis** | **Declaration of conflicts of interest** |
| --- | --- | --- | --- | --- | --- | --- | --- | --- | --- |
| **Askari 2010** | PY | NI | **Y** | PY | PY | **N** | **N** | PY | **Y** |
| **BEZURSO (NCT01654731)** | **Y** | **Y** | **Y** | **Y** | PY | PN | **N** | **Y** | **Y** |
| **Bowlus 2022 (NCT02955602)** | **Y** | NA | N | N | PY | PN | **N** | **Y** | **Y** |
| **CLARITY (NCT01904058)** | NI | NI | **Y** | **Y** | PY | PN | **N** | PY | NR |
| **COBALT (NCT02308111)** | **Y** | NI | **Y** | NI | **Y** | **N** | **N** | PY | N |
| **Combes 2005** | **Y** | NI | **Y** | **Y** | PY | **N** | **N** | NI | NI |
| **ELATIVE (NCT04526665)** | **Y** | NI | **Y** | **Y** | **Y** | **N** | **N** | **Y** | **Y** |
| **ENHANCE (NCT03602560)** | **Y** | **Y** | PY | **Y** | PY | **N** | **N** | PY | **Y** |
| **EPICS (NCT03112681)** | **Y** | **Y** | **Y** | **Y** | **Y** | PY | **N** | **Y** | **Y** |
| **FITCH (NCT02701166)** | **Y** | **Y** | **Y** | **Y** | NI | **N** | **N** | PY | **Y** |
| **GLIMMER (NCT02966834** | NI | NI | **Y** | **Y** | PY | **N** | PN | **Y** | **Y** |
| **Gonzalez-Koch 1997** | NI | NI | **Y** | **Y** | **Y** | **N** | **N** | NI | NR |
| **HAART (NCT01614405)** | NI | NI | **Y** | **Y** | **Y** | **Y** | **N** | N | **Y** |
| **Hegade 2017 (NCT01899703)** | **Y** | PY | **Y** | **Y** | NI | **N** | PN | NI | **Y** |
| **Hejda 2023** | NI | NI | NI | NI | NI | PY | **N** | NI | NR |
| **Hirschfield 2015 (NCT00550862)** | **Y** | **Y** | PY | **Y** | **Y** | PY | **N** | **Y** | **Y** |
| **Hirschfield 2021 (NCT00746486)** | **Y** | **Y** | **Y** | **Y** | PN | PY | **N** | **Y** | **Y** |
| **Hosonuma 2015** | NI | NA | N | N | **Y** | **N** | **N** | NI | NI |
| **Ikeda 1996** | NI | NI | PN | PN | **Y** | **N** | **N** | NI | **Y** |
| **Iwasaki 2008** | PY | **Y** | N | N | PY | NI | **N** | NI | **Y** |
| **Jones 2017 (NCT02609048)** | **Y** | **Y** | **Y** | **Y** | PY | **N** | **N** | PY | **Y** |
| **Kanda 2003** | NI | NI | PN | PN | **Y** | **N** | **N** | NI | N |
| **Kurihara 2000** | NI | NI | PN | PN | **Y** | **N** | **N** | NI | NI |
| **Li 2022 (ChiCTR1800020160)** | PY | NA | N | N | PY | **N** | **N** | **Y** | **Y** |
| **Liberopoulos 2010** | NI | NI | PN | PN | **Y** | **N** | **N** | **Y** | **Y** |
| **Lim 1997** | NI | NI | PN | PN | PY | **N** | **N** | PY | NR |
| **Mayo 2018** | **Y** | **Y** | **Y** | **Y** | PY | PY | **N** | N | **Y** |
| **Nakai 2000** | NI | NI | PN | PN | **Y** | **N** | **N** | NI | **Y** |
| **NCT02943447** | **Y** | **Y** | PY | **Y** | PY | **N** | **N** | **Y** | **Y** |
| **NCT03226067** | **Y** | **Y** | **Y** | **Y** | PY | **N** | **N** | **Y** | **Y** |
| **NCT03394924** | **Y** | **Y** | **Y** | **Y** | PY | PY | **N** | PY | **Y** |
| **NCT03633227** | NI | NI | **Y** | **Y** | PY | PN | **N** | **Y** | **Y** |
| **POISE (NCT01473524)** | **Y** | **Y** | **Y** | **Y** | **Y** | **N** | PY | PY | **Y** |
| **Poupon 1999** | **Y** | **Y** | PY | **Y** | **Y** | **N** | PY | **Y** | **Y** |
| **RDG-1/PBC** | NI | **Y** | **Y** | **Y** | NI | PY | NI | PY | NR |
| **RESPONSE (NCT04620733)** | **Y** | **Y** | PY | **Y** | PY | **N** | **N** | **Y** | **Y** |
| **Schattenberg 2021** | **Y** | **Y** | **Y** | **Y** | PY | **N** | **N** | PY | **Y** |
| **Schramm 2022 (NCT02516605)** | **Y** | **Y** | **Y** | **Y** | PY | PN | **N** | PY | **Y** |
| **Silveira 2017** | **Y** | **Y** | **Y** | **Y** | PY | PN | **N** | **Y** | **Y** |
| **Study 213/Study 214** | NI | NI | **Y** | NI | NI | PN | **N** | NI | NR |
| **Tanaka 2025 (NCT03092765)** | NI | NI | **Y** | Y | Y | N | **N** | N | Y |
| **TRANSFORM (NCT05014672)** | NI | NI | **Y** | NI | NI | N | **N** | Y | Y |
| **Wolfhagen 1998** | NI | NI | **Y** | **Y** | **Y** | PN | **N** | PY | **Y** |
| **Xiang 2021 (NCT03345589)** | NI | NA | N | N | **Y** | **N** | **N** | N | **Y** |

The quality of included studies was accessed in line with the University of York CRD quality assessment tool by one individual and verified by a second independent reviewer. CRD: Centre for Reviews and Dissemination; N: no; NA: not applicable; NI: no information; PN: partly no; PY: partly yes; ITT: intention to treat; RCT: randomized controlled trial; Y: yes.
